# Transcriptome analysis of historic olives reveals stress-specific biomarkers

**DOI:** 10.3389/fpls.2025.1549305

**Published:** 2025-06-05

**Authors:** Hamad A. Alkhatatbeh, Monther T. Sadder, Nizar Haddad, Ibrahim Al-Amad, Mohammad Brake, Nawal A. Alsakarneh, Abdulsalam M. Alnajjar

**Affiliations:** ^1^ Faculty of Agricultural Technology, Al-Balqa Applied University, Al-Salt, Jordan; ^2^ Department of Horticulture and Crop Science, School of Agriculture, University of Jordan, Amman, Jordan; ^3^ Olive Research Department, National Agricultural Research Center, Baqa, Jordan; ^4^ Innovations and Business Development, Fresh Del Monte, De L’Ora Bio, Amman, Jordan; ^5^ Department of Science, Faculty of Science, Jerash University, Jerash, Jordan; ^6^ Department of Nutrition and Food Processing, Al-Huson University College, Al-Balqa Applied University, Irbid, Jordan

**Keywords:** olive, salinity, drought, DEG, biomarkers

## Abstract

**Introduction:**

Water scarcity and soil salinization are increasingly becoming limiting factors in food production, including olives, a major fruit crop in several parts of the world. Investigating historical olives, which are the last resort for genetic resources, is essential due to their natural resilience to drought and salinity, making them valuable for breeding stress-tolerant cultivars and ensuring sustainable olive production.

**Methods:**

In this study, four historic olive cultivars (‘Nabali’, ‘Mehras’, ‘Frantoio’, and ‘Manzanillo’) were investigated under both drought and salinity stresses. These cultivars also preserve local biodiversity, support traditional agriculture, and offer economic opportunities through unique, heritage-based olive oils. Drought and salt stress in olives are assessed through physiological [the ratio of variable to maximum fluorescence (Fv/Fm), relative water content (RWC)], biochemical (proline content), and molecular (stress-responsive genes) analyses to evaluate stress tolerance.

**Results:**

Under salinity and drought stress, RWC decreased in all olive cultivars, with drought having the most severe impact. ‘Nabali’ exhibited the highest salinity tolerance, while all cultivars showed similar sensitivity to drought. Proline levels remained stable in ‘Mehras’ but decreased under salinity stress in ‘Frantoio’, ‘Manzanillo’, and ‘Nabali’. Higher proline accumulation under drought suggested better drought tolerance than salinity in these cultivars. Photosynthetic efficiency (Fv/Fm) declined under salinity and drought stress in all cultivars, with drought causing a more significant reduction. ‘Manzanillo’ showed the highest sensitivity to drought, while the other cultivars maintained moderate efficiency under stress. ‘Manzanillo’ and ‘Mehras’ exhibited the highest number of differentially expressed genes (DEGs) under both drought and salinity stress, with ‘Manzanillo’ showing 2,934 DEGs under drought and 664 under salinity stress, while ‘Mehras’ had 2,034 and 2,866 DEGs, respectively. ‘Nabali’ demonstrated the strongest salinity-specific response, with 3,803 DEGs under salinity stress compared to 1,346 under drought. ‘Frantoio’ consistently had the lowest number of DEGs, with 345 under drought and 512 under salinity stress, indicating a more stable transcriptional response. Comparative analyses between drought and salinity conditions revealed significant variations, with ‘Manzanillo’ showing 2,599 unique DEGs under drought relative to salinity stress, while ‘Nabali’ exhibited 2,666 DEGs under salinity stress relative to drought. The major novel upregulated genes under salinity stress were Xyloglucan endotransglucosylase hydrolase (7 fold in ‘Nabali’ and 6.9 fold in ‘Mehras’). The novel drought genes detected in ‘Frantoio’ included Phytosulfokines 3 (4.9 fold), while Allene oxide synthase (6.5 fold) and U-box domain-containing (6.4 fold) were detected in ‘Manzanillo’.

**Discussion:**

The data revealed both novel and common stress-specific biomarkers under both salinity and drought stress, which can potentially be utilized in olive breeding and genetic improvement programs to mitigate stress.

## 1 Introduction

The olive tree (*Olea europaea* L.) belongs to the Oleaceae family, which comprises 25 genera and approximately 600 species (Ben Ahmed et al, 2009). It is estimated to occupy 10.8 million ha spread across 58 countries, with 97% of it still concentrated in the Mediterranean basin (Benítez-Cabello et al., 2023). *O. europaea* L., an evergreen plant of medium size, can grow up to 10 meters high (Iaria, 2010). Olives are also considered one of the most important types of xerophyte species that have evolved to withstand the climate in the Mediterranean region, which is considered a harsh climate. The *O. europaea* complex includes wild and cultivated Mediterranean olives (*O. europaea* subspecies *europaea*), and five non-Mediterranean subspecies: subsp. *laperrinei*; subsp. *cuspidata*; subsp. *guanchica*; subsp. *maroccana*; and subsp. *cerasiformis*.

The subsp. *europaea* is further subdivided into two taxonomic varieties: var. *sylvestris*, also named oleaster or wild olive, which comprises the wild forms of the olive tree, and var. *europaea*, which encompasses approximately 1,000 cultivated forms (Julca et al., 2023). Studies showed huge genetic variation between olive cultivars around the Mediterranean (Ateyyeh and Sadder, 2006; Ziar et al., 2024), indicating long-term adaptation to regional conditions.

Climate change poses a significant threat to global agricultural productivity by increasing the frequency and intensity of abiotic stresses such as drought and salinity (Masson-Delmotte et al, 2019; Muluneh, 2021). These stressors can disrupt cellular functions, reducing plant biomass, crop yield, and survival rates (Bose et al, 2014; Ma et al., 2020). Among these stresses, water scarcity is particularly critical, as water is essential for plant physiological processes, comprising 80%–90% of non-woody plant biomass and approximately 50% of woody plant biomass (Saheli Lisar et al., 2012).

Water deficit conditions induce morphological, physiological, and biochemical changes, affecting vital processes such as photosynthesis, respiration, and enzyme activity (Michaletti et al, 2018; Guo et al., 2023). In olives, drought stress leads to stomatal closure, reduced chlorophyll content, and decreased photosynthetic efficiency (Silva et al, 2018; Dias et al, 2022). Similarly, salinity stress increases the production of reactive oxygen species (ROS), causing oxidative damage, lipid peroxidation, and declines in stomatal conductance and chlorophyll levels (Chartzoulakis, 2005; Ben-Gal, 2011; Regni et al, 2019).

Despite these challenges, olive trees have evolved adaptive strategies to cope with arid conditions. Their xerophytic traits, including small, thick, waxy leaves and deep root systems, minimize water loss and enhance drought tolerance (Tripepi et al., 2011; Petruccelli et al., 2022). However, different olive cultivars exhibit varying degrees of stress tolerance. For instance, Frantoio, Picual, and Koroneiki are known for their resilience to environmental stressors, while Manzanillo is classified as moderately sensitive to drought (Gholami et al., 2022). Studies indicate that drought stress causes the greatest reduction in fruit size and yield in Manzanillo compared to other cultivars (Rico et al, 2023). In Jordan, where climatic conditions vary widely from north to south, olive cultivation plays a crucial role in agricultural production. The country hosts over 27 olive cultivars, including 13 local and 14 introduced varieties, each with distinct adaptive traits (Al-Qinna et al, 2011).

Given the increasing challenges posed by climate change, understanding the genetic and physiological mechanisms underlying stress tolerance in olives is crucial for developing resilient cultivars. While modern breeding programs often focus on widely cultivated varieties, historical olive cultivars represent a valuable reservoir of genetic diversity that has evolved under diverse environmental conditions. These cultivars may harbor unique adaptive traits that can be leveraged to enhance stress tolerance in future breeding efforts.

This study aims to explore the genetic and physiological responses of selected historical olive cultivars to drought and salinity stress. Specifically, we investigate the differential expression of stress-related genes and key physiological parameters, including photosynthetic efficiency, proline content, and relative water content (RWC). By integrating transcriptomic and physiological analyses, we seek to identify unique and common stress-responsive biomarkers that could inform breeding and genetic improvement programs. Our research hypothesizes that historical olive cultivars possess distinct genetic and physiological adaptations to abiotic stress, making them valuable candidates for future breeding strategies aimed at enhancing climate resilience in olive cultivation.

## 2 Materials and methods

### 2.1 Materials and experimental design

Four major historic olive cultivars were selected to study the biodiversity among them. Rooted plantlets were obtained from the National Agricultural Research Center (NARC), Jordan. The four cultivars are “Mehras” (Haddad et al., 2021; Sadder et al., 2023; Ziar et al., 2024), “Nabali” (one of the most widespread cultivars in Jordan), “Manzanillo” (the most widely cultivated variety in Spain), and “Frantoio” (the most noted olive oil variety in Tuscany, Italy). Plants that were 2 years old and approximately 1.2–1.5 m tall were grown in a greenhouse after transplanting into 15 L pots in growing media rich in organic matter (1:1:1 peatmoss: soil: sand). Three weeks after transplanting, olive plants (five plants per treatment for each cultivar) were subjected to the following treatments. (1) Drought stress (_d): Plants were irrigated with a polyethylene glycol (PEG 6000) solution added to the potting medium at concentrations of 300 g/L (1.03 MPa), applied once (Baccari et al., 2016; Du et al., 2024). (2) Salinity stress (_s): Plants were irrigated with a NaCl solution at a concentration of 75 mM (approximately 7.5 dS/m), applied 10 times (twice a week). (3) Control (_c): Plants were irrigated with regular tap water (EC = 0.7 dS/m) (Sadder et al., 2021; Boussadia et al., 2023).

### 2.2 Physiological parameters

#### 2.2.1 Relative water content

Mature and fully expanded leaves were cut and immediately placed into pre-weighed plastic tubes. The method of Parri et al. (2023) was applied and the RWC was calculated using the following equation:


RWC=(fresh weight−dry weight)÷(saturated weight−dry weight)×100%

#### 2.2.2 Proline content

For the determination of proline content, the method of Bates et al. (1973) was applied based on the standard curve for known concentrations of L-proline.

#### 2.2.3 Photosynthetic efficiency

The photosynthetic efficiency was estimated by measuring transient chlorophyll fluorescence using a Handy PEA 2000 fluorimeter (Hansatech Instruments, King’s Lynn, Norfolk, UK) with an excitation light energy of 3,000 μmol m^-^² s^-^¹, following the manufacturer’s instructions. The variation in photosynthesis activity was calculated as the ratio of variable to maximum fluorescence (Fv/Fm), where Fv = Fm - Fo.

#### 2.2.4 Statistical analysis

Means and standard deviations (SD) for physiological parameters were collected from five replicates (individual trees), each with three samples (leaves). The experimental design was completely randomized design (CRD) and data were analyzed with one-way ANOVA. Means were separated using least significant difference (LSD) at p < 0.05% level.

### 2.3 RNA extraction

Total RNA was extracted from plant leaf tissues from all four cultivars from all treatments (_d, _s, and _c) using the GF-1 Vivantis Total RNA Extraction Kit. Three biological replicates were taken for each sample. The extraction protocol was conducted as per the manufacturer’s instruction manual. The RNA samples were transferred to a new 1.5-ml RNase-free tube. The RNase inhibitor was added to RNA samples and stored at -80°C.

### 2.4 Full cDNA synthesis

RNA was used to synthesize the full-length double-stranded cDNA of the transcriptome for RNA sequencing. The reaction was conducted using a SMART^®^ cDNA Library Construction Kit according to the manufacturer’s instruction manual (SMARTer cDNA Synthesis, Takara Bio, USA) and using the PCR Program Smarter (cDNA) Kit (Applied Biosystems, Thermo Fisher Scientific, United States). After the run was completed, the full cDNA samples were stored at -20°C.

### 2.5 RNA sequencing and data analysis of transcriptome

cDNA was generated as described above using the SMARTer™ PCR cDNA Synthesis Kit (Clontech, USA). The quality of the RNA was checked using agarose gel electrophoresis and the absorbance was read using a Nanodrop 2000c spectrophotometer (Thermo Fisher Scientific, DE, USA). The samples were sent abroad for sequencing. The samples were prepared according to the NGS library preparation workflow and sequenced using the Illumina platform with paired-end and 100 bp long reads.

The RNA sequencing was analyzed using CLC Genomics Workbench v. 9.5 (Qiagen, USA). To normalize for sequencing depth and gene length, reads per kilobase of exon model per million mapped reads (RPKM) were calculated. DEGs with a p-value ≤ 0.05 were selected. CLC Genomics Workbench uses Student’s t-test as the statistical model to calculate the p-values for each DEG between two or more conditions. In addition, the software automatically adjusts the p-values using the Benjamini–Hochberg method to control the false discovery rate (FDR). Gene Ontology analysis for each DEG for each cultivar under each condition was performed using Blast2Go v. 6.3 (BioBam Bioinformatics, Spain).

### 2.6 Estimation of gene expression level using qPCR

A group of random DEGs from the transcriptome analysis was selected for confirmation using quantitative real-time PCR (qPCR) (**Supplementary Table 1**). The primer pairs for these genes were designed using Vector NTI 10 (Invitrogen, USA). The features were set in the program for primers as follows: primer length, 22–28 bp; Tm (°C), 58–60; GC %, 60; amplicon product length, 150–200 bp; and 3′ ends, CG. qPCR was performed using an Applied Biosystems™ 7500 Real-Time PCR System with SYBR Green dye. The reaction mixture for each sample had a total volume of 25 µL, consisting of 8 µL forward primer, 8 µL reverse primer, 6 µL cDNA, and 3 µL nuclease-free water. The qRT-PCR reactions were set up on a 96-well plate to ensure the accuracy and reproducibility of the results. The primers were designed to target specific genes of interest across four olive cultivars, with two actin primers serving as internal controls for normalization. The reaction conditions and cycling parameters were optimized according to the manufacturer’s instructions for the ABI system. The relative expression levels of the target genes were calculated using the 2^-ΔΔCt^ method (Sadder and Al-Doss, 2014).

## 3 Results

### 3.1 Physiological parameters

The two investigated abiotic stresses (salinity and drought) needed to be verified as effective, which was achieved by measuring the major physiological parameters, including relative water content, proline content, and photosynthesis efficiency. These parameters not only show the effectiveness of applied stresses but also provide a measurement of the tolerance of the investigated historic olive cultivars.

#### 3.1.1 Relative water content

**Figure 1:**
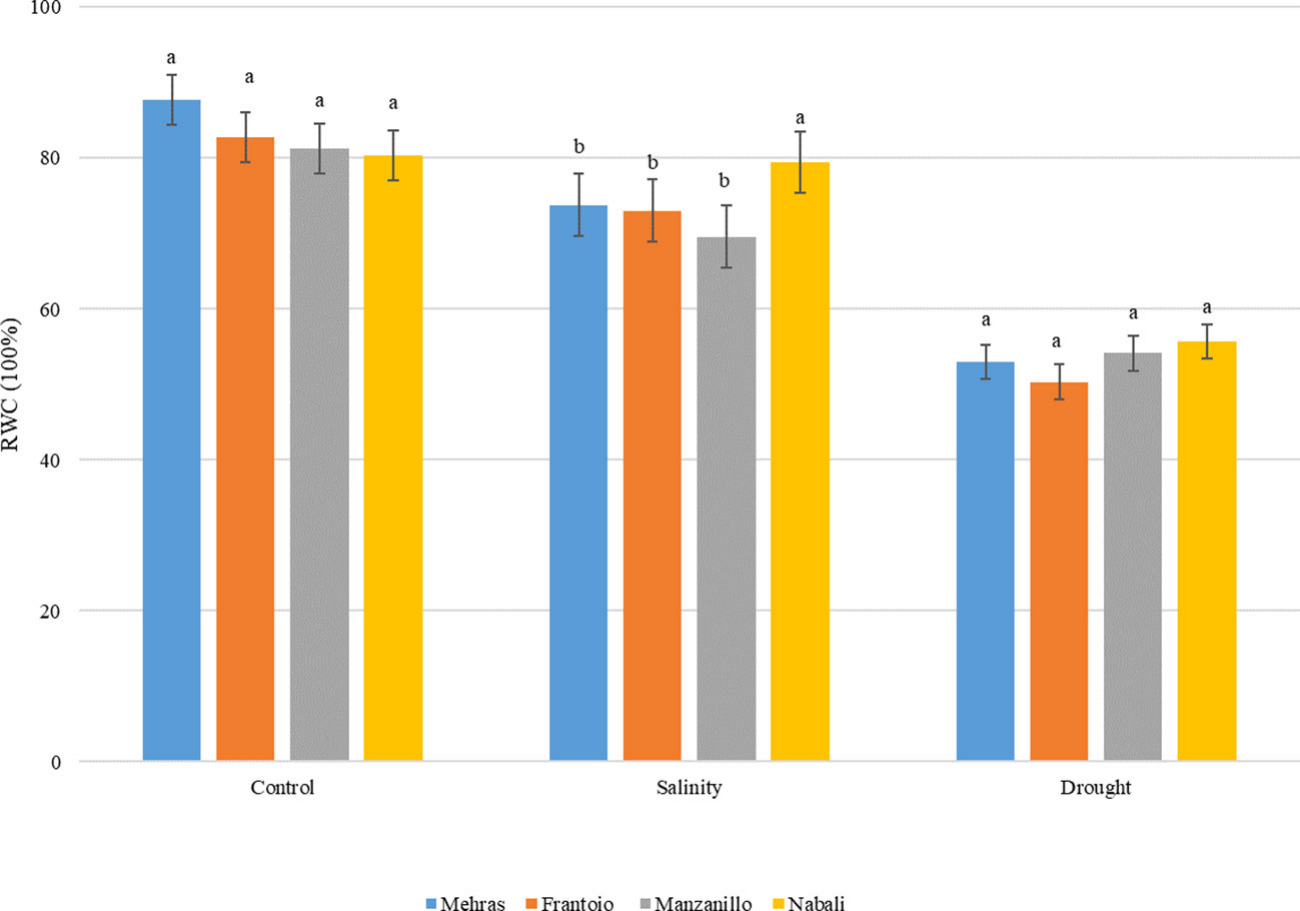
Analysis of RWC in leaves from four olive cultivars under salinity and drought stress. For each treatment, cultivars with different letters are significantly different at p < 0,05 using the LSD. Error bars represent the SD.

In this study, the RWC of four olive cultivars, Mehras, Frantoio, Manzanillo, and Nabali, was assessed under control, salinity, and drought stress conditions (**Figure 1**). Under control conditions, all four olive cultivars exhibited high RWC values, indicating that the plants were well-hydrated and not experiencing water stress. Mehras showed an RWC of approximately 87.7%, Frantoio of approximately 82%, Manzanillo of approximately 81%, and Nabali of approximately 80%. The LSD value was 7.51. Under salinity stress, the RWC values for all cultivars decreased, indicating water stress due to saline conditions. Mehras showed an RWC of approximately 73.8%, Frantoio of approximately 73%, and Manzanillo of approximately 69.5%. However, Nabali maintained a higher RWC of approximately 79.4%, similar to the control conditions, suggesting better salinity tolerance in Nabali compared to the other cultivars. The LSD value was 5.35. Under drought conditions, the RWC values for all cultivars also decreased, reflecting the impact of water deficit. Mehras showed an RWC of approximately 53%, Frantoio of approximately 50%, Manzanillo of approximately 54%, and Nabali of approximately 55%. These values indicated no statistically significant differences in RWC among the four cultivars under drought conditions with an LSD value of 8.13 (**Figure 1**).

**Figure 2:**
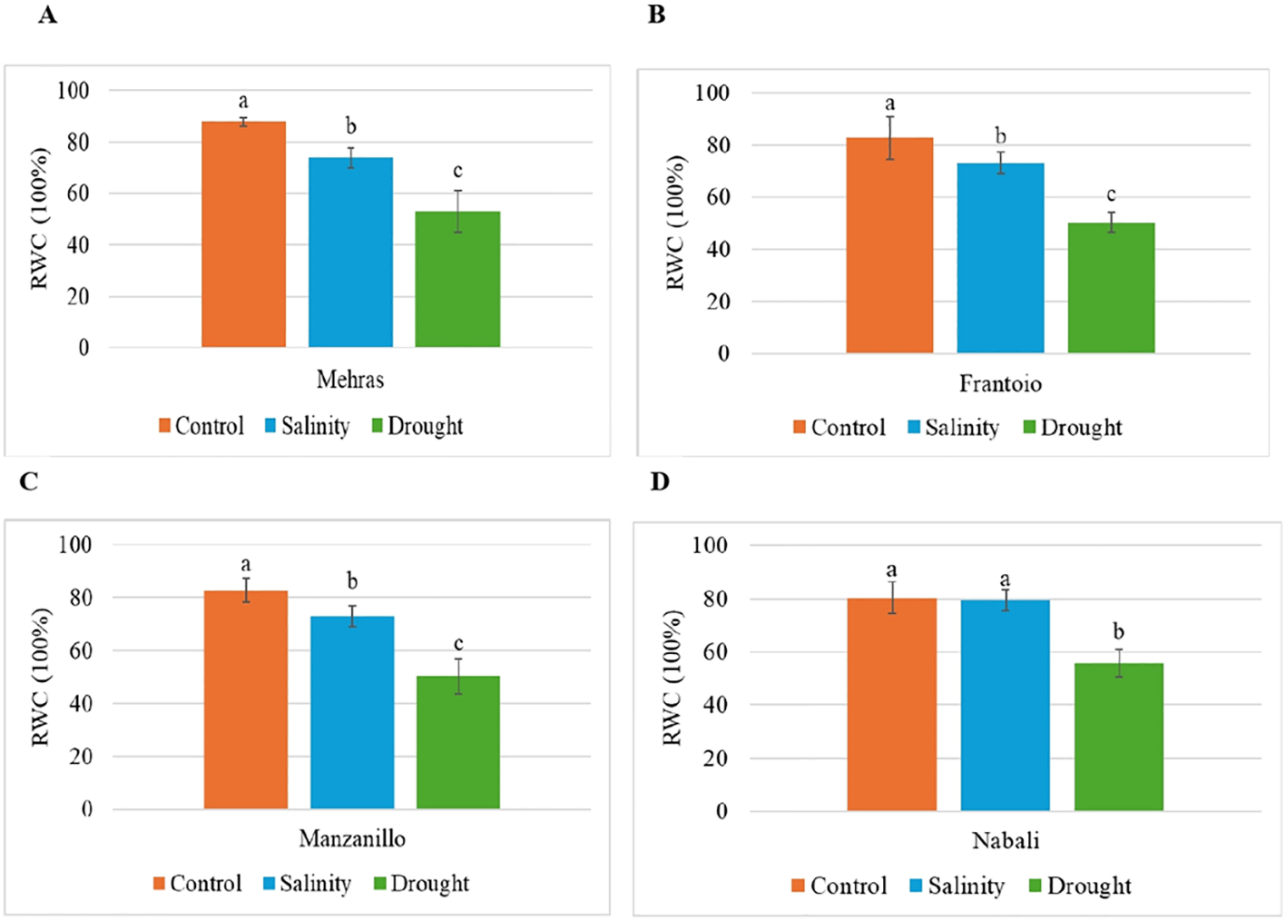
Analysis of RWC in olive cultivars under different stress conditions. Columns with the same letter are not significantly different using the LSD. Error bars represent the SD. **(A)** Mehras, **(B)** Frantoio, **(C)** Manzanillo and **(D)** Nabali olive cultivars.

The bar chart (**Figure 2A**) illustrates the RWC of the Mehras olive cultivar under control, salinity, and drought conditions. The LSD value was 6.89. Under the control conditions, the Mehras cultivar showed a high RWC of approximately 87.78%. This indicated that the plant maintained optimal water levels without any stress. In contrast, under salinity and drought conditions, the Mehras cultivar exhibited significantly lower RWC values of approximately 73.81% and 53.03%, respectively. This indicated that there was a significant decline in water content compared to the control condition (**Figure 2A**). Similar trends were found for both the Frantoio (**Figure 2B**) and Manzanillo (**Figure 2C**) olive cultivars.

However, the bar chart (**Figure 2D**) illustrates how the RWC of the Nabali olive cultivar changed under control, salinity, and drought conditions. Under both the control and salinity conditions, the Nabali cultivar maintained a high RWC, which showed that the plant kept its water content at an optimal level under normal conditions and was quite resilient to salinity stress. The LSD value was 7.35. Interestingly, the RWC under salinity stress was almost identical to the control condition, indicating that salinity did not significantly affect the water retention ability of the Nabali cultivar. This resilience suggested that the Nabali cultivar could tolerate saline conditions without compromising its water content (**Figure 2D**). However, the scenario changed under drought conditions. The RWC dropped significantly, which indicated that drought stress had a notable impact on the plant’s water retention. The difference between the control/salinity conditions and the drought conditions was quite pronounced, highlighting the greater sensitivity of the Nabali cultivar to drought compared to salinity (**Figure 2D**).

#### 3.1.2 Proline content

**Figure 3:**
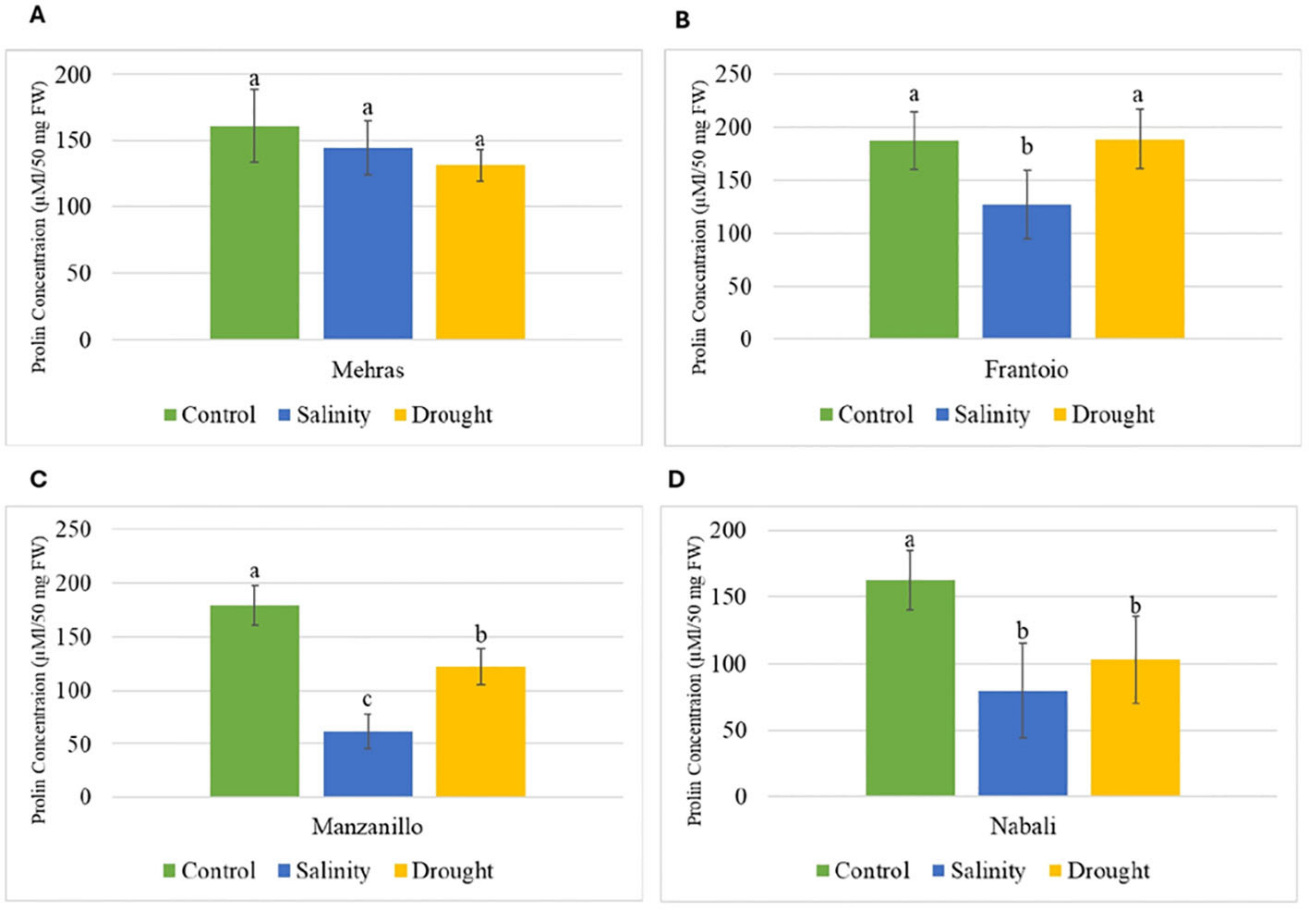
Analysis of proline content in olive cultivars under different stress conditions. Columns with the same letter are significantly not different using LSD. Error bars represent SD. **(A)** Mehras, **(B)** Frantoio, **(C)** Manzanillo and **(D)** Nabali olive cultivars.

Proline is an amino acid that accumulates in plants in response to various stress conditions, and its concentration can indicate the level of stress tolerance in different cultivars. The bar chart in **Figure 3A** displays the proline concentration (μM/50 mg FW) in the Mehras olive cultivar under three different conditions: control, salinity, and drought. The proline concentration was approximately 130–160 μM/50 mg FW (**Figure 3A**). The analysis of proline concentration in the Mehras olive cultivar under different stress conditions revealed no significant differences among the three conditions. However, for Frantoio, the proline concentration was approximately 180 μM/50 mg FW, indicating no statistically significant difference between them (**Figure 3B**). In contrast, under salinity stress, the proline concentration was significantly lower than the control and drought, approximately 130 μM/50 mg FW, indicating a statistically significant difference compared to both the control and drought conditions. However, the Manzanillo olive cultivar’s proline concentration was approximately 180 μM/50 mg FW, which was significantly different compared to both the salinity and drought conditions (**Figure 3C**). Under salinity stress, the proline concentration was significantly lower than the control, approximately 100 μM/50 mg FW, indicating a statistically significant difference compared to both the control and drought conditions. Finally, the proline level in the Nabali olive cultivar was approximately 160 μM/50 mg FW. This value indicated a statistically significant difference compared to both the salinity and drought conditions (**Figure 3D**). Under salinity and drought stresses, the proline concentration dropped significantly to approximately 70 and 100 μM/50 mg FW, respectively. These values indicated a statistically significant difference compared to the control condition (**Figure 3D**).

#### 3.1.3 Photosynthetic efficiency

**Figure 4:**
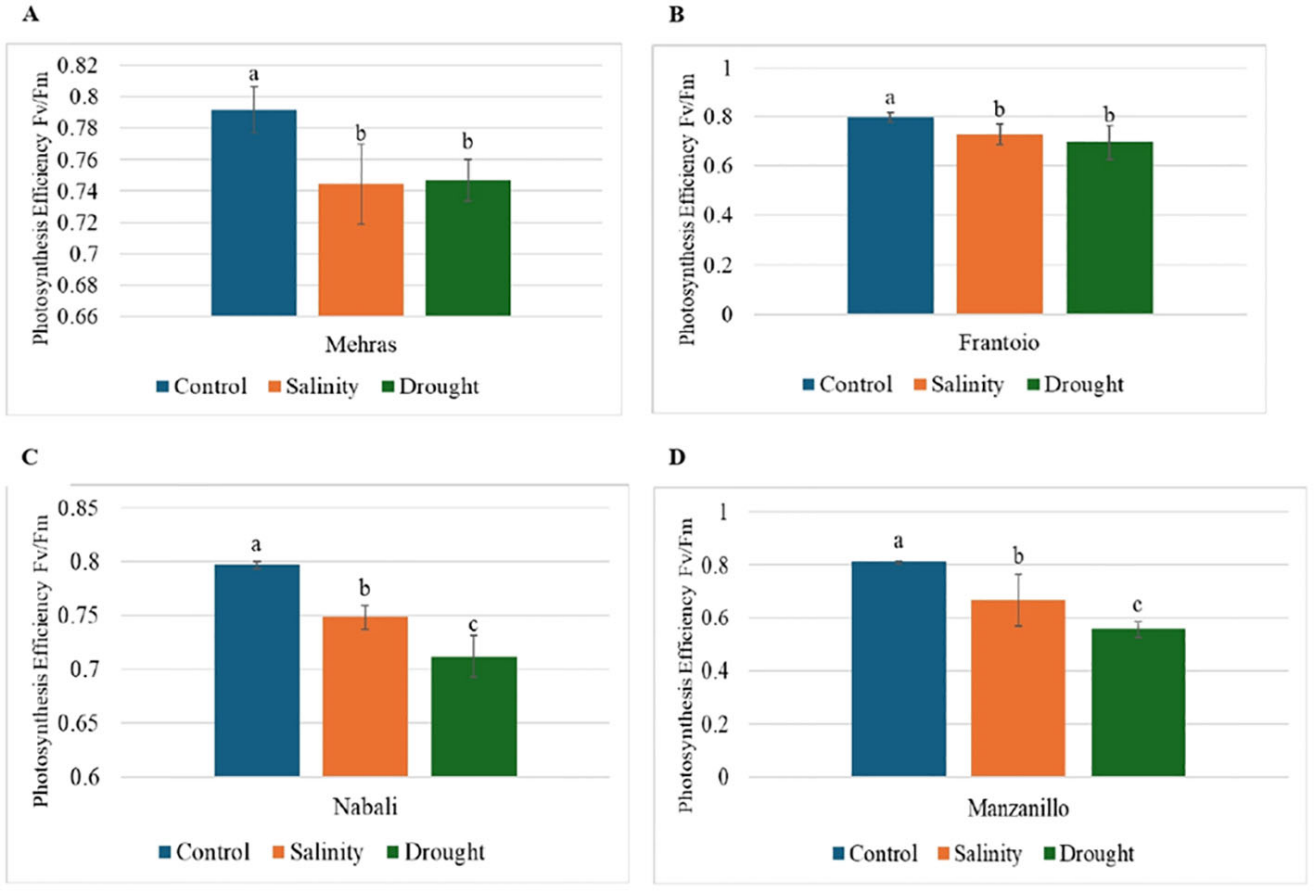
Analysis of photosynthesis efficiency (Fv/Fm) in olive cultivars under different stress conditions. Columns with the same letter are significantly not different using LSD. Error bars represent SD. **(A)** Mehras, **(B)** Frantoio, **(C)** Nabali and **(D)** Manzanillo olive cultivars.

The Fv/Fm ratio is a key parameter in chlorophyll fluorescence analysis and represents the maximum quantum efficiency of photosystem II (PSII) when the photosynthetic apparatus is without stress. Under optimal conditions, a healthy plant typically has an Fv/Fm ratio of approximately 0.75 (Edziri et al., 2021). Lower values indicate stress or damage to the photosynthetic apparatus, often due to environmental factors such as drought, temperature extremes, or nutrient deficiencies (Rico et al., 2023). The bar chart in **Figure 4** depicts the photosynthesis efficiency (Fv/Fm) of the four olive cultivars. **Figure 4A** shows the photosynthesis efficiency of the Mehras olive cultivar under three conditions. Under control conditions, the Mehras cultivar showed a high photosynthesis efficiency of approximately 0.79, which indicated optimal photosynthetic performance without any stress (**Figure 4A**). In contrast, under salinity and drought conditions, the Mehras cultivar exhibited lower photosynthesis efficiency values of approximately 0.74 and 0.75, respectively. However, decreasing Fv/Fm values under salinity and drought conditions were found in the Frantoio olive cultivar (**Figure 4B**). The photosynthesis efficiency under salinity stress was slightly higher than under drought stress, although not significantly. Similar trends were also achieved for both Nabali (**Figure 4C**) and Manzanillo (**Figure 4D**), however, there were significant differences between the salinity and drought-stressed plants.

### 3.2 Molecular analysis

**Figure 5:**
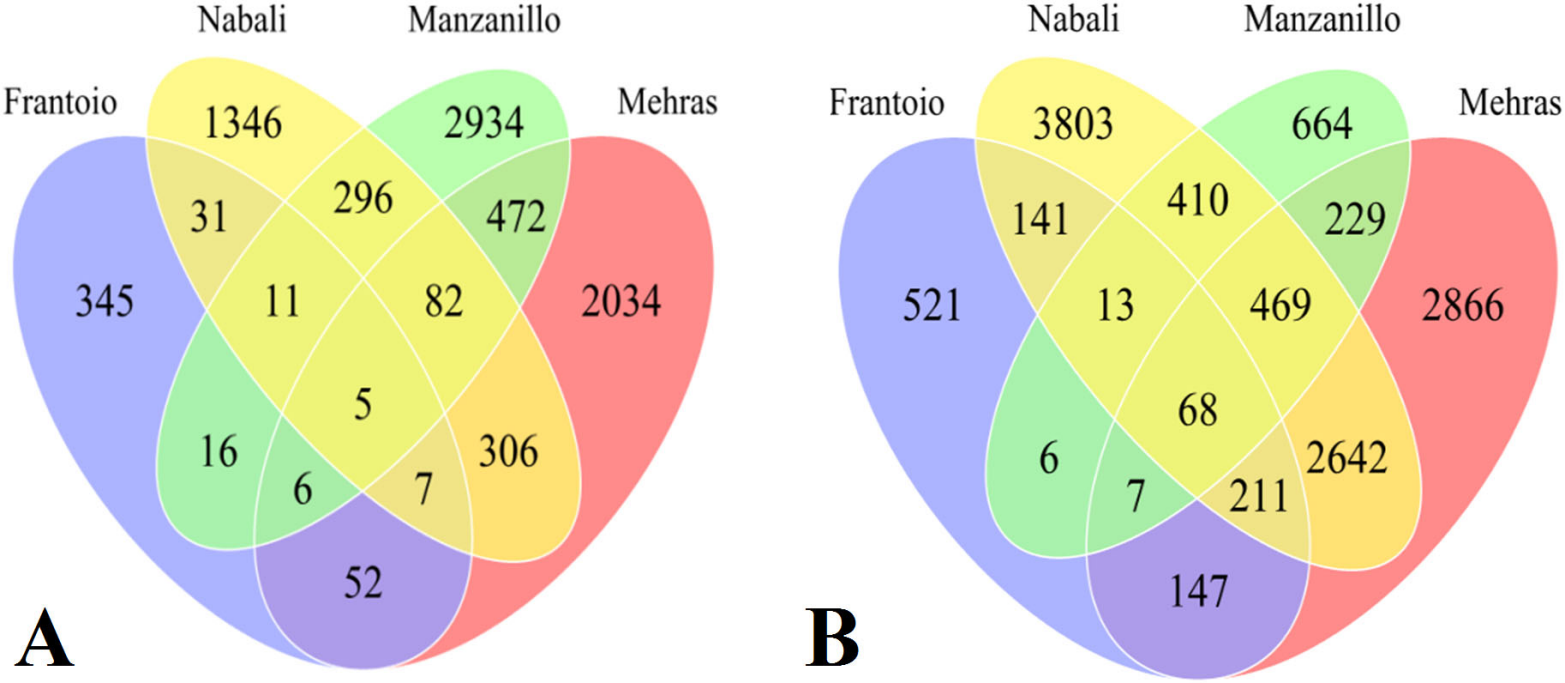
Venn diagrams of DEGs with 2-fold expression and above for olive cultivars ‘Frantoio’, Nabali’, Manzanillo’, and ‘Mehras’ under drought **(A)** or under salinity **(B)** as compared to the control.

This section examines the DEGs between the two abiotic stress treatments (salinity and drought) applied to four olive cultivars as compared to the control. Each run generated approximately 60 million reads of 100 bp. To assess the DEGs for each cultivar under stress treatments from the NGS-generated transcriptome data, RPKM data were generated for each composite biological sample. The RPKM data were log2 transformed to manage the huge discrepancies between the DEGs. Furthermore, the comparison of the four olive cultivars under salinity or drought stress treatment was performed using Venn diagrams for 2-fold expressions and above. The data revealed interesting findings with novel insights into the abiotic stress mitigation mechanism. The first group of comparisons included all four olive cultivars under drought stress, where Frantoio had 345 unique DEGs, while Nabali had 1,346 drought-specific DEGs, Manzanillo had 2,934 unique DEGs, and Mehras had 2,034 stress-specific DEGs (**Figure 5A**). Moreover, Nabali shared 296 DEGs with Manzanillo and 306 DEGs with Mehras, while the other two cultivars shared 472 DEGs. However, common DEGs were limited between the olive cultivars Frantoio and Nabali, Manzanillo, and Mehras, with only 31, 16, and 52 DEGs, respectively. The second group of comparison included all four olive cultivars under salinity stress, where Frantoio had 521 unique DEGs, while Nabali had 3,803 salinity-specific DEGs, Manzanillo had 664 unique DEGs, and Mehras had 2,866 stress-specific DEGs (**Figure 5B**). Moreover, Nabali shared 410 DEGs with Manzanillo and 2,642 DEGs with Mehras, while the other two cultivars shared 229 DEGs. Furthermore, common DEGs were also limited under salinity between the olive cultivars Frantoio and Nabali, Manzanillo and Mehras, with only 141, 6, and 147 DEGs, respectively.

**Figure 6:**
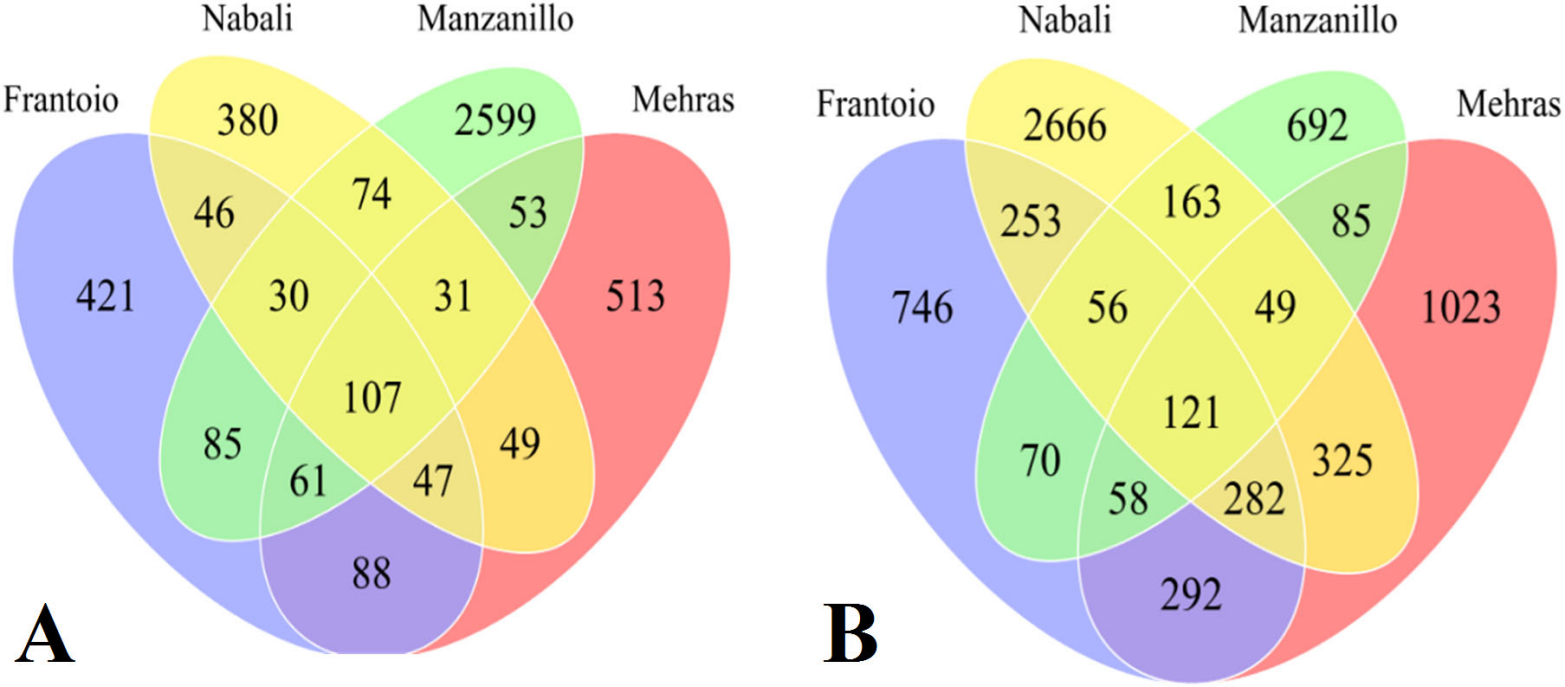
Venn diagrams of DEGs with 2-fold expression and above for olive cultivars ‘Frantoio’, Nabali’, Manzanillo’, and ‘Mehras’ under drought stress relative to salinity stress **(A)** or under salinity stress relative to drought stress **(B)**.

The third group of comparison included all four olive cultivars under drought relative to salinity, where Frantoio had 421 unique DEGs, while Nabali had 380 drought-specific DEGs, Manzanillo had 2,599 unique DEGs, and Mehras had 513 stress-specific DEGs (**Figure 6A**). Moreover, Nabali shared 74 DEGs with Manzanillo and 49 DEGs with Mehras, while the other two cultivars shared 53 DEGs. Furthermore, common DEGs were in comparable range between the olive cultivars Frantoio and Nabali, Manzanillo and Mehras, with 46, 85, and 88 common DEGs, respectively. The fourth group of comparison included all four olive cultivars under salinity stress relative to drought, where Frantoio had 746 unique DEGs, while Nabali had 2,666 salinity-specific DEGs, Manzanillo had 692 unique DEGs, and Mehras had 1,023 stress-specific DEGs (**Figure 6B**). Moreover, Nabali shared 163 DEGs with Manzanillo and 325 DEGs with Mehras, while the other two cultivars shared 85 DEGs. Moreover, common DEGs were in comparable range between the olive cultivars Frantoio and Nabali, Manzanillo and Mehras, with 253, 70, and 292 common DEGs, respectively.

**Figure 7:**
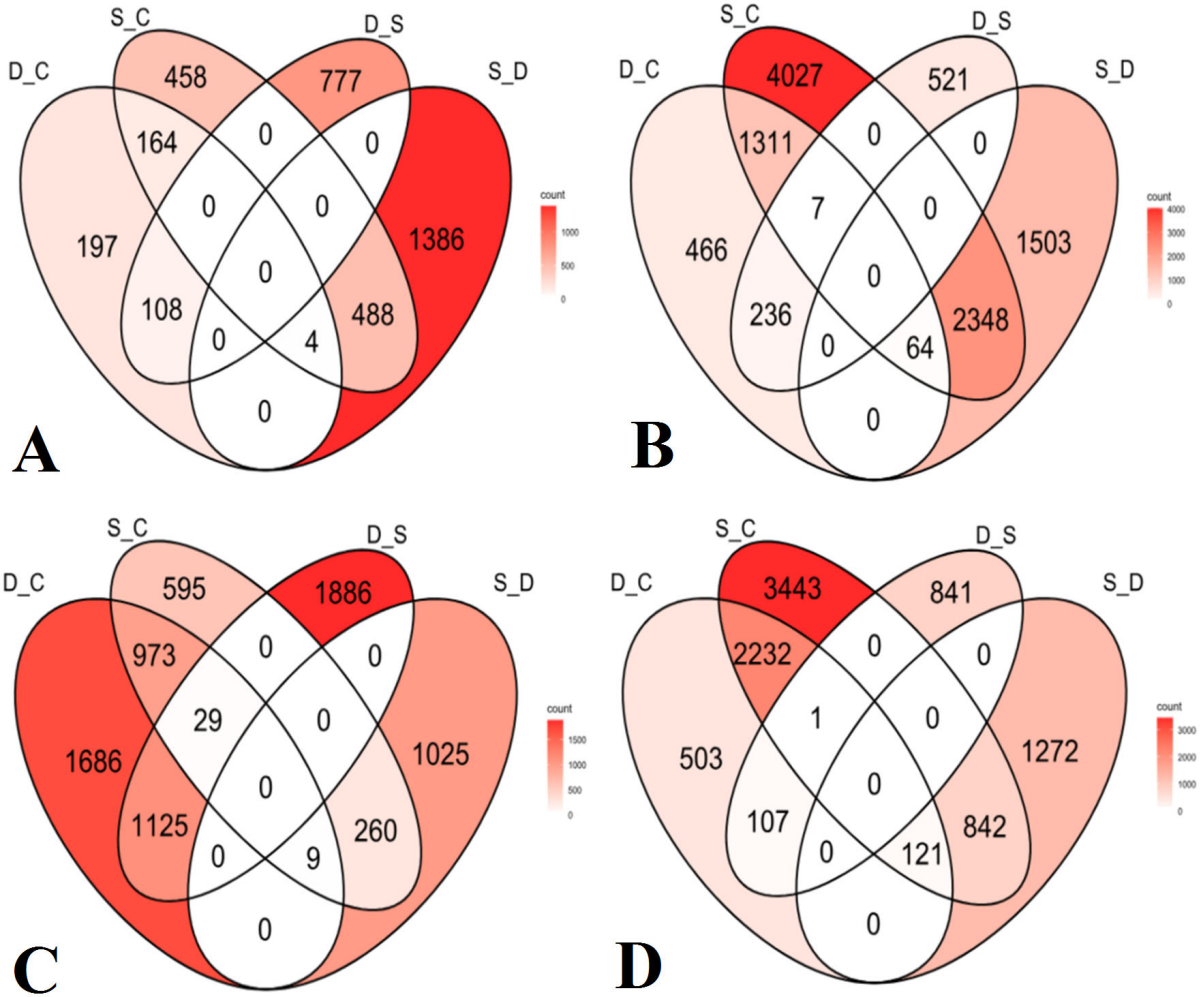
Venn diagrams of DEGs with 2-fold expression and above under drought stress relative to the control (D_C), salinity stress relative to the control (S_C), drought stress relative to salinity stress (D_S), and salinity stress relative to drought stress (S_D). Olive cultivars: **(A)** Frantoio, **(B)** Nabali, **(C)** Manzanillo, and **(D)** Mehras.

The fifth group of comparison included DEGs under drought stress relative to the control (D_C), salinity stress relative to the control (S_C), drought stress relative to salinity stress (D_S), and salinity stress relative to drought stress (S_D). These were calculated for each of the four olive cultivars. For Frantoio, D_C revealed 197 unique DEGs, while S_C showed 458 specific DEGs, D_S revealed 777 unique DEGs, and S_D showed 1386 stress-specific DEGs (**Figure 7A**). For Nabali, D_C revealed 466 unique DEGs, while S_C showed 4,027 specific DEGs, D_S revealed 521 unique DEGs, and S_D revealed 1,503 stress-specific DEGs (**Figure 7B**). For Manzanillo, D_C revealed 1,686 unique DEGs, while S_C showed 595 specific DEGs, D_S revealed 1,886 unique DEGs, and S_D revealed 1025 stress-specific DEGs (**Figure 7C**). Finally, for Mehras, D_C revealed 503 unique DEGs, while S_C showed 3443 specific DEGs, D_S revealed 841 unique DEGs, and S_D revealed 1,272 stress-specific DEGs (**Figure 7D**).

**Figure 8:**
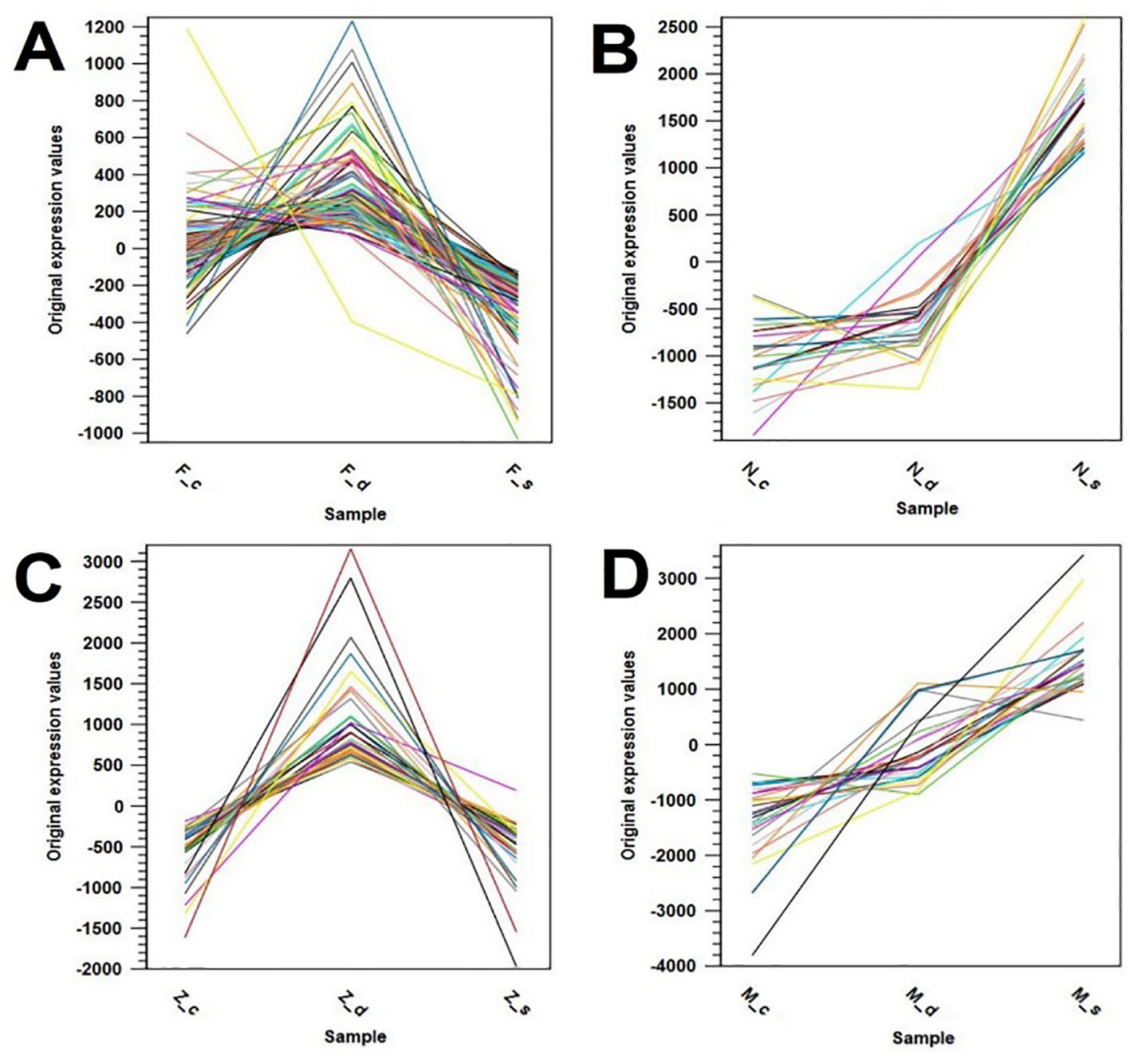
Gene expression pattern clusters showing similar expression patterns in the olive cultivars: **(A)** Frantoio (_F), **(B)** Nabali (_N), **(C)** Manzanillo (_Z), and **(D)** Mehras (_M), where _c is control, _d is drought stress, and _s is salinity stress.

Several gene expression pattern clusters were investigated for common gene expression patterns among the four olive cultivars. Four major clusters were found, one for each cultivar. The first cluster for olive cultivar Frantoio revealed upregulated gene expression under drought stress as compared to the control, which, in turn, under the salinity condition, dropped below the control level (**Figure 8**).

The second cluster for the olive cultivar Nabali showed elevated gene expression under salinity stress as compared to similarly low levels in both control and drought stress conditions (**Figure 8B**). The third cluster for olive cultivar Manzanillo showed upregulated gene expression under drought stress as compared to low levels under both control and the salinity conditions (**Figure 8C**). The final cluster for olive cultivar Mehras showed elevated gene expression levels under the drought condition as compared to the control, which in turn were far more elevated under the salinity condition than the drought stress levels (**Figure 8D**). The top 100 upregulated DEGs with a 2-fold expression and above cut-off point were listed for all four olive cultivars under both abiotic stress treatments as compared to the control. The top 100 DEGs with 2-fold expression and above in Frantoio under drought stress relative to the control (**Supplementary Tables 2**-**Supplementary Tables 9**) showed one upregulated DEG with more than 5-fold expression and a large group of DEGs above 3-fold expression. This includes ATP-dependent 6-phosphofructokinase, the WRKY family transcription factor family protein, and the ABC transporter family protein. The top five DEGs are listed in **Table 1**.

**Table 1:** Top 5 upregulated DEGs in olive cultivars Frantoio (F), Nabali (N), Manzanillo (Z), and Mehras (M) under d/c (drought stress relative to control) and s/c (salinity stress with relative to control) with fold change values.

#	Feature ID	Description	F_d/c	Feature ID	Description	F_s/c
1	Oeu032705.1	Lemir	5.5	Oeu016087.1		5.9
2	Oeu025312.1	Phytosulfokines 3	4.9	Oeu033675.1	GA 20-oxidase family	5.7
3	Oeu056985.2		4.8	Oeu002507.1	beta-galactosidase	5.5
4	Oeu018758.1	Kunitz-type protease inhibitor KPI-	4.4	Oeu037614.1		5.5
5	Oeu007004.1	tRNA synthetase class i	4.1	Oeu007811.1	DUF1005 family protein	5.5
#	Feature ID	Description	N_d/c	Feature ID	Description	N_s/c
1	Oeu032705.1	Lemir	8.2	Oeu012604.1	Xyloglucan endotransglucosylase hydrolase	7.0
2	Oeu035127.1	Lipid transfer protein	7.6	Oeu035401.1	At1g36320 f7f23_4	6.7
3	Oeu050850.1		7.6	Oeu024369.3		6.6
4	Oeu047443.1	At4g32480 f8b4_180	6.0	Oeu060006.1	NDH-dependent cyclic electron flow	6.5
5	Oeu001768.1	UDP-glucoronosyl UDP-glucosyl transferase	5.8	Oeu017977.1	GDSL-motif lipase hydrolase	6.4
#	Feature ID	Description	Z_d/c	Feature ID	Description	Z_s/c
1	Oeu029201.1	Non-specific lipid-transfer	8.8	Oeu012144.1	Auxin repressed dormancy	6.8
2	Oeu015612.1	Allene oxide synthase	6.5	Oeu038414.1		6.6
3	Oeu017720.1	U-box domain-containing	6.4	Oeu007780.1	heat shock protein	6.5
4	Oeu037159.1	AVR9 CF-9 rapidly elicited	6.4	Oeu059302.1		5.8
5	Oeu001701.1	Glycosyl transferase 17	6.3	Oeu029201.1	Non-specific lipid-transfer	5.3
#	Feature ID	Description	M_d/c	Feature ID	Description	M_s/c
1	Oeu043352.1	ATP-dependent CLP protease proteolytic subunit	5.2	Oeu052645.1	Ankyrin repeat	7.0
2	Oeu021363.2	Zinc finger family protein	5.0	Oeu040651.1	Xyloglucan endotransglucosylase hydrolase	6.9
3	Oeu032705.1	Lemir	5.0	Oeu044952.2	Peptide nitrate transporter	6.7
4	Oeu003335.2	wd g-beta repeat protein	5.0	Oeu024323.1	MYB 109	6.6
5	Oeu035711.1	Basic helix-loop-helix	4.9	Oeu057521.1	Iron transporter-related	6.6

Our aim was to find major novel biomarkers in the investigated historic olive cultivars as major genetic resources in olives to mitigate salinity and drought in the future. These are the top up-regulated genes listed in **Table 1** in the four cultivars, but mainly for drought tolerance in Frantoio and Manzanillo and for salinity tolerance in the Nabali and Mehras cultivars.

#### 3.2.1 Differentially expressed genes

DEGs are genes that show significant changes in expression level between different experimental conditions, such as stress versus control conditions. These changes in expression are typically analyzed using RNA sequencing (RNA-seq) or microarray data, and they provide insights into the biological responses of organisms. The fold change (FC) is a common metric used to quantify the level of gene expression differences, representing the ratio of expression levels under different conditions. A positive fold change indicates upregulation, while a negative fold change suggests downregulation. The identification and analysis of DEGs and their fold changes are crucial for understanding molecular mechanisms, pathways, and key regulatory genes involved in specific biological processes or stress responses.

The top 100 upregulated DEGs above the 2-fold expression and above cut-off point were listed for all four olive cultivars under both abiotic stress treatments as compared to the control. The top 100 DEGs with 2-fold expression and above in Frantoio under drought stress relative to the control (**Supplementary Table 2**) showed one upregulated DEG with more than 5-fold expression and a large group of DEGs above 3-fold expression. This included ATP-dependent 6-phosphofructokinase, the WRKY family transcription factor family protein, and the ABC transporter family protein.

Furthermore, the top 100 DEGs with 2-fold expression and above in Frantoio under salinity stress relative to the control (**Supplementary Table 3**) showed 10 upregulated DEGs with more than 5-fold expression and a large group of DEGs with above 4-fold expression. This included gibberellin 20-oxidase family protein, beta-galactosidase, duf1005 family protein, and eukaryotic aspartyl protease family protein.

The top 100 DEGs with 2-fold expression and above in Nabali under drought stress relative to the control (**Supplementary Table 4**) showed one upregulated DEG with more than 8-fold expression and a large group of DEGs above 4-fold expression. This included potassium efflux antiporter, SWEET sugar transporter, ankyrin repeat family protein, and c4-dicarboxylate transporter malic acid protein. Furthermore, the top 100 DEGs with 2-fold expression and above in Nabali under salinity stress relative to the control (**Supplementary Table 5**) showed 10 upregulated DEGs with more than 5-fold expression and a large group of DEGs with above 4-fold expression. This included GDSL-motif lipase hydrolase family protein, 3-ketoacyl-synthase, mitochondrial transcription termination factor family protein, and gland-specific fatty acyl-reductase.

The top 100 DEGs with 2-fold expression and above in Manzanillo under drought stress relative to the control (**Supplementary Table 6**) showed one upregulated DEG with more than 8-fold expression and a large group of DEGs with above 5-fold expression. This included heavy metal transport detoxification superfamily protein, RAP2-like protein, MOL-like protein, mitogen-activated protein kinase, alcohol dihydrogen family protein, and AP2 ERF domain transcription factor. Furthermore, the top 100 DEGs with 2-fold expression and above in Manzanillo under salinity stress relative to the control (**Supplementary Table 7**) showed three upregulated DEGs with more than 6-fold expression and a large group of DEGs with above 4-fold expression. This included auxin-repressed dormancy-associated protein, heat shock protein, non-specific lipid-transfer protein, and universal stress family protein.

The top 100 DEGs with 2-fold expression and above in Mehras under drought stress relative to the control (**Supplementary Table 8**) showed four upregulated DEGs with more than 5-fold expression and a large group of DEGs with above 4-fold expression. This included ATP-dependent clp protease proteolytic subunit, zinc finger family protein, T-box family protein, TCP family transcription factor, diacylglycerol kinase, alpha-mannosidase, and hexosyltransferase.

Furthermore, the top 100 DEGs with 2-fold expression and above in Mehras under salinity stress relative to the control (**Supplementary Table 9**) showed one upregulated DEG with more than 7-fold expression and a large group of DEGs with above 5-foldm expression. This included xyloglucan endotransglucosylase hydrolase, peptide nitrate transporter plant, MYB transcription factor myb109, iron transporter-related family protein, receptor-like protein kinase Feronia, F-box family protein, and xyloglucan endotransglucosylase hydrolase.

The expression patterns of stress responsive genes were investigated with qPCR for a group of olive genes. The fold increase in expression level was compared to that revealed by the olive RNA-Seq, which showed comparable trends in expression.

## 4 Discussion

The major aim of this research was to study these historic olive cultivars and find unique responsive genes that could be used as biomarkers to mitigate abiotic stresses from climate change. A physiology analysis is a vital indicator of any stress. If the exposed stress level does not or marginally affects the plant physiology, then this should be considered the normal condition for the plant in question. However, if the measured physiological or morphological parameters indicate a significant reduction as compared to the control plants, then this would confirm the onset of the stress, which is important when searching for major DEGs related to the mitigation of specific abiotic stress.

### 4.1 Physiological responses of historic olives to abiotic stresses

Under control conditions, all four cultivars exhibited high RWC values, ranging from 80%–90%, reflecting optimal hydration levels and the absence of water stress. Specifically, Mehras, Frantoio, Manzanillo, and Nabali maintained RWC values of 87.7764%, 82.7374%, 81.2385%, and 80.3249%, respectively. These high values indicated the cultivars’ capacity to maintain adequate leaf hydration under favorable conditions, which is crucial for sustaining physiological processes, including photosynthesis.

Leaf RWC was lower in the salinity and drought stress conditions compared to the control. The external solution with high salt concentration led to osmotic stress and dehydration at the cellular level, which caused the drop in RWC. Therios and Misopolinos (1988) observed that salinized olive trees absorbed less water, mostly due to the lower osmotic potential of NaCl-containing solutions. Gucci et al. (1997) presented a thorough examination of the water relation features of olive leaves under salt stress for the salt-tolerant Frantoio and salt-sensitive Leccino cultivars. Similar to most woody crops, olives quickly reduce their RWC and leaf water potential (Cw) in response to salinity. Moreover, higher salinity levels generated similar changes in other fruit tree species, causing alterations in Cw, RWC, and water uptake (Banuls and Primo-Millo, 1992). According to Hassan et al. (2020), electrolyte leakage increased as salinity concentrations increased from 0 to 4,000 mg L^-1^. In fact, salinity stress steadily lowered the relative water content of the leaves in all cultivars when compared to the control. There were notable variations in leaf water content across the cultivars under investigation. The Aggizi, Shami cultivars displayed the lowest relative water content (76.25%), whilst the Picual cultivar displayed the highest relative water content (83%). Manzanillo’s relative water content was found to be in the middle range at 78.25%. Therefore, the amount of electrolyte leakage differed among olive cultivars. Additionally, the researchers observed that all three cultivars, namely, the Picual, Manzanillo, and Aggizi, Shami cultivars, that were exposed to salt stress showed a lower decrease in vegetative growth parameters (seedling height, number of leaves, and leaf area) and total chlorophyll content, and greater increase in proline, soluble carbohydrates, and electrolyte leakage (Hassan et al., 2020).

This study examined the proline levels in the leaves of four olive cultivars (Mehras, Frantoio, Manzanillo, and Nabali) under control, salinity stress, and drought stress conditions. Proline content is known to support homeostasis during salt stress through osmotic control (Di Martino et al., 2003; Diez et al., 2015). In this experiment, the proline levels were highest under the control conditions and decreased under salinity and drought stress. This agrees with what was found by Regni et al. (2019), where the content of proline varied across four olive cultivars in non-stress conditions; it was greater in Royal and Koroneiki and less noticeable in Fadak 86 and Arbequina. In our study, proline decreased in the saline-stressed plants of all four cultivars in a statistically significant way, which is in agreement with earlier observations (Ayala-Astorga and Alcaraz-Meléndez, 2010). In contrast, Vita et al. (2022) found that proline in olive leaves was not affected by salt stress, apart from a significant increase in a single cultivar, Oliana, under 200 mM salt stress (Vita et al., 2022). Regni et al. (2019) investigated the behavior of four olive cultivars under salt stress and found that proline levels generally decreased in response to salinity, similar to the observed trend in Mehras. According to Karimi et al. (2018), the leaves of the Conservolia olive cultivar showed the highest proline concentration (157.8 μmol g^-1^) during the drought, whereas the leaves of Fishomi had the lowest value (116.8 μmol g^−1^) (Karimi et al., 2018). The results showed that Mehras had a proline level of 160.32 µmol/g FW under control conditions, which decreased to 144.53 µmol/g FW under salinity stress and further to 131.02 µmol/g FW under drought stress. Similarly, Nabali had a proline level of 162.4 µmol/g FW under control conditions, decreasing to 79.61 µmol/g FW under salinity stress and 102.84 µmol/g FW under drought stress. Manzanillo showed a drastic decrease in proline level from 178.76 µmol/g FW under control conditions to 61.87 µmol/g FW under salinity stress and 121.39 µmol/g FW under drought stress. In contrast, Frantoio exhibited resilience with proline levels decreasing slightly from 186.91 µmol/g FW under control conditions to 177.06 µmol/g FW under salinity stress and increasing to 188.4 µmol/g FW under drought stress. These findings align with previous research on the physiological responses of olive cultivars to environmental stress. For instance, a study by Gholami and Zahedi (2019) found that certain olive genotypes showed superior drought tolerance through biochemical adaptations, including proline accumulation, which supports the current study’s findings on Frantoio resilience under drought conditions. Similarly, Regni et al. (2019) reported that different olive cultivars, such as Arbequina and Koroneiki, exhibited varied responses to salinity stress, with significant decreases in proline levels and other physiological changes, which parallels the observed responses in Mehras and Nabali under salinity stress (Regni et al., 2019).

The study investigated the photosynthesis efficiency (Fv/Fm) in the leaves of four olive cultivars (Mehras, Frantoio, Manzanillo, and Nabali) under control, salinity, and drought stress conditions. The results demonstrated a decrease in photosynthesis efficiency under both stress conditions for all the cultivars, with the most significant reductions observed under drought stress. For instance, Manzanillo exhibited the most substantial decline, with efficiency dropping from 0.8106 under the control to 0.667 under salinity stress and 0.5587 under drought stress. This pattern is in agreement with the findings of ​ El Yaman and Cordovilla (2024), who highlighted that salinity and drought stress induce osmotic and ionic toxicity, impacting chlorophyll function and thus reducing photosynthesis efficiency in olive trees​. Gholami and Zahedi (2019) also documented significant reductions in photosynthesis efficiency in drought-stressed olive genotypes. Their research highlighted that drought conditions induce stomatal closure, which reduces CO_2_ availability, and oxidative stress, which damages the photosynthetic apparatus. This aligns with the current study’s observation of lower photosynthesis efficiency in drought-stressed cultivars, particularly in Manzanillo and Frantoio, suggesting that these cultivars are more susceptible to drought-induced stress. The variability in stress response among the cultivars can be partly explained by the differential activity of antioxidant enzymes, as observed by Regni et al. (2019). Their study showed that cultivars with higher activities of antioxidant enzymes, such as glutathione (GSH) and catalase (CAT), were better able to maintain photosynthetic performance under salinity stress. This is consistent with the relatively better performance of Nabali under both stress conditions, which may be due to more effective oxidative stress management. However, the significant drop in photosynthesis efficiency in Manzanillo indicates a lower capacity to counteract the oxidative damage induced by drought stress (Regni et al., 2019). Additionally, Kchaou et al. (2010) assessed the tolerance of different olive cultivars to NaCl salinity and found that efficient ion regulation mechanisms and robust antioxidant defenses were crucial for maintaining photosynthetic efficiency under stress. The findings of the present study support this, as the cultivars that showed a lesser reduction in photosynthesis efficiency under stress conditions, such as Mehras and Nabali, are likely to have better ion regulation and antioxidant responses. These results underscore the importance of selecting and breeding olive cultivars with enhanced physiological and biochemical stress tolerance mechanisms to ensure sustainable productivity in environments prone to salinity and drought (Kchaou et al., 2010).

### 4.2 Olive DEGs under drought and salinity

#### 4.2.1 Drought stress

The current investigation revealed both unique and common DEGs between the investigated olive cultivars Frantoio, Nabali, Manzanillo, and Mehras under drought stress. The striking finding is related to the absolute number of unique DEGs compared to the common ones. The reason both Manzanillo and Mehras showed the highest numbers of unique DEGs, 2,934 and 2,034, respectively, could be linked to their long adaptation history. This was proved for Mehras by studying its phylogenetic position among major Mediterranean olive cultivars based on both the plastome (Haddad et al., 2021) and the mitogenome (Sadder et al., 2023a). In contrast, Nabali showed an intermediate number of unique DEGs under drought stress (1346 DEGs), while Frantoio had the lowest potential to withstand drought stress, as it showed only 345 unique DEGs. Our data are novel for olives under drought, as limited studies of gene expression have been conducted in olives under drought stress (Nteve et al., 2024). The Freila olive cultivar under drought stress showed upregulation of genes related to transcription factors induced by ABA, auxin, and ethylene signaling, and the action of a predicted membrane intrinsic protein (MIP) (Calvo-Polanco et al., 2019). In contrast, the responses of Grazalema trees with enough water supply were related to different root genes related to oxidation-reduction, ATP synthesis, transduction, and posttranslational regulation, with special mention of cytokinin signaling through the transcript predicted to be a histidine-containing phosphotransferase protein (Calvo-Polanco et al., 2019).

Although there is extensive literature on drought stress, the level of coverage for olive crops is quite limited. An RNA-seq meta-analysis conducted by Benny et al. (2020) examined five fruit tree crops from six published studies, including olive trees, under drought and salinity stress. In total, 26 RNA-seq samples were analyzed, and 683 genes were identified as commonly regulated among the three drought studies. A comparison was also employed of the genes that were common among both salinity and drought, resulting in 82 genes, of which 39 were regulated with the same trend of expression (Benny et al., 2020). Later, a study was published regarding olive trees (cv. Souri) and the effect of water stress on non-structural carbohydrates (NSC) and starch regulation, suggesting a group of stress-related starch metabolism genes, correlated with NSC fluctuations during drought and recovery (Tsamir-Rimon et al., 2021). In the published Olive Atlas, containing 70 RNA-seq experiments (Bullones et al., 2023), the experiments related to drought were based on the Souri variety and included a total of three experiments. The Souri drought experiments were conducted using RNA-seq technology, and the data were analyzed using the Picual genome sequence and gene annotation as a reference. To obtain detailed information about the genes identified in the framework of the Olive Atlas, a public platform was released in 2023 (https://www.oliveatlas.uma.es/). These findings indicate that plants, upon detecting changes in environmental conditions, may activate distinct genetic pathways to initiate adaptive responses (Bullones et al., 2023).

**Figure 9:**
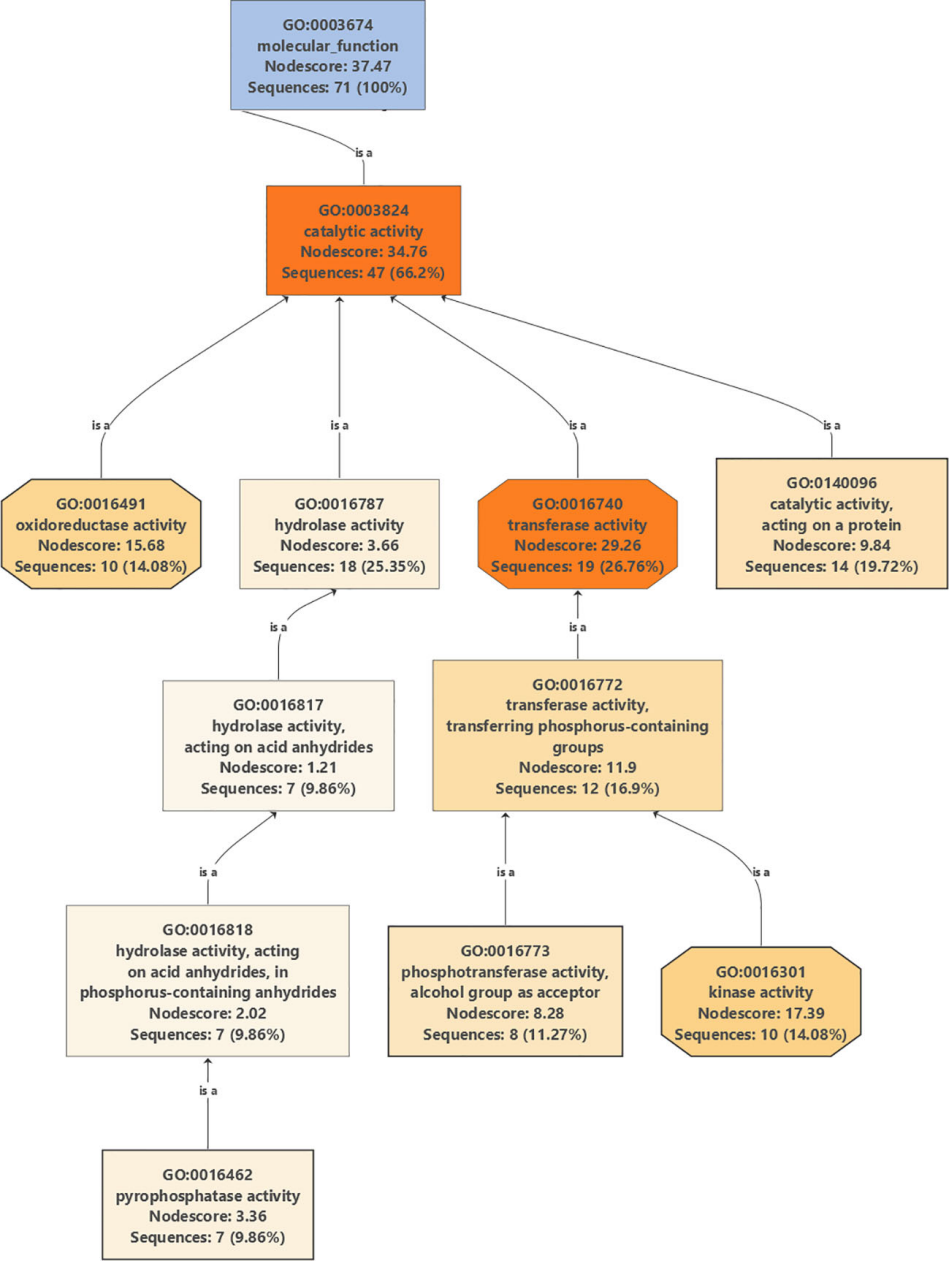
Partial Gene Ontology graph (molecular function) for DEGs in Mehras under drought stress compared to control conditions.

Gene Ontology graphs were generated for top DEGs in all cultivars under drought stress for biological processes (**Supplementary Figures 2**, **Supplementary Figures 8**, **Supplementary Figures 14**, and **Supplementary Figures 20**), cellular localizations (**Supplementary Figures 3**, **Supplementary Figures 9**, **Supplementary Figures 15**, and **Supplementary Figures 21**), and molecular functions (**Supplementary Figures 4**, **Supplementary Figures 10**, **Supplementary Figures 16**, and **Supplementary Figures 22**). A careful examination of a major part of the molecular functions graph for the cultivar Mehras (**Figure 9**), which was found to be the most active olive cultivar in unique DEGs under salinity (**Figure 7D**), showed that a major part of mitigation was linked to signaling pathways.

#### 4.2.2 Salinity stress

Similar to drought stress, the current investigation revealed both unique and common DEGs between the investigated olive cultivars Frantoio, Nabali, Manzanillo, and Mehras under salinity stress. However, the data were different regarding the absolute number of unique DEGs compared to the common ones. Under salinity stress, Nabali and Mehras, rather than Manzanillo, had the highest number of unique DEGs, 3,803 and 2,866, respectively, which again could be related to their long adaptation history. They were followed by a relatively small number of unique DEGs for Frantoio and Manzanillo with 521 and 664 DEGs, respectively. When comparing drought stress with salinity stress, Manzanillo had the largest number of unique DEGs with 2,599, and when comparing salinity stress with drought stress, Nabali had the largest number of unique DEGs with 2,666 followed by 1,023 DEGs for Mehras. Furthermore, when making the comparisons within each cultivar (**Figure 4**), the Jordanian cultivars were skewed towards salinity stress with 4,027 and 3,443 unique DEGs for Nabali and Mehras, respectively, again indicating their historic adaptation (Haddad et al., 2021; Sadder et al., 2023). The olive cultivar Frantoio had a low number for both unique salinity and drought DEGs, with 458 and 197, respectively, indicating its limited adaptation to both abiotic stresses. When investigating the Manzanillo cultivar, it showed an intermediate number of unique drought-stress DEGs (1686) and a low number of unique salinity-stress DEGs (595). The overlap between drought and salinity stress DEGs showed a relatively good number, with 1,311 and 2,232 DEGs for Nabali and Mehras, respectively. Before the introduction of NGS, studies were limited to cDNA library construction and Sanger sequencing of ESTs and small-scale microarray analysis (around 1 k ESTs). In a study of salinity stress in olives (Sadder et al., 2021), the novel salinity-responsive biomarkers (SRBs) were monooxygenase1 (OeMO1), cation calcium exchanger1 (OeCCX1), salt tolerance protein (OeSTO, proteolipid membrane potential modulator (OePMP3), universal stress protein (OeUSP2), adaptor protein complex 4 medium mu4 subunit (OeAP-4), WRKY1 transcription factor (OeWRKY1), and potassium transporter 2 (OeKT2). Unique structural features were highlighted for encoded proteins compared with other plant homologs. The expression of olive SRBs was investigated in the leaves of young plantlets of two cultivars, Nabali (moderately tolerant) and Picual (tolerant). At the 60 mM NaCl stress level, OeMO1, OeSTO, OePMP3, OeUSP2, OeAP-4, and OeWRKY1 were upregulated in Nabali as compared with Picual. Furthermore, OeCCX1 and OeKT2 were upregulated at three stress levels (30, 60, and 90 mM NaCl) in Picual compared to Nabali. Despite the limited number of probe sets, transcriptional regulatory networks have been successfully constructed for two olive cultivars, Kalamon and Chondrolia, while several hierarchically clustered interacting transcription factor regulators, such as JERF and bZIP homologs, were identified (Bazakos et al., 2012). The first NGS study to identify DEGs in olives under salinity (Bazakos et al., 2015) utilized an old technology of 454 pyrosequencing, yielding a relatively low number of reads compared to the recent technology covered in this study. They found that in leaves, among the 2,642 clusters, 70 genes were identified as differentially expressed, with 14 down and 56 upregulated genes, while in the current study, thousands of DEGs were revealed. Later, a study was conducted with the Frantoio olive cultivar as the tolerant genotype compared to Leccino as the sensitive genotype under salinity stress (Rossi et al., 2016). The data showed differential transcript levels of five key genes of the phenylpropanoid pathway measured by quantitative real-time PCR. Our data, in contrast, classifies Frantoio as less salt-tolerant when compared to both Nabali and Mehras. Advanced NGS using RNAseq has been used to compare four olive cultivars, as we did in our study under salinity stress. Palm et al. (2024) compared the olive cultivars Frantoio, Leccino, Lecciana, and Oliana under salinity stress (Palm et al., 2024). They found that Frantoio showed the highest DEGs, revealed as proteins, followed by Leccino, both with a significant change in the proteome repertoire, with an overrepresentation of components regulating cellular metabolism, ion transport, redox insult, and dissipation of excess photochemical energy. In a similar study with different olive cultivars, namely Koroneiki, Picual, Royal de Cazorla, and Fadak86, Mousavi et al. (2021) found that Royal de Cazorla was the most tolerant cultivar, and Fadak86 and Picual were the most susceptible ones (Mousavi et al., 2021). They showed that *OeNHX7*, *OeP5CS*, *OeRD19A*, and *OePetD* were upregulated in tolerant cultivars.

**Figure 10:**
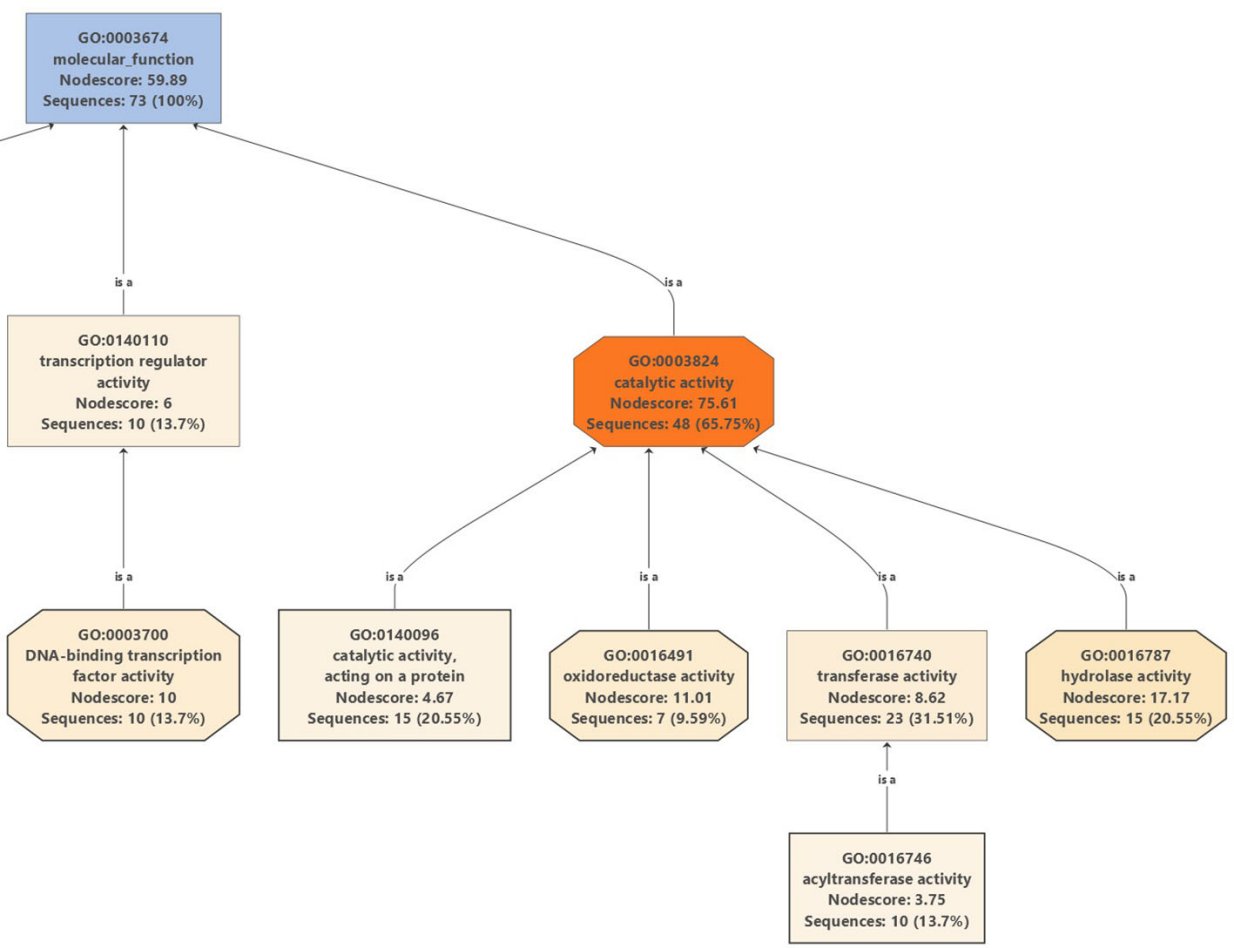
Partial GO graph (molecular function) for DEGs in Manzanillo under salinity stress compared to control conditions.

Moreover, Gene Ontology graphs were generated for the top DEGs in all cultivars under salinity stress for biological processes (**Supplementary Figures 5**, **Supplementary Figures 11**, **Supplementary Figures 17**, and **Supplementary Figures 23**), cellular localizations (**Supplementary Figures 6**, **Supplementary Figures 12**, **Supplementary Figures 18**, and **Supplementary Figures 24**), and molecular functions (**Supplementary Figures 7**, **Supplementary Figures 13**, **Supplementary Figures 19**, and **Supplementary Figures 25**). A careful examination of a major part of the molecular functions graph in the cultivar Manzanillo (**Figure 10**), which was found to be the most active olive cultivar in unique DEGs under salinity (**Figure 6C**), shows that a major part of mitigation was linked to ROS alleviation.

An additional major concern when studying drought and salinity stresses is the underground part of the plant, the roots. Although root studies are limited due to the complexity of the root system and its interactions with the surrounding substrate and soil microbiome, new innovative methods are being adapted and developed to study the root system under abiotic stresses (Kul et al., 2020; Karlova et al., 2021; Chen et al., 2025). Nonetheless, it is important to distinguish between root systems for perennials and annuals, where annual plant roots need to mitigate the stress in one season, but perennial plant roots are there for years in the soil and behave differently with potential long-term strategies. This makes it more difficult to investigate just the short-term effects of perennial plant roots rather than a complex extensive long-term study.

## 5 Conclusions

The investigated historic olive cultivars vary in their response to abiotic stresses, with limited common features. The historic Jordanian cultivars were found to be more salinity tolerant than their Mediterranean counterparts. However, all the cultivars revealed unique DEGs (biomarkers) under both salinity and drought stresses. This could indicate that each cultivar has followed a different adaptation route, leading to huge variations in genetic responses.

## Data Availability

The datasets presented in this study can be found in online repositories. The names of the repository/repositories and accession number(s) can be found in the article/**Supplementary Material**. Data was submitted as SRA at NCBi with BioProject PRJNA1267179 and BioSample accessions SAMN48713327-362.
