# Supplementary material for: Transcriptome analysis of historic olives reveals stress-specific biomarkers
Source: Front Plant Sci. 2025 Jun 5;16:1549305. doi: 10.3389/fpls.2025.1549305 (PMC12176842; doi:10.3389/fpls.2025.1549305)
Supplement: Supplementary file 1 [file DataSheet1.docx]

Supplementary Material

# Supplementary Data

Supplementary Material should be uploaded separately on submission. Please include any supplementary data, figures and/or tables.

Supplementary material is not typeset so please ensure that all information is clearly presented, the appropriate caption is included in the file and not in the manuscript, and that the style conforms to the rest of the article.

# Supplementary Figures and Tables

## Supplementary Figures

**
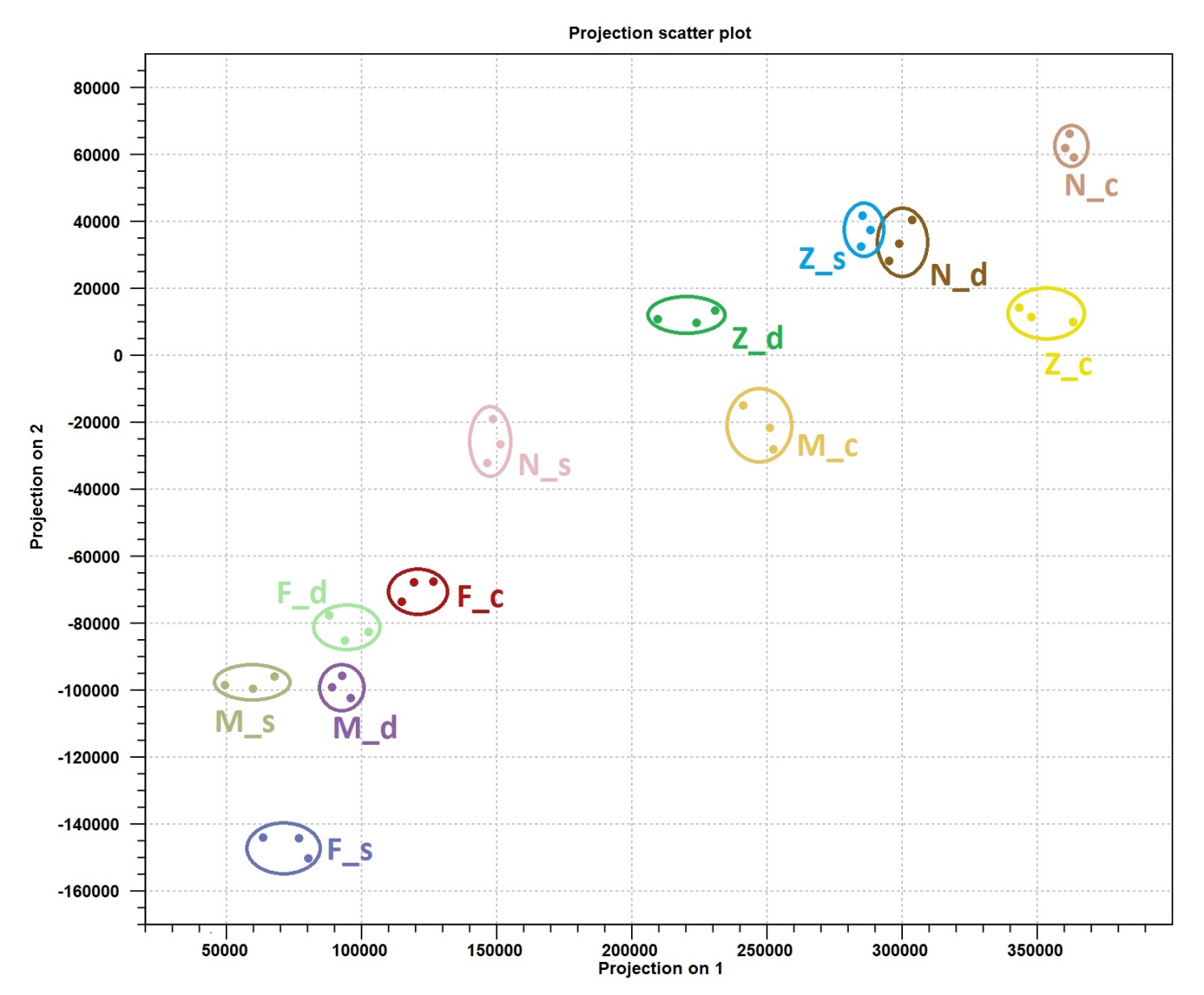
**

**Supplementary Figure 1**: PCA for RNA-seq data for all olive cultivars and treatments. ‘Mehras’ (M), ‘Nabali’ (N), ‘Manzanillo’ (Z) and ‘Frantoia’ (F). Treatments: control (_c), salinity (_s) and drought (_d).


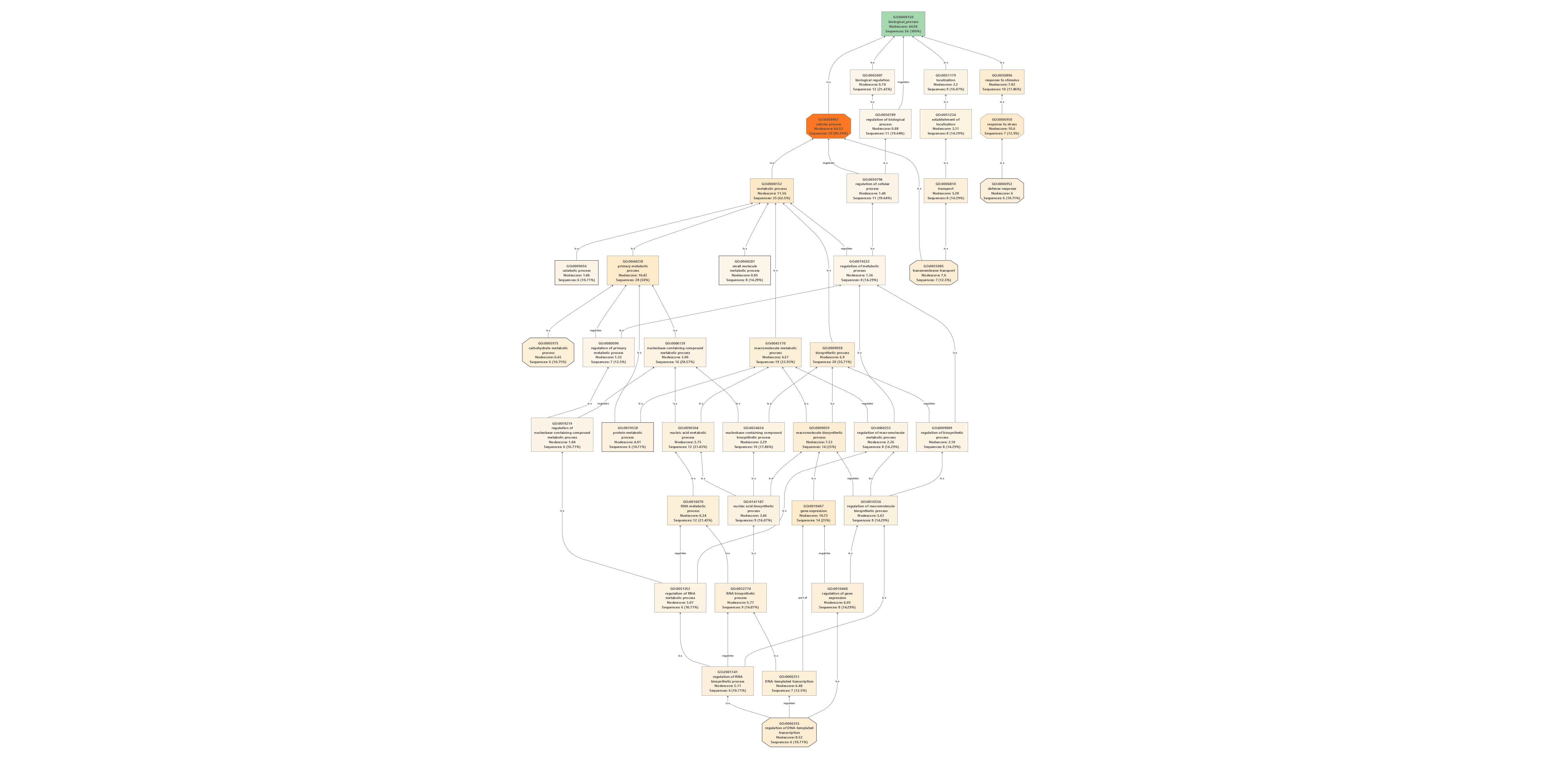


**Supplementary Figure 2**: GO graph (Biological processes) for DEGs in Frantoia under drought stress compared to control conditions.


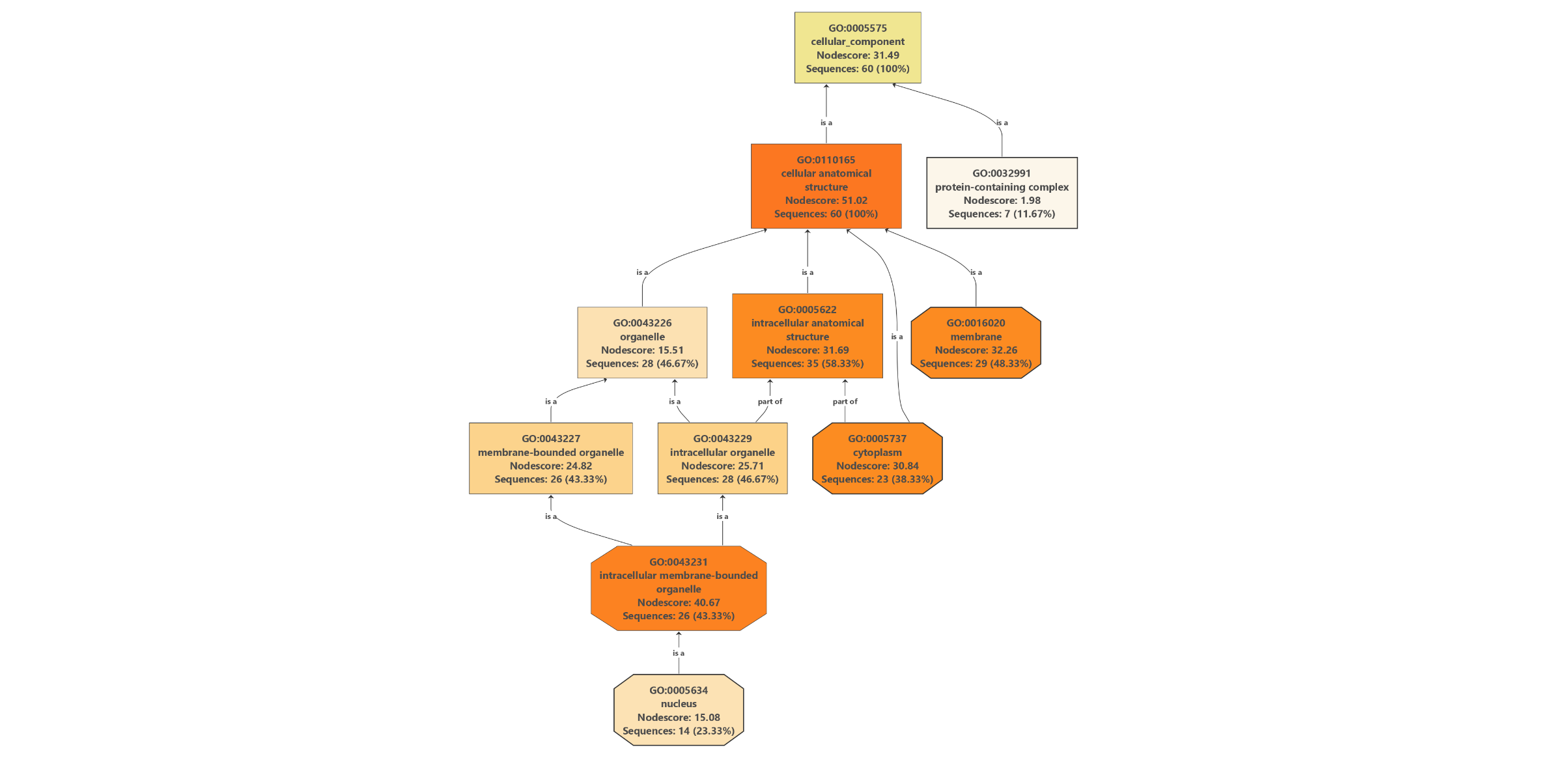


**Supplementary Figure 3**: GO graph (Cellular localization) for DEGs in Frantoia under drought stress compared to control conditions.


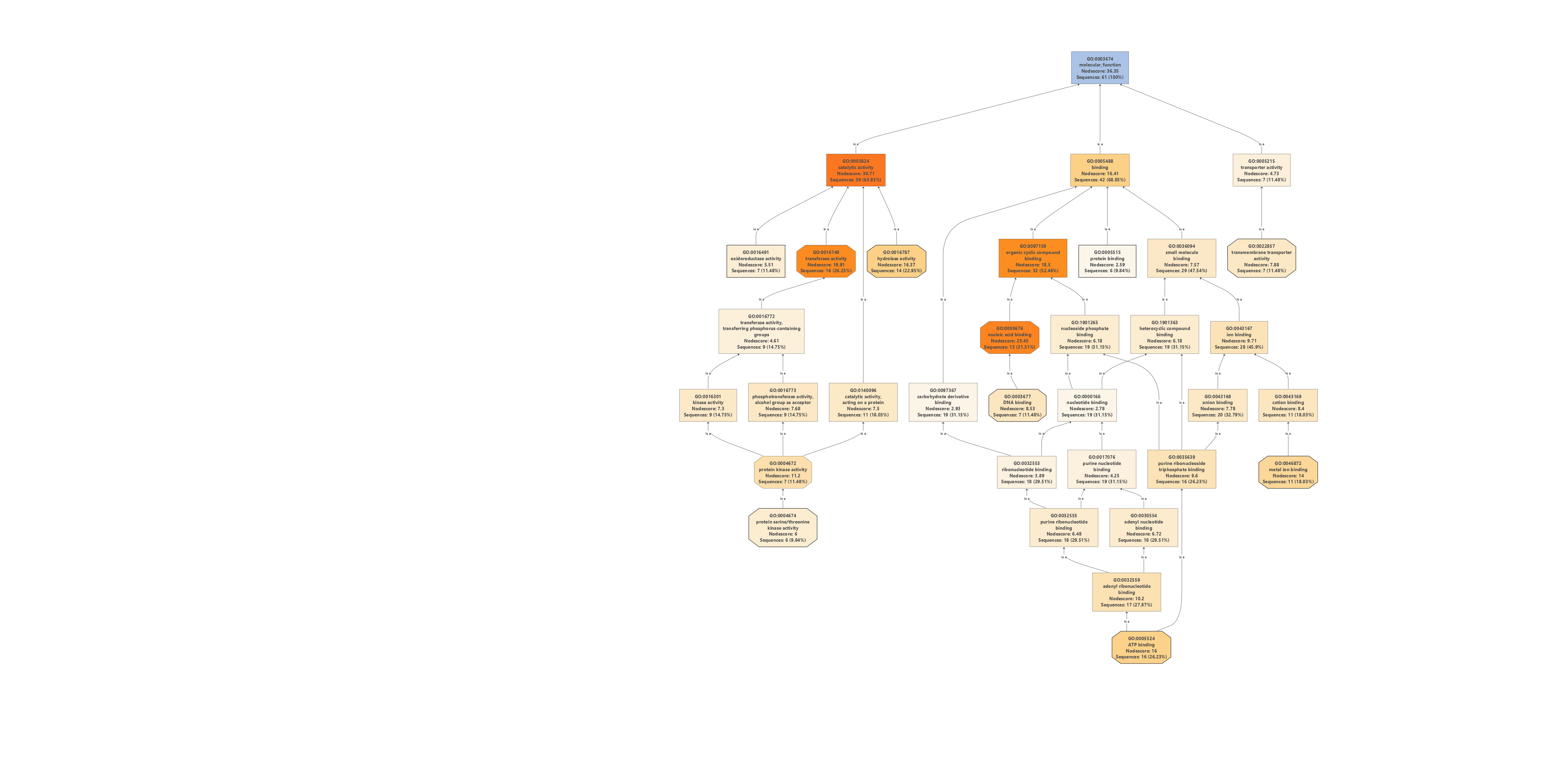
 **Supplementary Figure 4**: GO graph (Molecular function) for DEGs in Frantoia under drought stress compared to control conditions.


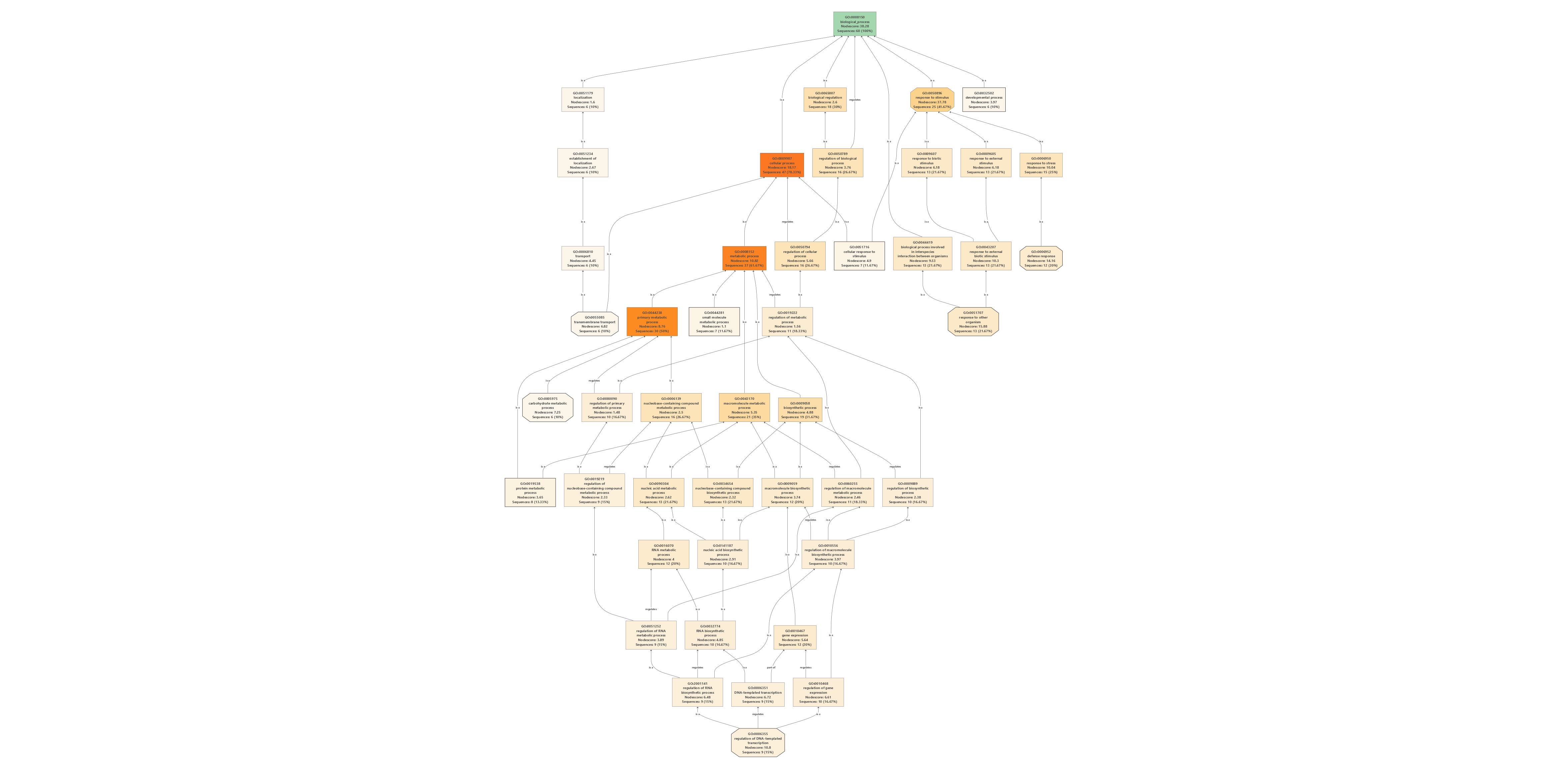
 **Supplementary Figure 5**: GO graph (Biological process) for DEGs in Frantoia under salinity stress compared to control conditions.


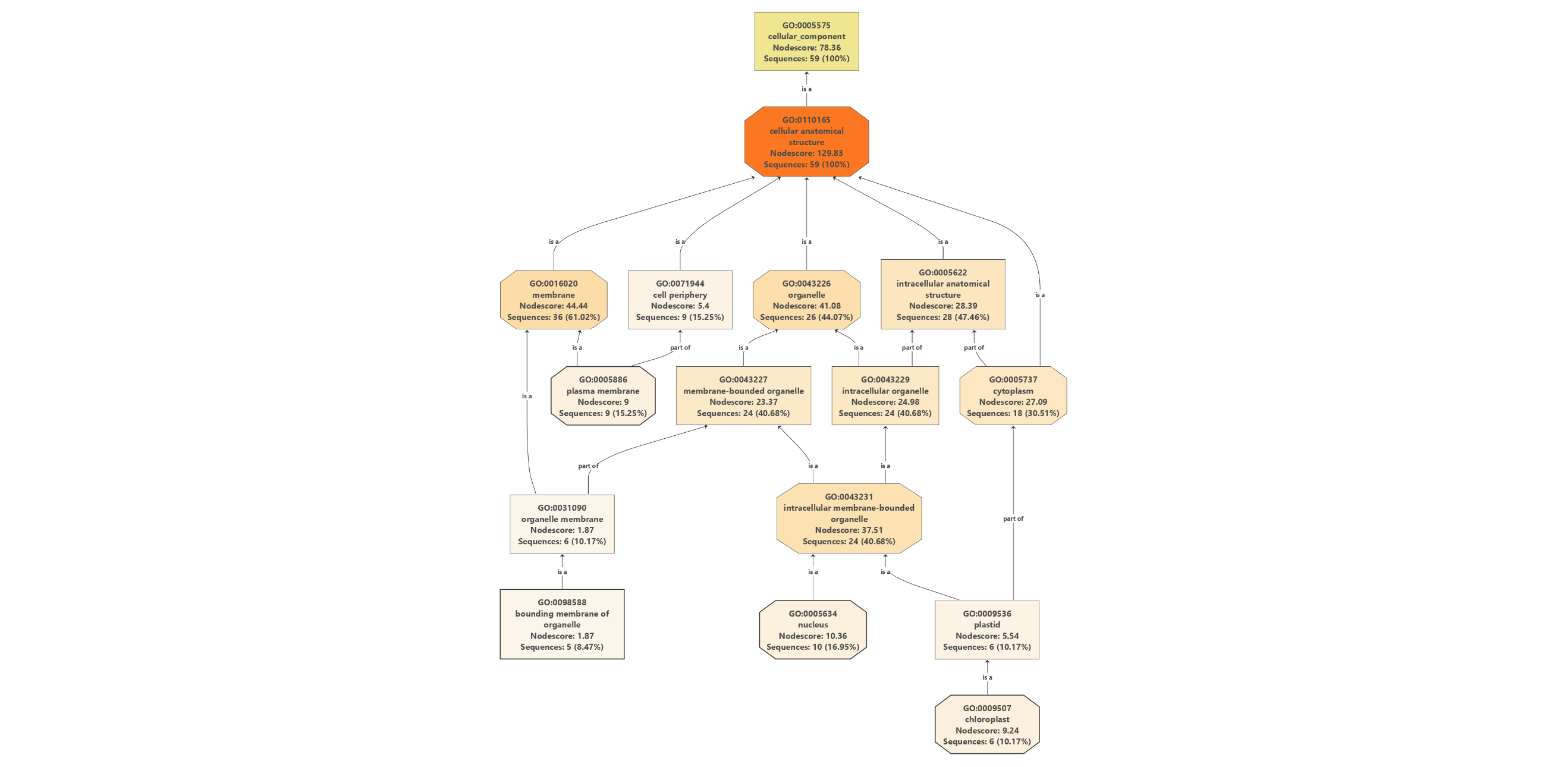


**Supplementary Figure 6**: GO graph (Cellular localization) for DEGs in Frantoia under salinity stress compared to control conditions.


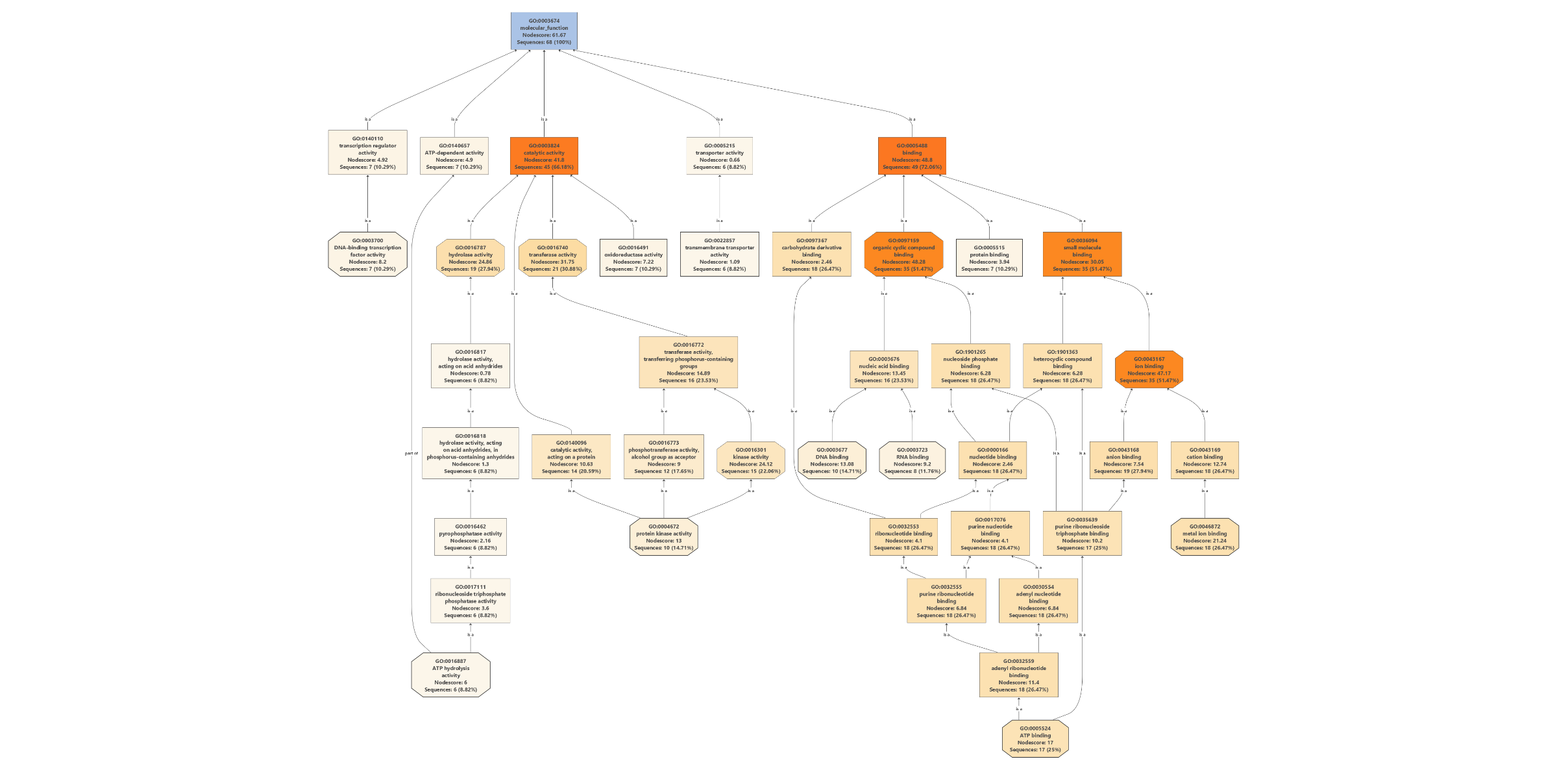


**Supplementary Figure 7**: GO graph (Molecular function) for DEGs in Frantoia under salinity stress compared to control conditions.


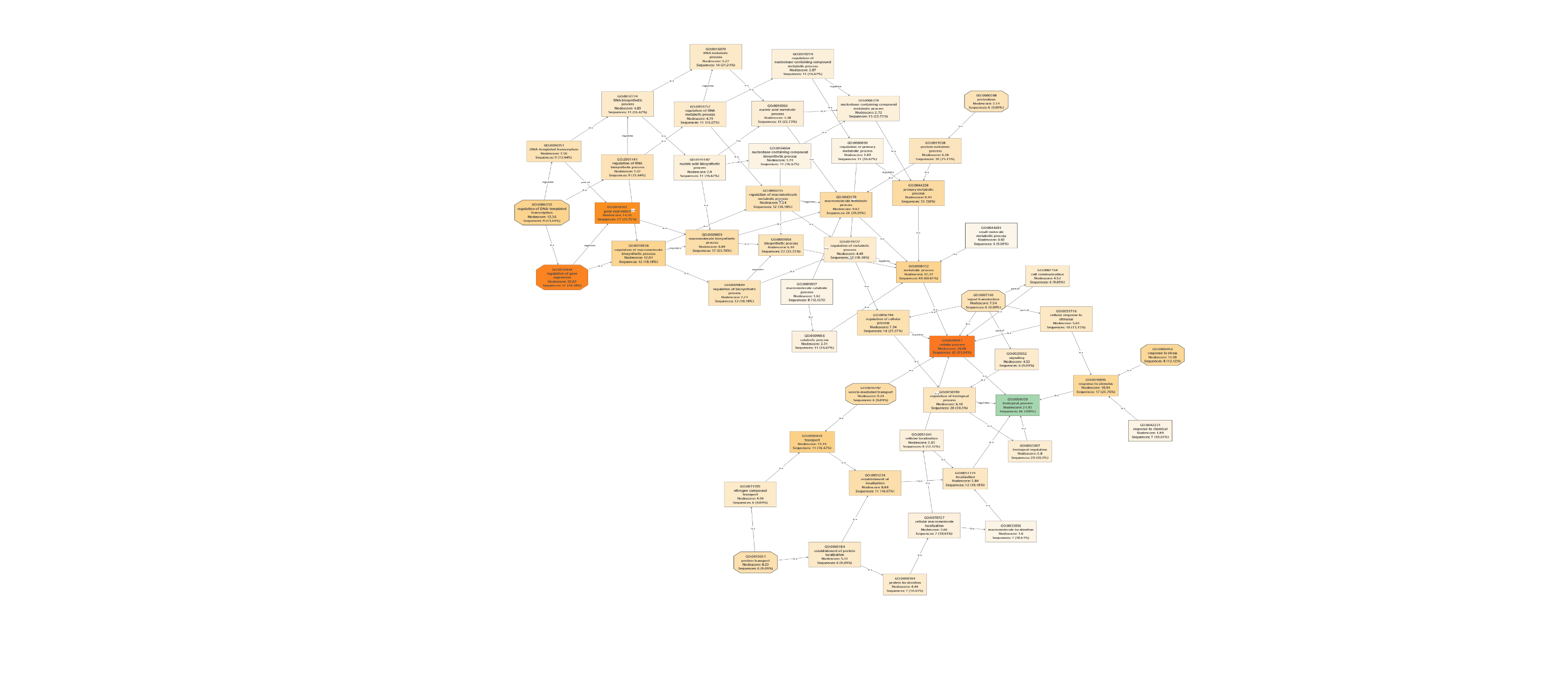


**Supplementary Figure 8**: GO graph (Biological process) for DEGs in Mehras under drought stress compared to control conditions.


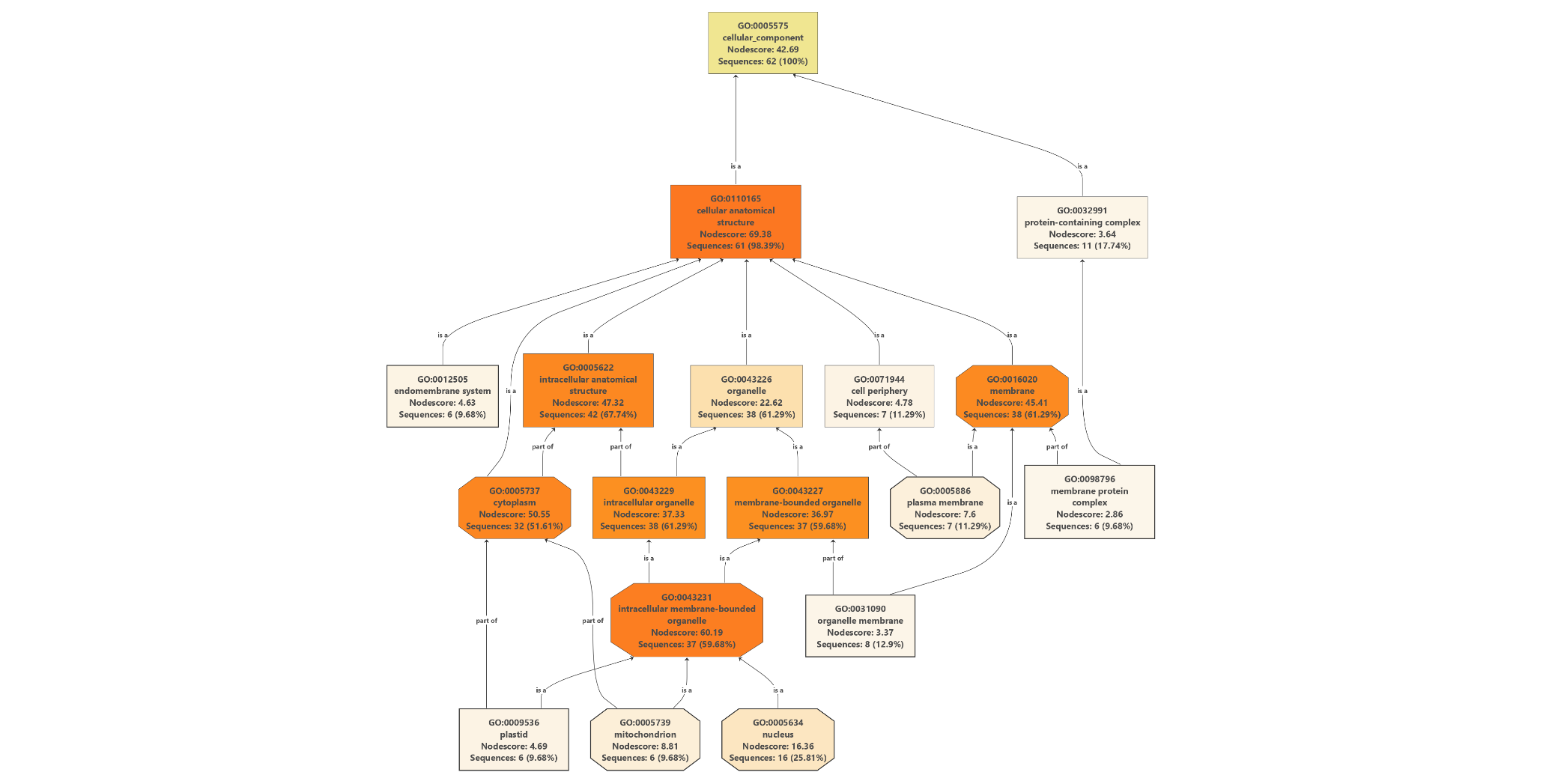


**Supplementary Figure 9**: GO graph (Cellular localization) for DEGs in Mehras under drought stress compared to control conditions.


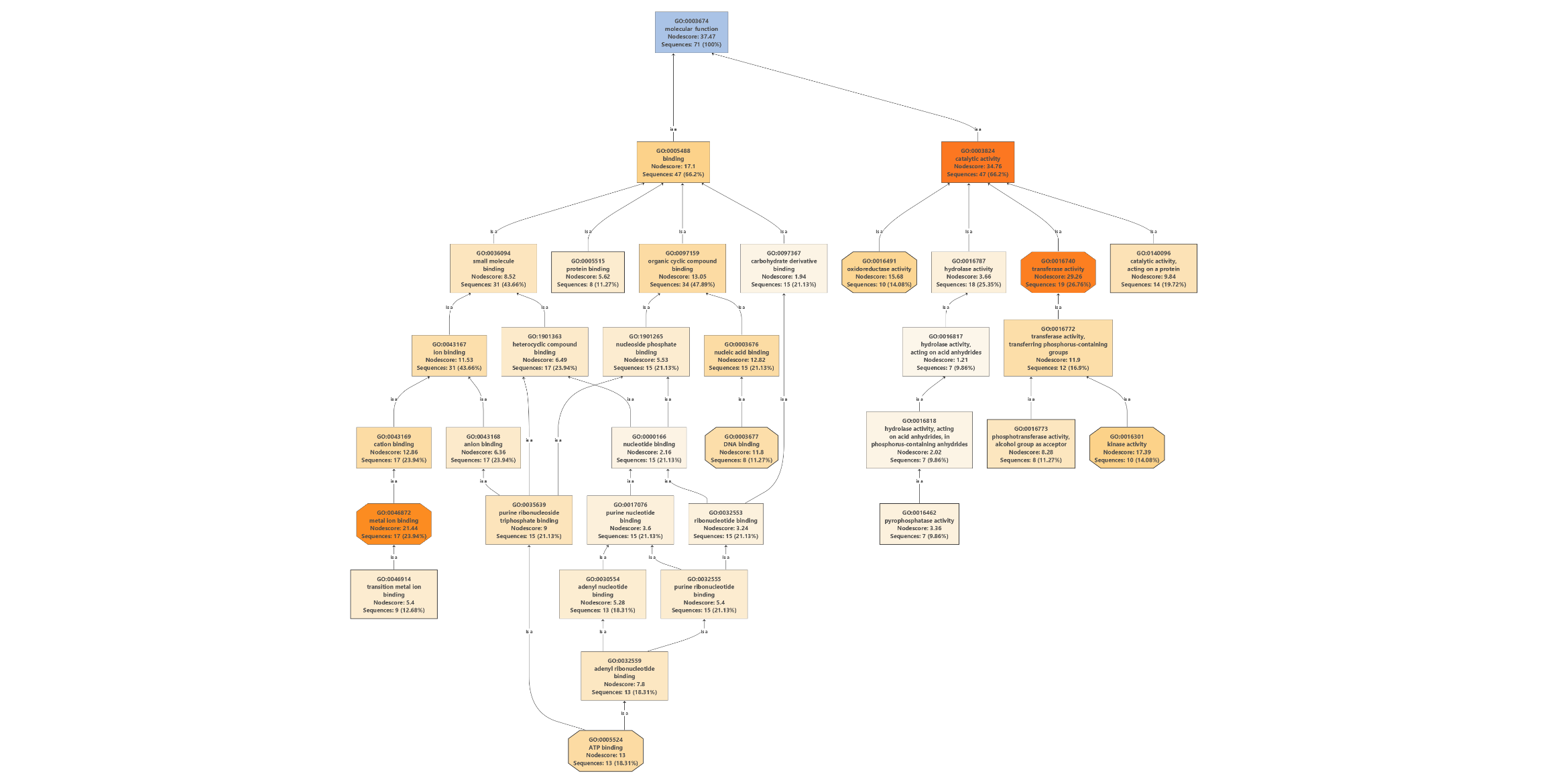


**Supplementary Figure 10**: GO graph (Molecular function) for DEGs in Mehras under drought stress compared to control conditions.


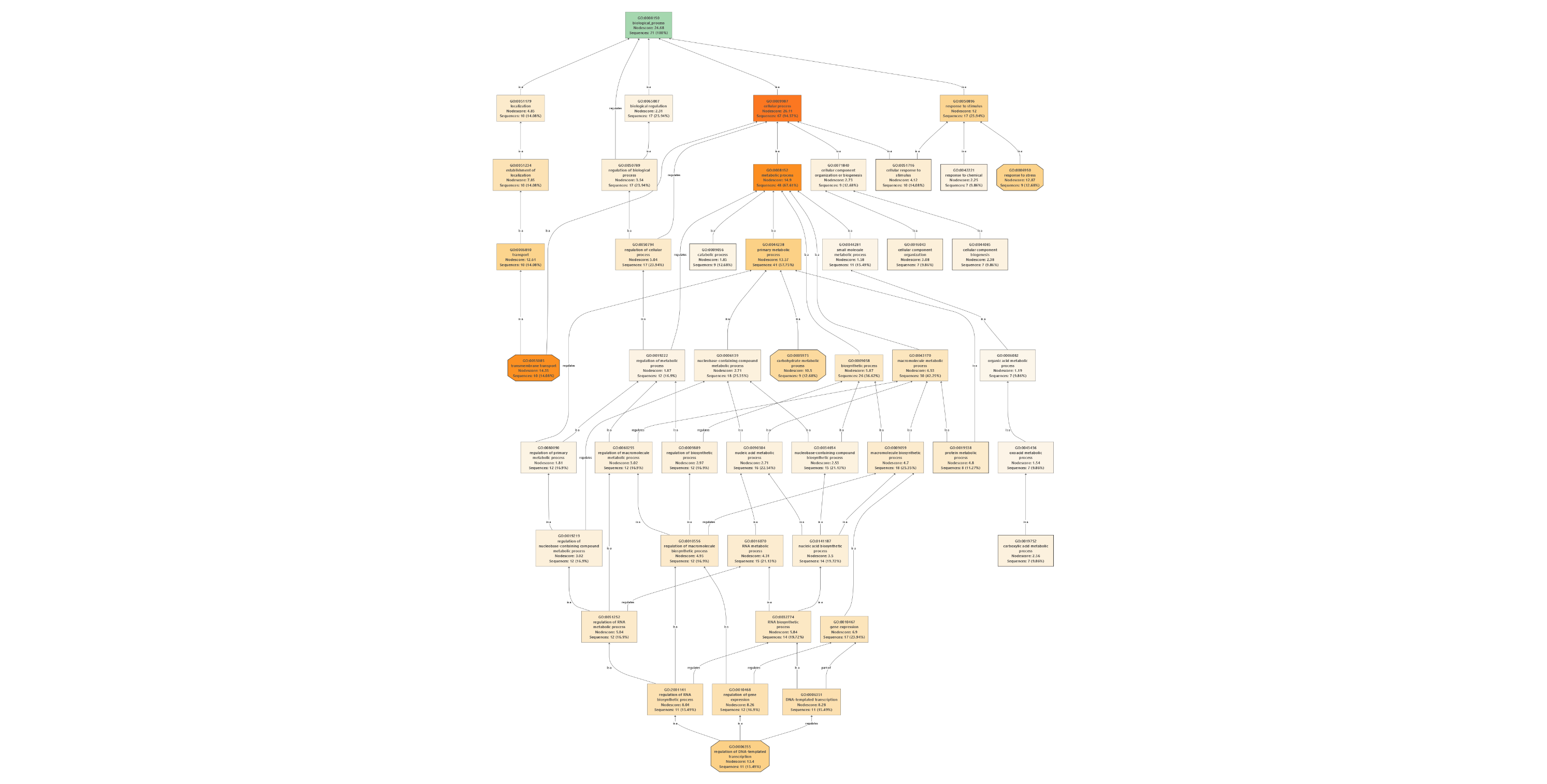


**Supplementary Figure 11**: GO graph (Biological process) for DEGs in Mehras under salinity stress compared to control conditions.


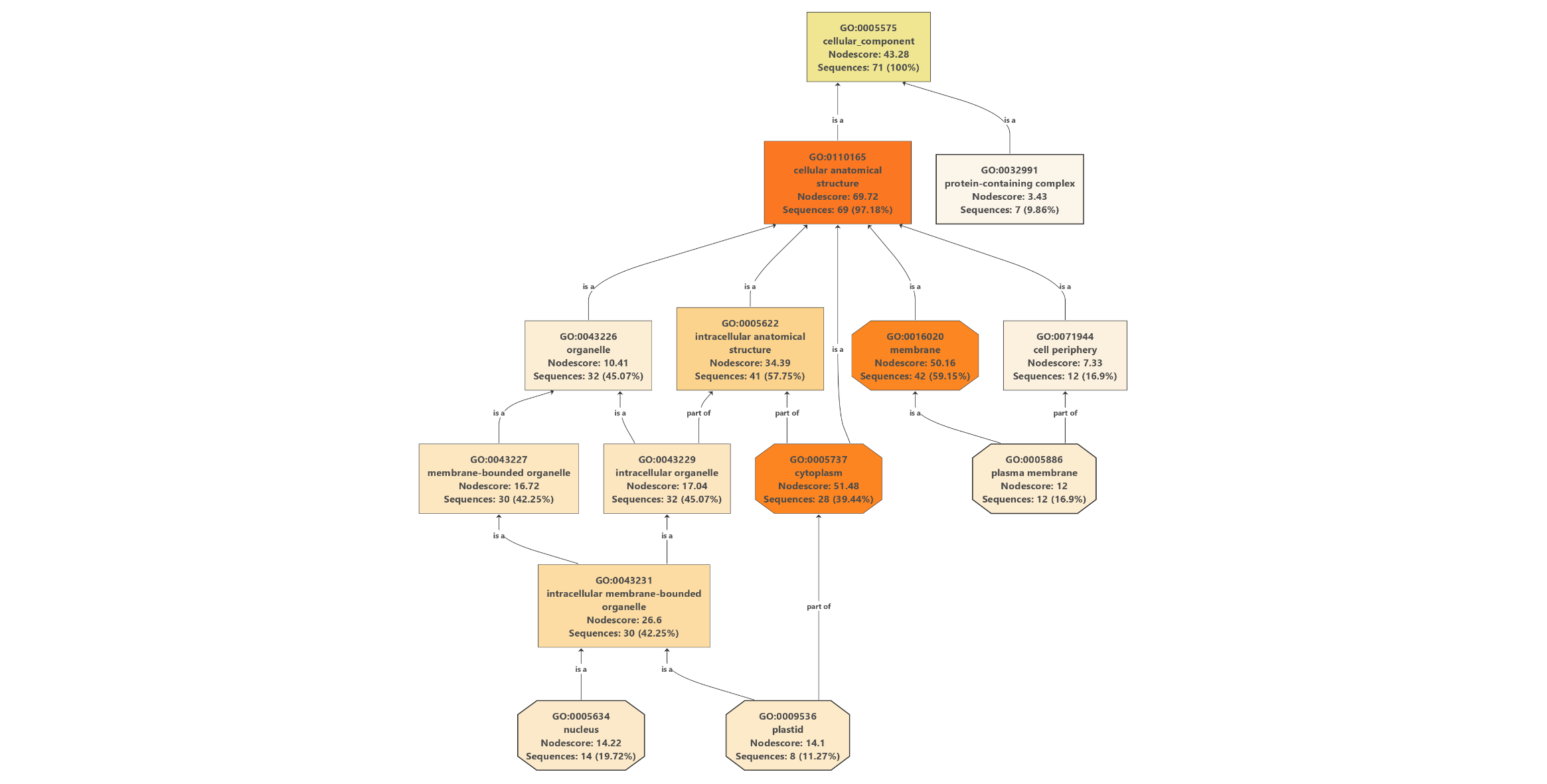


**Supplementary Figure 12**: GO graph (Cellular localization) for DEGs in Mehras under salinity stress compared to control conditions.


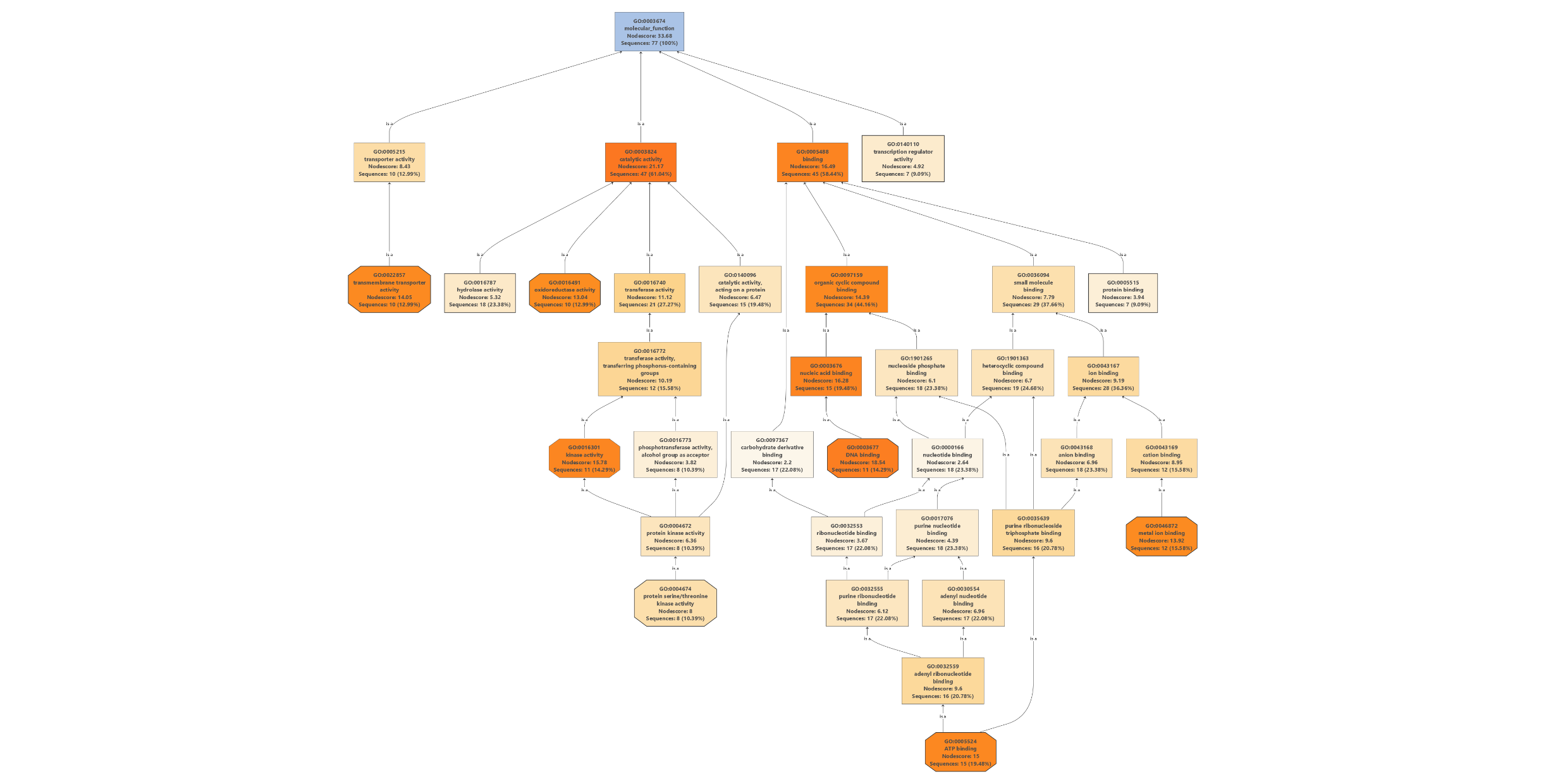


**Supplementary Figure 13**: GO graph (Molecular function) for DEGs in Mehras under salinity stress compared to control conditions.


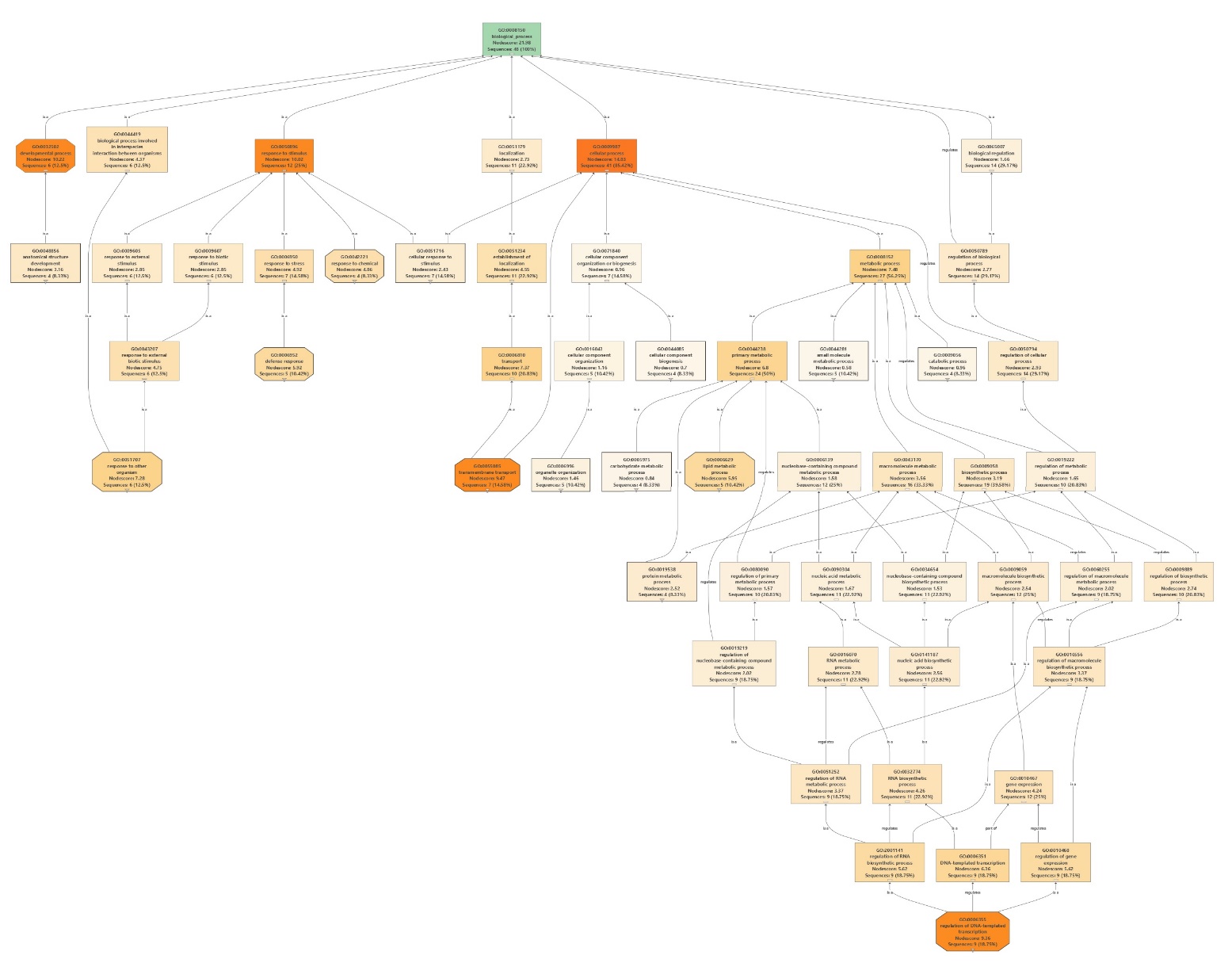


**Supplementary Figure 14**: GO graph (Biological process) for DEGs in Nabali under drought stress compared to control conditions.


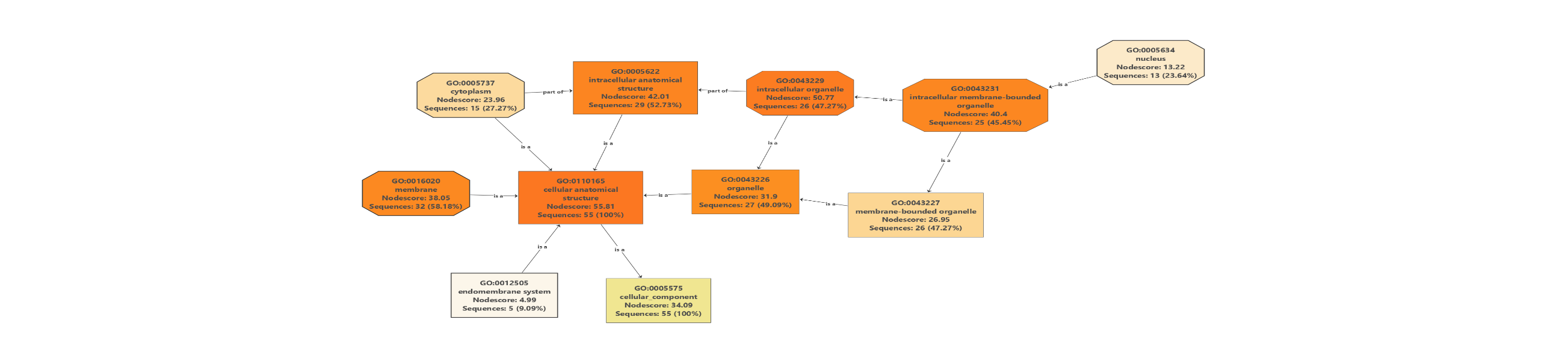


**Supplementary Figure 15**: GO graph (Cellular localization) for DEGs in Nabali under drought stress compared to control conditions.


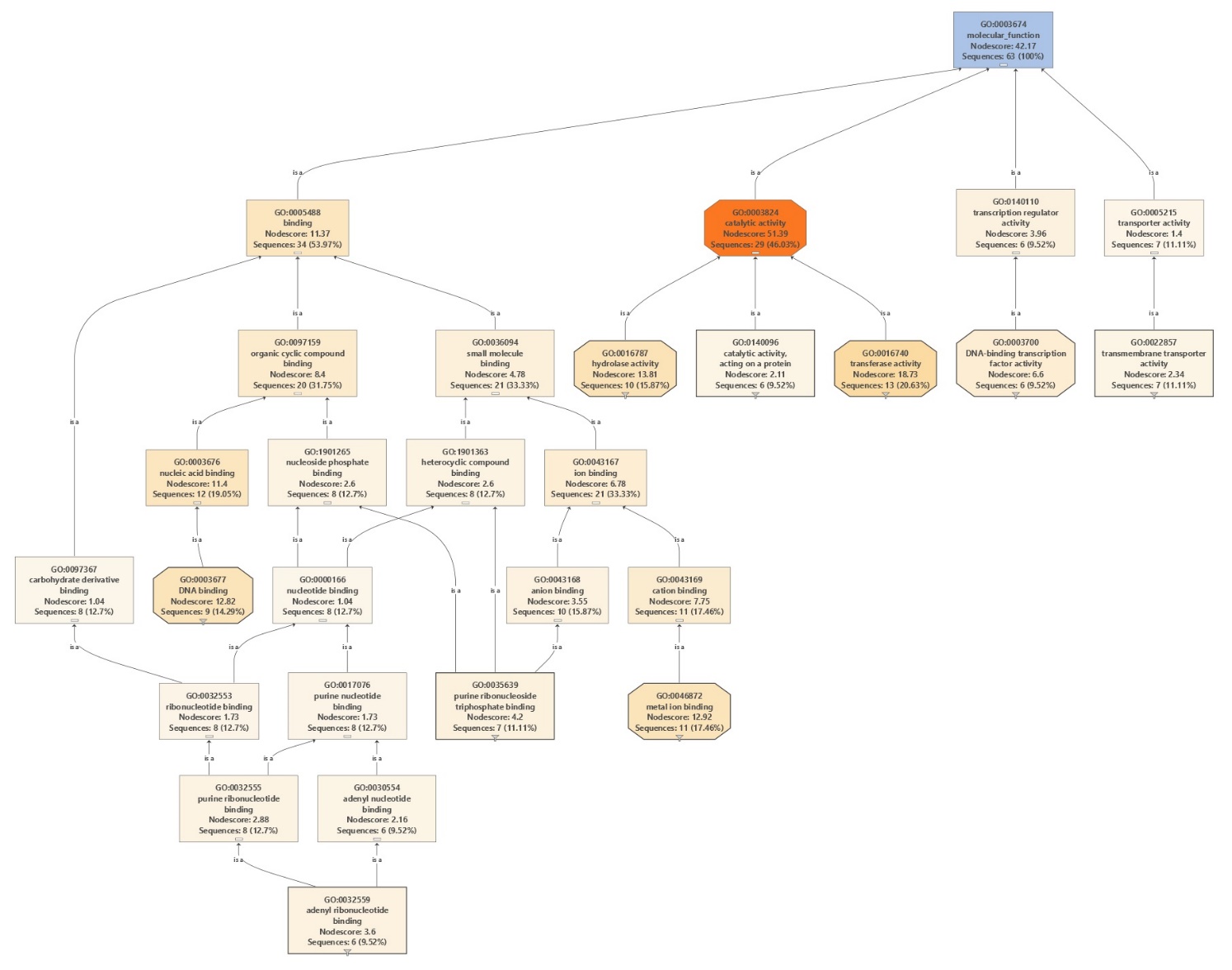


**Supplementary Figure 16**: GO graph (Molecular function) for DEGs in Nabali under drought stress compared to control conditions.


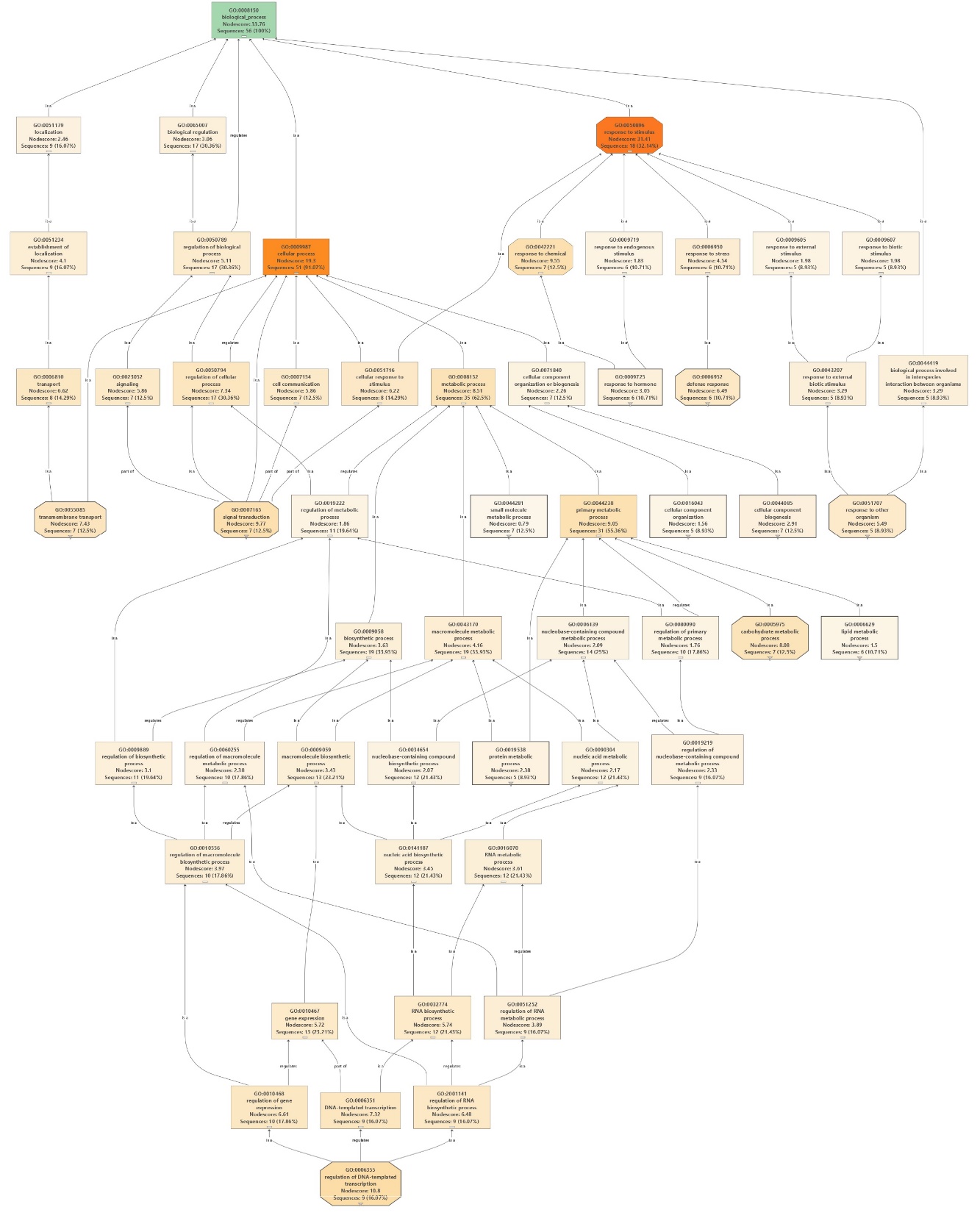


**Supplementary Figure 17**: GO graph (Biological process) for DEGs in Nabali under salinity stress compared to control conditions.


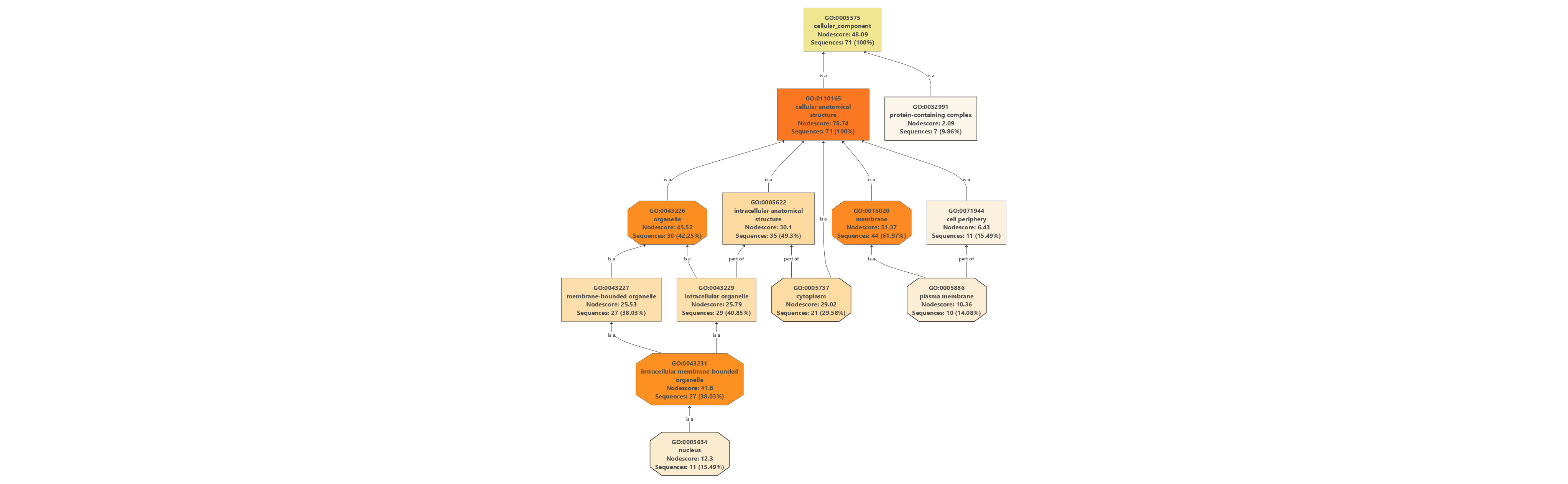


**Supplementary Figure 18**: GO graph (Cellular localization) for DEGs in Nabali under salinity stress compared to control conditions.


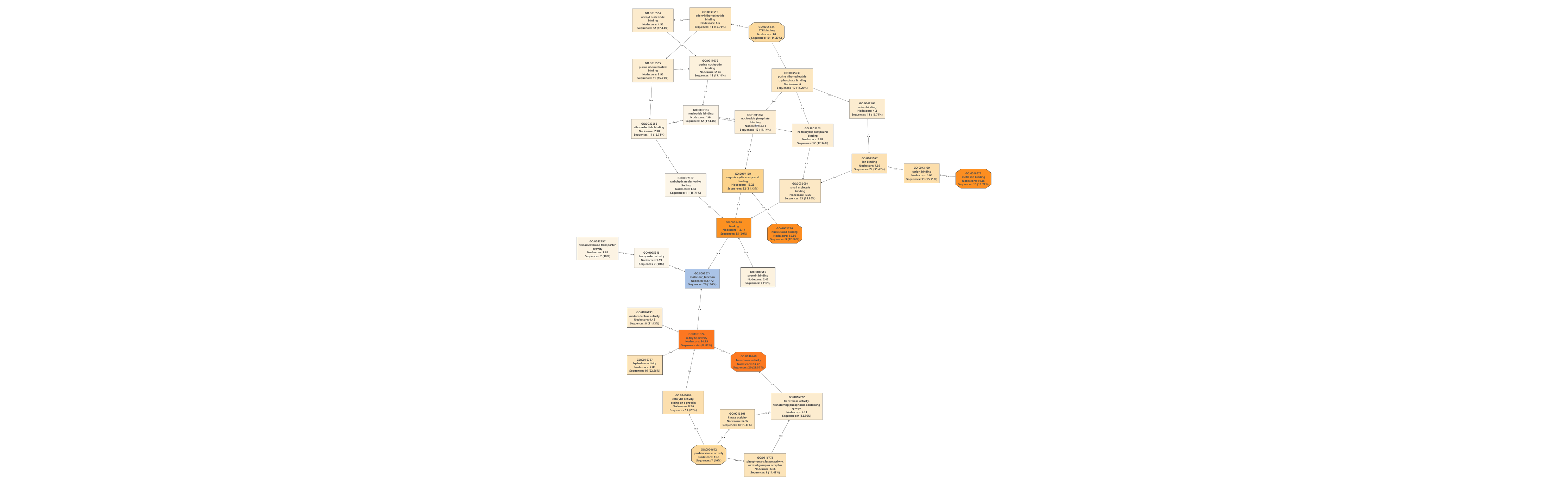


**Supplementary Figure 19**: GO graph (Molecular function) for DEGs in Nabali under salinity stress compared to control conditions.


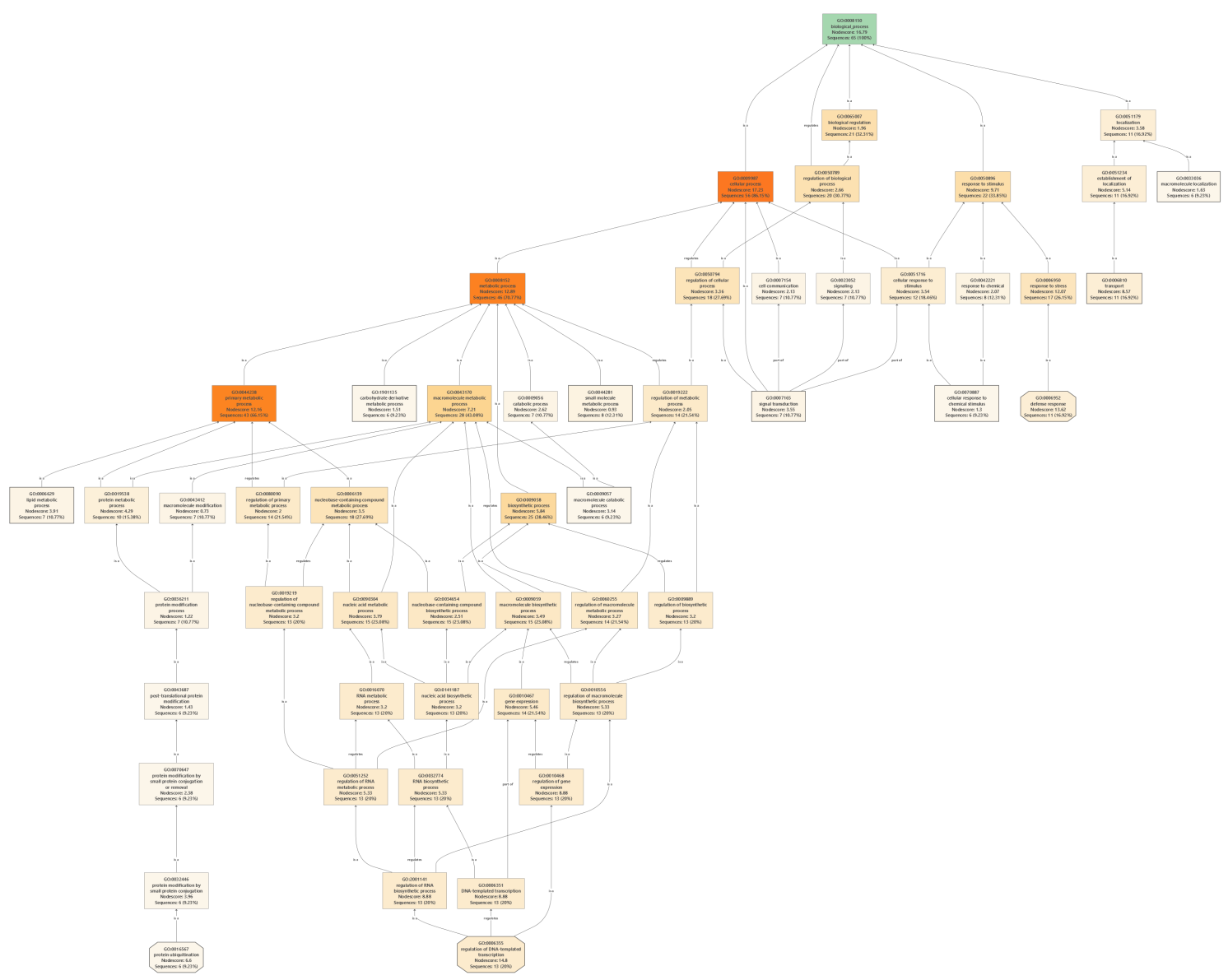


**Supplementary Figure 20**: GO graph (Biological process) for DEGs in Manzanillo under drought stress compared to control conditions.


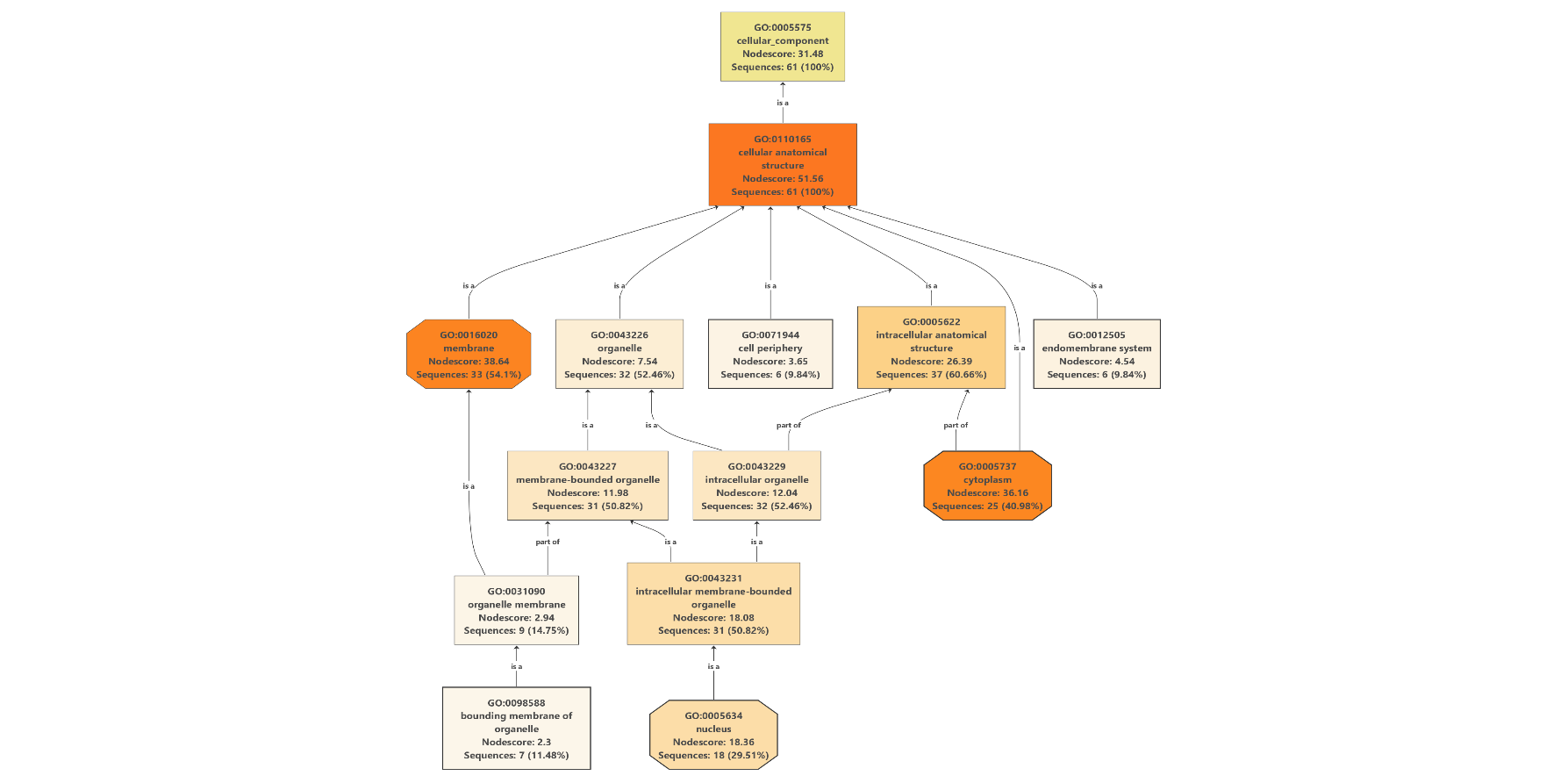


**Supplementary Figure 21**: GO graph (Cellular localization) for DEGs in Manzanillo under drought stress compared to control conditions.


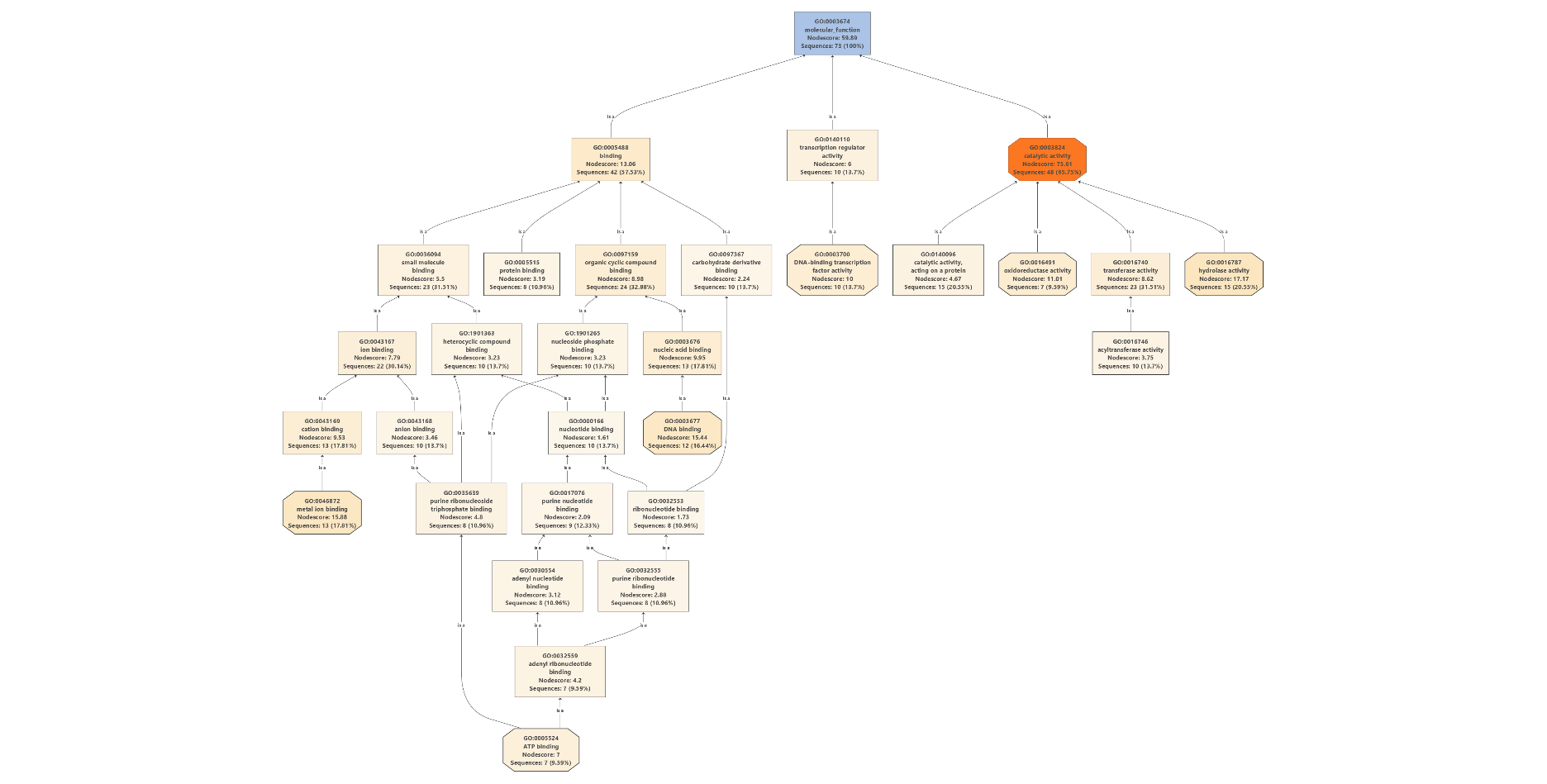


**Supplementary Figure 22**: GO graph (Molecular function) for DEGs in Manzanillo under drought stress compared to control conditions.


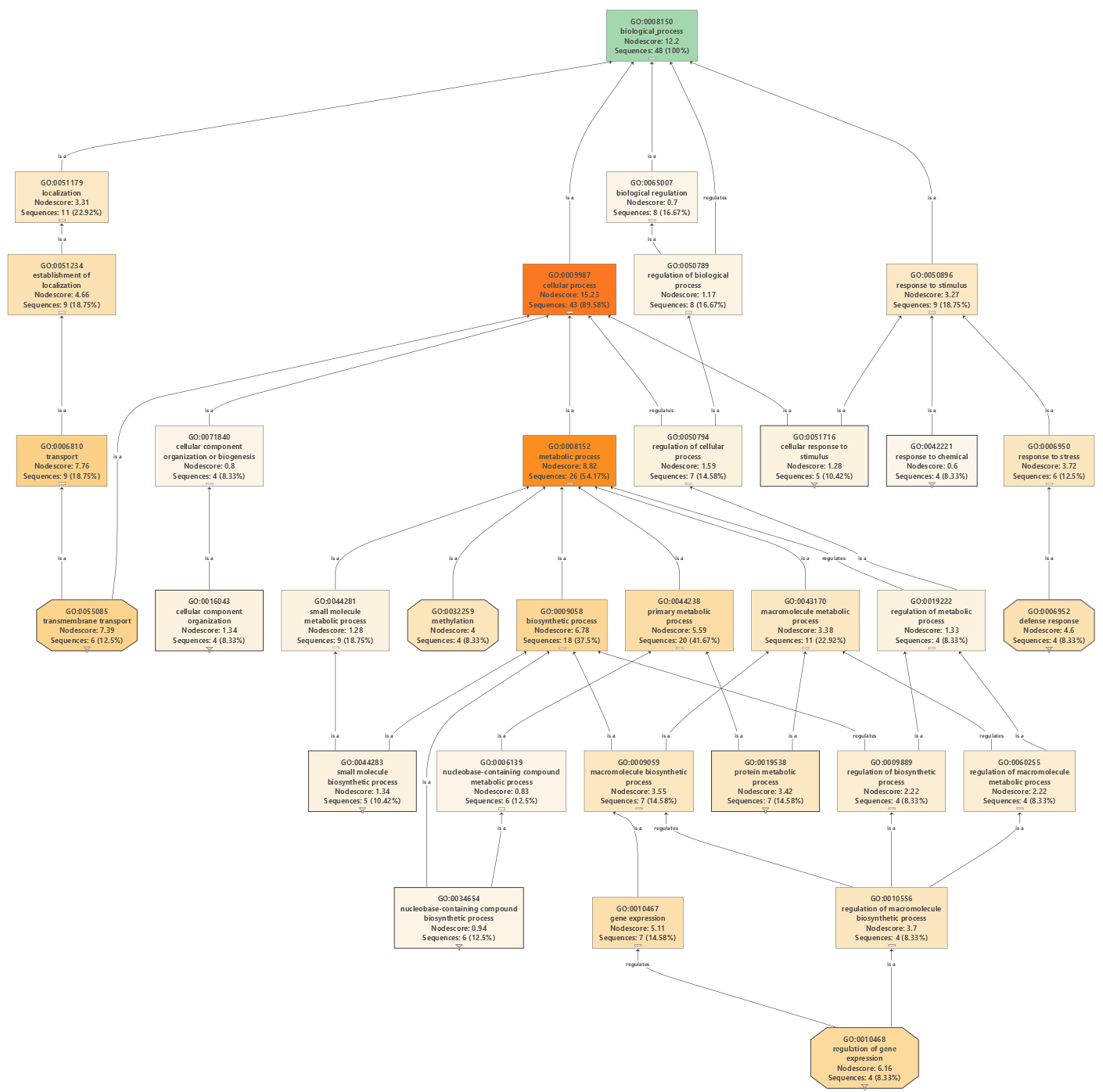


**Supplementary Figure 23**: GO graph (Biological process) for DEGs in Manzanillo under salinity stress compared to control conditions.


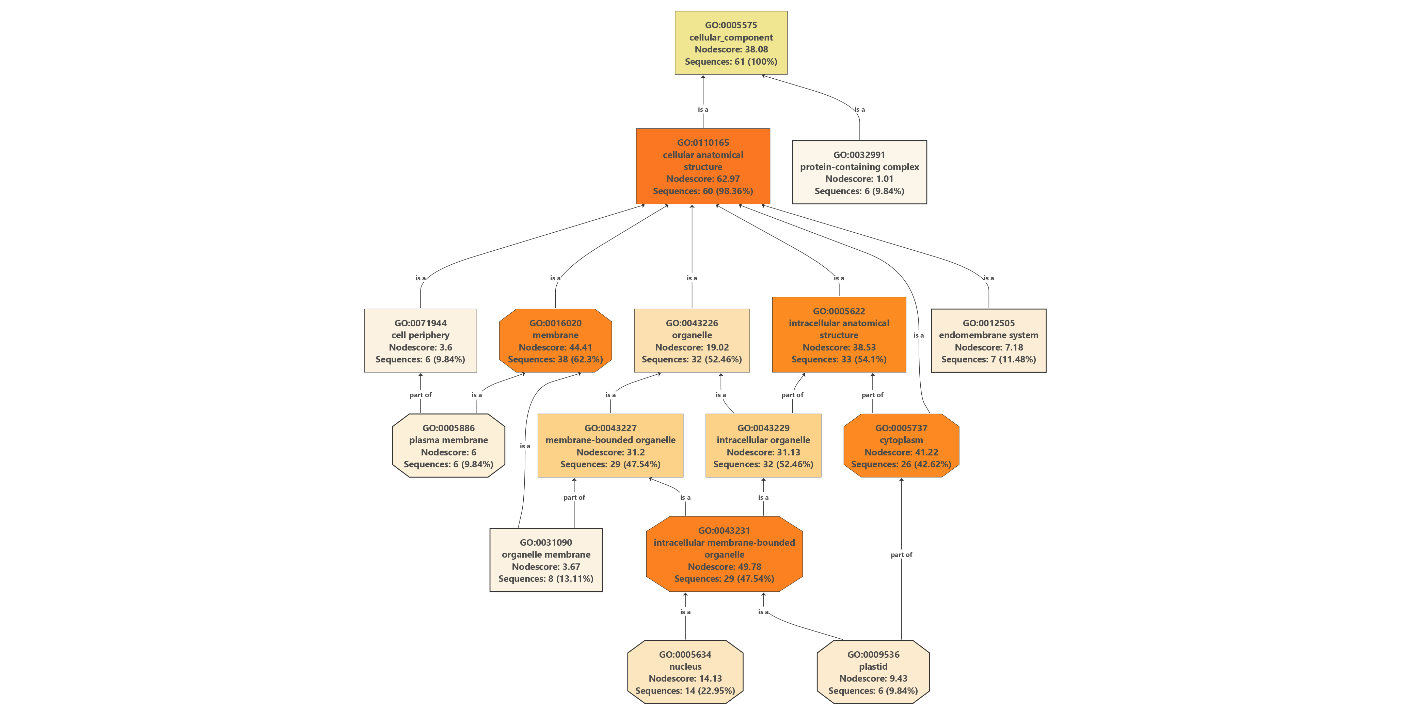


**Supplementary Figure 24**: GO graph (Cellular localization) for DEGs in Manzanillo under salinity stress compared to control conditions.


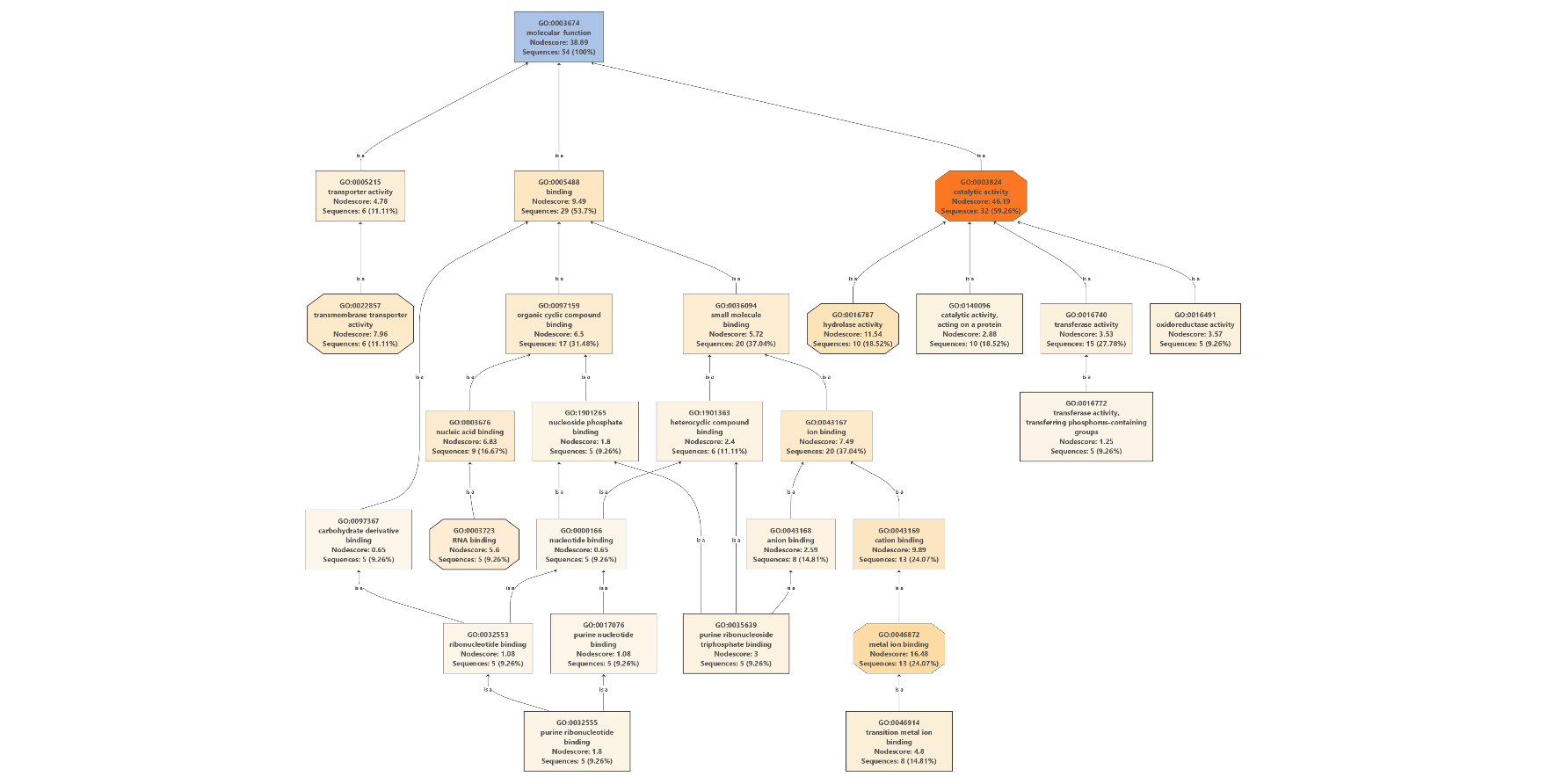


**Supplementary Figure 25**: GO graph (Molecular function) for DEGs in Manzanillo under salinity stress compared to control conditions.

## Supplementary Tables

**Supplementary Tables 1**: Sequence of primers for selected stress responsive genes in olive.

| **#** | **Gene description** | **Gene locus** | **Primer Code** | **Sequence 5′–3′ (Forward and reverse)** | **Tm** | **Size (bp)** |
| --- | --- | --- | --- | --- | --- | --- |
| 1 | WRKY Family Transcription Factor Family Protein | Oeu038715.1 | O.e._F101  O.e._R101 | ACACAACCATGATGTTCTTGCA  AGGGTATACGAAGCATGGTTCT | 59.04  58.96 | 177 |
| 2 | Aspartyl Protease Family Protein | Oeu037859.1 | O.e._F102  O.e._R102 | ATCATCAAAGGGCAATGCTTGT  GATTCGAACTTGGAGGCTGATC | 59.16  59.13 | 173 |
| 3 | Kinase Family Protein | Oeu032895.1 | O.e._F103  O.e._R103 | ACAAAAGGTTACATGGCTCCTG  AGCATCTCCGATAACTCTGTGT | 58.84  58.97 | 155 |
| 4 | Gibberellin 20-Oxidase Family Protein | Oeu033675.1 | O.e._F104  O.e._R104 | CATAGAGCAGTGGTGAACAAGG  GTGGTTTTGGGTGAATTCTTGC | 58.99  58.87 | 159 |
| 5 | Heat Shock Protein | Oeu007780.1 | O.e._F105  O.e._R105 | TTTCCTTCGGTTCACAGTTGAC  TCAGCAATTGTTCCACTTTCTGT | 59.06  59.05 | 169 |
| 6 | Galactokinase Family Protein | Oeu046580.1 | O.e._F106  O.e._R106 | AAGCTTGGTGACCTTATGAACG  AACGATGCTTTCCTTCACCAAG | 58.93  59.45 | 177 |
| 7 | Mate Efflux Family Protein | Oeu021967.1 | O.e._F107  O.e._R107 | CCTTAGCTGTGATTTTGGGTGT  ATCATAGAACGTGCCGCATAAG | 58.84  58.94 | 193 |
| 8 | Ubiquitin-Protein | Oeu040176.1 | O.e._F108  O.e._R108 | ACTCTTCACCTGTCTGACATGT  CCACTGTTTTGAGCTGAGTCAT | 59.03  58.86 | 193 |
| 9 | Serine Threonine-Protein | Oeu026361.1 | O.e._F109  O.e._R109 | TAAATAGGGGTGTAGTTCGGGT  CCACCATCTTGCTGAATTCCAA | 58.28  59.17 | 150 |
| 10 | Homeobox Associated Leucine Zipper Protein | Oeu012394.1 | O.e._F110  O.e._R110 | ACGCAACGCTAAATCAGATTCT  ATCTTCATGTGGGCTCTGATGA | 58.74  59.22 | 175 |
| 11 | Alcohol Dehydrogenase Expressed | Oeu022027.2 | O.e._F111  O.e._R111 | ATGGTTGGGGATTAACGGTAGT  CAACTTCACCTCCCCATTCATG | 59.15  59.24 | 176 |
| 12 | AP2 Domain-Containing TF | Oeu051802.2 | O.e._F112  O.e._R112 | CATTGACGCAAAACTACAAGCC  CGACCCAAATCCTCCATTCAAT | 59.02  58.71 | 175 |
| 13 | Lemir Os=Solanum Lycopersicum | Oeu032705.1 | O.e._F113  O.e._R113 | ATTCGATGCCAGCAATCAGAAA  GCAATCTTTACACACTGTCGGA | 58.98  58.94 | 157 |
| 14 | Potassium Efflux Antiporter | Oeu030165.3 | O.e._F114  O.e._R114 | TTACTGGCGCAAATTGGAGAAT  CCAATGCATAAGAACTCCCAGG | 58.91  59.05 | 180 |
| 15 | Proline-Rich Family Protein | Oeu022984.1 | O.e._F115  O.e._R115 | GCTGATATCATCTCAAGTGGCC  CATCCTCGTTTTCCCAACTTGA | 58.93  58.85 | 198 |
| 16 | PPR Containing Plant-Like Protein | Oeu060351.1 | O.e._F116  O.e._R116 | TCAGGGAAGGTGGTTGAAGTTA  CACTATAAGGCACCATGACACG | 58.95  59.13 | 187 |
| 17 | Enox5 Os=Prunus Persica | Oeu000296.1 | O.e._F117  O.e._R117 | GCCGGCTTTATTTCGTCTTTTG  TCTGTGATTTGAAGACCTGGGT | 59.09  59.29 | 170 |
| 18 | Peptidyl-Prolyl Cis-Trans Isomerase | Oeu049917.2 | O.e._F118  O.e._R118 | GGTTCTCCTCATGTTGTTGCA  CTGCCATCCTGTTTTGCCTT | 58.77  59.03 | 230 |
| 19 | Rap2-Like Protein | Oeu013901.1 | O.e._F119  O.e._R119 | GAAATACAAAGGCATTCGACGC  TTTCCGGTGATGAAATTCCTCG | 58.90  58.99 | 167 |
| 20 | AP2 Erf Domain Transcription Factor | Oeu013711.1 | O.e._F120  O.e._R120 | GATAGGGCGGCGATTGAATTTA  CCCAACTTCCATTTCTGTCGAA | 58.93  58.85 | 189 |
| 21 | WRKY Transcription Factor 6 Family Protein | Oeu051201.1 | O.e._F121  O.e._R121 | AGTAGTACTGAAGCTGCCTCAT  TACTGTTGTTTGGATTGGAGCC | 58.63  58.85 | 181 |
| 22 | Heat Shock Protein | Oeu007780.1 | O.e._F122  O.e._R122 | GCAGTAGTTTCAGGTTCCGAAA  CTCTGCACCTTTTGTTTCTGGA | 58.86  59.05 | 172 |
| 23 | Alcohol Dehydroge Family Protein | Oeu009320.2 | O.e._F123  O.e._R123 | GACACAGGAAAGGAGAAAGTGG  TTTAGGCCTTCTCTCAGAGCAA | 58.86  59.09 | 194 |
| 24 | Heat Shock Factor Protein | Oeu043983.1 | O.e._F124  O.e._R124 | AACAGATGGAGACTTAGTGCCT  AAGCTGGCCATGATTTTCGTAA | 58.82  58.91 | 169 |
| 25 | Actin 1 | Sadder b et al. (2021) | O.e._F125  O.e._R125 | TTAATAGGAATGGAATCTGCAGGAATCC  CAAGAGCTGTAATTTCCTTACTCATGCG |  | 167 |
| 26 | Actin 11 |  | O.e._F126  O.e_R.126 | CCCAAGGCCAACAGAGAGAA  GGAAAGAACGGCCTGAATAGC |  |  |

**Supplementary Tables 2**: Top 100 up-regulated DEGs in olive cultivar ‘Frantoia’ under drought relative to control with at least 2 folds.

| # | **Feature ID** | **Annotations – Description** | **F_d/c** |
| --- | --- | --- | --- |
| 1 | Oeu032705.1 | lemir | 5.535428 |
| 2 | Oeu025312.1 | phytosulfokines 3 family protein | 4.961436 |
| 3 | Oeu056985.2 |  | 4.823933 |
| 4 | Oeu018758.1 | kunitz-type protease inhibitor kpi- | 4.430706 |
| 5 | Oeu007004.1 | trna synthetase class i family protein | 4.145861 |
| 6 | Oeu063387.1 | verticillium wilt disease resistance protein | 4.025567 |
| 7 | Oeu029537.1 | #NAME? | 3.961436 |
| 8 | Oeu038980.1 | gland-specific fatty acyl- reductase | 3.894322 |
| 9 | Oeu061787.1 | pentatricopeptide repeat-containing protein at2g27610 | 3.894322 |
| 10 | Oeu004512.1 |  | 3.823933 |
| 11 | Oeu037859.1 | aspartyl protease family protein | 3.823933 |
| 12 | Oeu052209.1 | mif4g domain-containing family protein | 3.787407 |
| 13 | Oeu051410.2 | carboxypeptidase | 3.749932 |
| 14 | Oeu058293.1 | abc transporter family protein | 3.749932 |
| 15 | Oeu062637.1 | metallo-beta-lactamase family protein | 3.749932 |
| 16 | Oeu018241.1 | atp-dependent 6-phosphofructokinase | 3.711458 |
| 17 | Oeu038715.1 | wrky family transcription factor family protein | 3.67193 |
| 18 | Oeu004236.1 | abc transporter family protein | 3.589467 |
| 19 | Oeu020267.1 |  | 3.589467 |
| 20 | Oeu048962.1 | transcription factor kan2 | 3.589467 |
| 21 | Oeu040105.1 | glycosyl hydrolase family 17 family protein | 3.524372 |
| 22 | Oeu058879.1 | os02g0828533 protein | 3.502005 |
| 23 | Oeu030072.1 | hxxxd-type acyl-transferase family protein | 3.456201 |
| 24 | Oeu001751.1 | ring-h2 finger protein atl47 | 3.408895 |
| 25 | Oeu002537.1 | diacylglycerol kinase | 3.408895 |
| 26 | Oeu005927.1 | sam domain protein | 3.408895 |
| 27 | Oeu013490.1 | gdp-fucose o-fucosyltransferase-like protein | 3.408895 |
| 28 | Oeu015770.1 | leucine-rich repeat family protein | 3.408895 |
| 29 | Oeu032782.1 | chaperone bcs1-a-like | 3.408895 |
| 30 | Oeu039278.1 | homocysteine s-methyltransferase 1 family protein | 3.408895 |
| 31 | Oeu050383.1 | wall associated kinase-like protein | 3.408895 |
| 32 | Oeu052764.1 | patatin | 3.408895 |
| 33 | Oeu035793.1 | male sterility ms5 family protein | 3.38953 |
| 34 | Oeu026057.1 |  | 3.359986 |
| 35 | Oeu033042.1 |  | 3.359986 |
| 36 | Oeu011957.1 | ef hand calcium-binding family protein | 3.30936 |
| 37 | Oeu025260.1 | glutathione s-transferase t3 | 3.30936 |
| 38 | Oeu033333.1 | pre-mrna cleavage complex-related family protein | 3.30936 |
| 39 | Oeu037148.1 |  | 3.30936 |
| 40 | Oeu061430.1 | desacetoxyvindoline -4 | 3.30936 |
| 41 | Oeu032776.1 | chromo domain protein lhp1 | 3.256892 |
| 42 | Oeu039449.1 | d-3-phosphoglycerate dehydrogenase family protein | 3.256892 |
| 43 | Oeu036009.4 | cytidine deoxycytidylate deaminase family protein | 3.202444 |
| 44 | Oeu056538.1 |  | 3.202444 |
| 45 | Oeu003374.1 | os03g0707200 protein | 3.202444 |
| 46 | Oeu020156.3 | abc transporter family protein | 3.202444 |
| 47 | Oeu022295.1 |  | 3.202444 |
| 48 | Oeu022529.1 | basix helix-loop-helix family protein | 3.202444 |
| 49 | Oeu047783.2 |  | 3.202444 |
| 50 | Oeu055000.1 | armadillo beta-catenin repeat protein | 3.202444 |
| 51 | Oeu058771.1 | inactive lrr receptor-like kinase | 3.202444 |
| 52 | Oeu062817.1 | s-norcoclaurine synthase-like protein | 3.202444 |
| 53 | Oeu063395.1 |  | 3.202444 |
| 54 | Oeu017474.1 | 3 2 protein | 3.16497 |
| 55 | Oeu002701.1 |  | 3.157356 |
| 56 | Oeu024078.1 |  | 3.145861 |
| 57 | Oeu009134.1 |  | 3.086967 |
| 58 | Oeu009479.1 | abc transporter family protein | 3.086967 |
| 59 | Oeu013186.1 | cytochrome p450 family protein | 3.086967 |
| 60 | Oeu022457.1 | integrin-linked protein kinase-like protein | 3.086967 |
| 61 | Oeu023781.1 | serine threonine-protein kinase | 3.086967 |
| 62 | Oeu024376.1 | zinc finger family protein | 3.086967 |
| 63 | Oeu028423.2 | biotin carboxylase chloroplastic | 3.086967 |
| 64 | Oeu031815.1 | 40s ribosomal protein s15a | 3.086967 |
| 65 | Oeu035044.1 | proton-dependent oligopeptide transport family protein | 3.086967 |
| 66 | Oeu035561.1 | ppr containing plant-like protein | 3.086967 |
| 67 | Oeu044008.1 | pentatricopeptide repeat-containing protein at2g01740 | 3.086967 |
| 68 | Oeu049484.1 | alcohol dehydrogenase-like protein | 3.086967 |
| 69 | Oeu060769.1 | 4-alpha-glucanotransferase-related family protein | 3.086967 |
| 70 | Oeu062143.1 | cyclic nucleotide-gated ion channel | 3.086967 |
| 71 | Oeu017831.1 | thaliana gn=oxs3 pe=2 sv=1 | 3.048493 |
| 72 | Oeu009496.1 | os02g0236500 protein | 3.025567 |
| 73 | Oeu030174.1 | glycoside hydrolase family 18 protein | 3.025567 |
| 74 | Oeu040281.1 | exocyst subunit exo70 family protein | 3.025567 |
| 75 | Oeu057312.1 | cytochrome p450 family 71 protein | 3.025567 |
| 76 | Oeu010311.1 |  | 2.994963 |
| 77 | Oeu040839.2 | at5g47580 mnj7_17 | 2.993858 |
| 78 | Oeu003671.1 | pentatricopeptide repeat-containing family protein | 2.961436 |
| 79 | Oeu004608.1 | gdp-fucose o-fucosyltransferase-like protein | 2.961436 |
| 80 | Oeu008015.1 | rcd1-like cell differentiation family protein | 2.961436 |
| 81 | Oeu015671.1 | chlorophyllase 2 | 2.961436 |
| 82 | Oeu022687.1 | transmembrane | 2.961436 |
| 83 | Oeu029659.1 | adenine nucleotide alpha hydrolases-domain containing protein kinase | 2.961436 |
| 84 | Oeu030610.1 | dre-binding protein 5 | 2.961436 |
| 85 | Oeu031504.1 | dna-binding family protein | 2.961436 |
| 86 | Oeu032895.1 | kinase family protein | 2.961436 |
| 87 | Oeu034528.1 |  | 2.961436 |
| 88 | Oeu035729.1 |  | 2.961436 |
| 89 | Oeu040027.1 | pollen ole e i family allergen | 2.961436 |
| 90 | Oeu042295.1 |  | 2.961436 |
| 91 | Oeu047006.1 | ascorbate oxidase promoter-binding family protein | 2.961436 |
| 92 | Oeu050726.1 | endomembrane protein 70 | 2.961436 |
| 93 | Oeu054479.1 | pathogenesis-related thaumatin family protein | 2.961436 |
| 94 | Oeu055686.1 |  | 2.961436 |
| 95 | Oeu059859.1 |  | 2.961436 |
| 96 | Oeu062256.1 |  | 2.961436 |
| 97 | Oeu015422.1 | zinc finger family protein | 2.937072 |
| 98 | Oeu031743.1 | serine threonine-protein phosphatase | 2.894322 |
| 99 | Oeu049760.1 | mads-box transcription factor family protein | 2.894322 |
| 100 | Oeu007324.1 | chaperone protein 11 | 2.890702 |

**Supplementary Tables 3:** Top 100 up-regulated DEGs in olive cultivar ‘Frantoia’ under salinity relative to control with at least 2 folds**.**

| # | **Feature ID** | **Annotations – Description** | **F_s/c** |
| --- | --- | --- | --- |
| 1 | Oeu016087.1 |  | 5.941692 |
| 2 | Oeu033675.1 | gibberellin 20-oxidase family protein | 5.791275 |
| 3 | Oeu002507.1 | beta-galactosidase | 5.587261 |
| 4 | Oeu037614.1 |  | 5.568883 |
| 5 | Oeu007811.1 | duf1005 family protein | 5.512299 |
| 6 | Oeu057085.2 | rna recognition motif-containing family protein | 5.233015 |
| 7 | Oeu004303.1 | eukaryotic aspartyl protease family protein | 5.202344 |
| 8 | Oeu031613.1 | Putative uncharacterized protein | 5.06484 |
| 9 | Oeu021568.1 | chitinase 1 | 5.05607 |
| 10 | Oeu032705.1 | lemir | 5.028314 |
| 11 | Oeu059690.1 | lrr receptor-like kinase family protein | 4.927337 |
| 12 | Oeu031349.1 | ribulose bisphosphate carboxylase oxygenase chloroplastic | 4.737902 |
| 13 | Oeu006130.1 | rna-directed rna polymerase | 4.65852 |
| 14 | Oeu038882.1 | homeobox leucine zipper protein | 4.65852 |
| 15 | Oeu029659.1 | adenine nucleotide alpha hydrolases-domain containing protein kinase | 4.605409 |
| 16 | Oeu004236.1 | abc transporter family protein | 4.568883 |
| 17 | Oeu012917.1 | Uncharacterized protein | 4.492934 |
| 18 | Oeu018708.1 | cytochrome p450 family protein | 4.412763 |
| 19 | Oeu027099.1 | bzip transcription factor family protein | 4.412763 |
| 20 | Oeu052401.1 | Putative uncharacterized protein | 4.412763 |
| 21 | Oeu033762.1 | basic helix-loop-helix family protein | 4.370943 |
| 22 | Oeu063498.1 | gcn5-related n-acetyltransferase family protein | 4.370943 |
| 23 | Oeu053046.1 | abscisic acid receptor pyl1 | 4.34957 |
| 24 | Oeu015404.1 |  | 4.28348 |
| 25 | Oeu042032.1 | di-glucose binding protein with leucine-rich repeat domain | 4.28348 |
| 26 | Oeu033423.1 | constans-like protein co2 | 4.237677 |
| 27 | Oeu018745.1 | wrky transcription factor 70 family protein | 4.190371 |
| 28 | Oeu049462.3 | rna-binding domain ccch-type zinc finger protein | 4.190371 |
| 29 | Oeu012921.2 | Putative uncharacterized protein | 4.178298 |
| 30 | Oeu055036.1 | nucleoside diphosphate kinase | 4.141461 |
| 31 | Oeu021019.1 | dicarboxylate diiron 1 family protein | 4.090835 |
| 32 | Oeu037148.1 |  | 4.090835 |
| 33 | Oeu047324.1 | alpha-dox2 | 4.090835 |
| 34 | Oeu012144.1 | auxin repressed dormancy associated protein | 4.06072 |
| 35 | Oeu007123.1 | serine threonine-protein kinase wnk1 | 4.05607 |
| 36 | Oeu012919.2 | lrr receptor-like kinase family protein | 4.051665 |
| 37 | Oeu011705.1 | hipl2 protein | 4.038368 |
| 38 | Oeu025154.1 | glyoxal oxidase-related family protein | 4.038368 |
| 39 | Oeu007494.1 | Uncharacterized protein (Fragment) | 4.011401 |
| 40 | Oeu021504.1 | chlorophyll a b binding protein | 3.99084 |
| 41 | Oeu013490.1 | gdp-fucose o-fucosyltransferase-like protein | 3.98392 |
| 42 | Oeu018842.1 |  | 3.98392 |
| 43 | Oeu060795.1 |  | 3.98392 |
| 44 | Oeu059685.2 | os06g0140000 protein | 3.938832 |
| 45 | Oeu000296.1 | enox5 | 3.935557 |
| 46 | Oeu007004.1 | trna synthetase class i family protein | 3.927337 |
| 47 | Oeu022457.1 | integrin-linked protein kinase-like protein | 3.927337 |
| 48 | Oeu045998.1 | bidirectional sugar transporter sweet1 | 3.927337 |
| 49 | Oeu059280.1 | Uncharacterized protein | 3.927337 |
| 50 | Oeu053645.1 |  | 3.919069 |
| 51 | Oeu039487.1 | duf4228 domain protein | 3.888343 |
| 52 | Oeu007780.1 | heat shock protein | 3.881533 |
| 53 | Oeu015671.1 | chlorophyllase 2 | 3.868443 |
| 54 | Oeu050406.1 | at3g11660 | 3.868443 |
| 55 | Oeu058293.1 | abc transporter family protein | 3.868443 |
| 56 | Oeu061787.1 | pentatricopeptide repeat-containing protein at2g27610 | 3.868443 |
| 57 | Oeu004716.4 | lrr receptor-like kinase family protein | 3.838069 |
| 58 | Oeu001751.1 | ring-h2 finger protein atl47 | 3.807042 |
| 59 | Oeu040089.1 | outer arm dynein light chain 1 | 3.807042 |
| 60 | Oeu042653.6 |  | 3.807042 |
| 61 | Oeu056845.1 | photosystem ii oxygen-evolving complex protein 2 | 3.807042 |
| 62 | Oeu063200.1 | alpha beta fold hydrolase | 3.807042 |
| 63 | Oeu016411.4 | transmembrane | 3.800056 |
| 64 | Oeu033294.1 | at1g68585 | 3.775334 |
| 65 | Oeu062287.2 | Uncharacterized protein | 3.775334 |
| 66 | Oeu010942.1 | wall-associated kinase family protein | 3.759214 |
| 67 | Oeu032472.1 | squamosa promoter-binding-like protein | 3.742912 |
| 68 | Oeu040290.1 | tonoplast intrinsic protein 12 | 3.742912 |
| 69 | Oeu045089.1 | pentatricopeptide repeat-containing protein at2g17033 | 3.742912 |
| 70 | Oeu059169.1 | wall-associated kinase family protein | 3.742912 |
| 71 | Oeu063183.1 | xyloglucan endotransglucosylase hydrolase | 3.742912 |
| 72 | Oeu008569.1 | v-type proton atpase subunit a | 3.698518 |
| 73 | Oeu042370.1 |  | 3.692871 |
| 74 | Oeu004280.1 | hop-interacting protein thi018 | 3.675798 |
| 75 | Oeu010152.2 | leucine rich repeat receptor like protein clavata2 | 3.675798 |
| 76 | Oeu044279.1 | plant basic secretory protein family protein | 3.661992 |
| 77 | Oeu049723.2 | at4g22290 | 3.652714 |
| 78 | Oeu008518.1 |  | 3.614397 |
| 79 | Oeu001280.1 | gata type zinc finger transcription factor family protein | 3.605409 |
| 80 | Oeu011039.5 | disease resistance protein (tir-nbs class) | 3.605409 |
| 81 | Oeu038417.1 |  | 3.605409 |
| 82 | Oeu039736.1 |  | 3.605409 |
| 83 | Oeu056468.1 | lrr receptor-like kinase family protein | 3.605409 |
| 84 | Oeu060668.1 | 2og-fe oxygenase | 3.605409 |
| 85 | Oeu060905.1 | receptor-like protein 2 | 3.605409 |
| 86 | Oeu001340.1 | mlo-like protein | 3.568883 |
| 87 | Oeu046580.1 | galactokinase family protein | 3.568883 |
| 88 | Oeu031673.1 |  | 3.566034 |
| 89 | Oeu049791.1 | p-loop nucleoside triphosphate hydrolase superfamily protein | 3.547141 |
| 90 | Oeu003785.1 | at4g37080 c7a10_280 | 3.531408 |
| 91 | Oeu020156.3 | abc transporter family protein | 3.531408 |
| 92 | Oeu028423.2 | biotin carboxylase chloroplastic | 3.531408 |
| 93 | Oeu035812.1 | aspartyl protease family protein | 3.531408 |
| 94 | Oeu037161.1 |  | 3.531408 |
| 95 | Oeu041231.1 | duf4408 domain protein | 3.531408 |
| 96 | Oeu022016.1 | catalase | 3.510621 |
| 97 | Oeu032906.1 | btb poz domain plant protein | 3.505873 |
| 98 | Oeu015099.1 | probable inactive receptor kinase at1g48480 | 3.492934 |
| 99 | Oeu042652.1 | beta-galactosidase | 3.492934 |
| 100 | Oeu061143.2 | f-box lrr plant protein | 3.492934 |

**Supplementary Tables 4:** Top 100 up-regulated DEGs in olive cultivar ‘Nabali’ under drought relative to control with at least 2 folds.

| # | **Feature ID** | **Annotations - Description** | **N_d/c** |
| --- | --- | --- | --- |
| 1 | Oeu032705.1 | lemir | 8.212771 |
| 2 | Oeu035127.1 | lipid transfer protein | 7.650892 |
| 3 | Oeu050850.1 |  | 7.601982 |
| 4 | Oeu047443.1 | at4g32480 f8b4_180 | 6.065929 |
| 5 | Oeu001768.1 | udp-glucoronosyl udp-glucosyl transferase family protein | 5.831464 |
| 6 | Oeu007324.1 | chaperone protein 11 | 5.819289 |
| 7 | Oeu040673.1 | lil3 family protein | 5.501431 |
| 8 | Oeu021129.1 | at3g53980 | 5.433833 |
| 9 | Oeu053821.1 | fom-2 family protein | 5.294198 |
| 10 | Oeu030165.3 | potassium efflux antiporter | 5.184574 |
| 11 | Oeu011336.1 | sugar transporter sweet | 5.1461 |
| 12 | Oeu019260.1 | ankyrin repeat family protein | 5.106571 |
| 13 | Oeu064707.1 | c4-dicarboxylate transporter malic acid protein | 5.106571 |
| 14 | Oeu018562.1 | glucosyltransferase family protein | 5.024109 |
| 15 | Oeu011738.1 | gland-specific fatty acyl- reductase | 4.981041 |
| 16 | Oeu025973.1 |  | 4.936646 |
| 17 | Oeu008652.1 | phosphoenolpyruvate carboxykinase | 4.843537 |
| 18 | Oeu022674.1 |  | 4.843537 |
| 19 | Oeu048240.1 | calcium-dependent lipid-binding domain-containing protein | 4.843537 |
| 20 | Oeu025780.1 | duf506 family protein | 4.794627 |
| 21 | Oeu038769.1 | leucoanthocyanidin dioxygenase-like protein | 4.794627 |
| 22 | Oeu025976.1 | c2c2-co-like transcription factor | 4.744001 |
| 23 | Oeu016034.1 | 3-oxo-5-alpha-steroid 4-dehydrogenase family protein | 4.691534 |
| 24 | Oeu018774.1 |  | 4.691534 |
| 25 | Oeu029492.1 | pyrroline-5-carboxylate reductase | 4.691534 |
| 26 | Oeu005760.1 | anaphase promoting complex subunit 2 | 4.637086 |
| 27 | Oeu049168.1 | probable carboxylesterase 16 | 4.637086 |
| 28 | Oeu058164.1 |  | 4.637086 |
| 29 | Oeu052682.1 |  | 4.580503 |
| 30 | Oeu011070.1 | beta-amylase | 4.521609 |
| 31 | Oeu017009.1 |  | 4.521609 |
| 32 | Oeu031511.1 | homolog subfamily c member | 4.521609 |
| 33 | Oeu038774.1 | at5g49480 | 4.521609 |
| 34 | Oeu004091.1 | n2 family protein | 4.460208 |
| 35 | Oeu062722.1 | myb family transcription factor family protein | 4.460208 |
| 36 | Oeu016686.1 | dna-binding protein | 4.396078 |
| 37 | Oeu024578.1 | transcription factor tga7 family protein | 4.396078 |
| 38 | Oeu036500.2 |  | 4.396078 |
| 39 | Oeu041022.2 | myb family transcription factor family protein | 4.396078 |
| 40 | Oeu064246.1 | os01g0773100 protein | 4.396078 |
| 41 | Oeu020824.1 | cysteine proteinase superfamily protein | 4.362911 |
| 42 | Oeu017831.1 | thaliana gn=oxs3 pe=2 sv=1 | 4.354499 |
| 43 | Oeu017036.1 | glycerolipase a1 | 4.328964 |
| 44 | Oeu034809.1 | kinase family protein | 4.328964 |
| 45 | Oeu049376.1 |  | 4.328964 |
| 46 | Oeu060060.1 | heavy-metal-associated domain-containing family protein | 4.328964 |
| 47 | Oeu020223.1 |  | 4.258575 |
| 48 | Oeu031063.1 | mitochondrial transcription termination factor family protein | 4.258575 |
| 49 | Oeu037659.1 | endonuclease exonuclease phosphatase family protein | 4.258575 |
| 50 | Oeu042677.1 | transcription factor hy5 | 4.258575 |
| 51 | Oeu003555.1 | leucine-rich repeat receptor-like protein kinase | 4.258575 |
| 52 | Oeu059049.1 | green flesh protein | 4.222049 |
| 53 | Oeu058077.1 | abc transporter family protein | 4.222049 |
| 54 | Oeu006525.1 | gpi-anchored protein | 4.184574 |
| 55 | Oeu012067.1 | gdsl-motif lipase hydrolase family protein | 4.184574 |
| 56 | Oeu015850.1 | ras-related protein rab7 | 4.184574 |
| 57 | Oeu049551.1 |  | 4.184574 |
| 58 | Oeu055333.1 |  | 4.184574 |
| 59 | Oeu032810.1 |  | 4.1461 |
| 60 | Oeu049085.1 | udp-glucoronosyl udp-glucosyl transferase family protein | 4.1461 |
| 61 | Oeu013758.1 | upf0631 plant-like protein | 4.106571 |
| 62 | Oeu022984.1 | proline-rich family protein | 4.106571 |
| 63 | Oeu024369.3 |  | 4.106571 |
| 64 | Oeu026259.1 | amino acid transporter ant1-like protein | 4.106571 |
| 65 | Oeu036103.2 | trehalose 6-phosphate phosphatase | 4.106571 |
| 66 | Oeu038760.2 | electron transport sco1 family | 4.106571 |
| 67 | Oeu042872.1 |  | 4.106571 |
| 68 | Oeu048076.2 | zinc finger protein 2 | 4.106571 |
| 69 | Oeu051201.1 | wrky transcription factor 6 family protein | 4.106571 |
| 70 | Oeu002365.1 |  | 4.065929 |
| 71 | Oeu040831.1 | duf1645 family protein | 4.065929 |
| 72 | Oeu013814.2 | gdp-fucose protein o-fucosyltransferase | 4.024109 |
| 73 | Oeu016619.1 | triose-phosphate transporter family protein | 4.024109 |
| 74 | Oeu024418.2 | dimethyladenosine transferase | 4.024109 |
| 75 | Oeu029363.1 | mitochondrial substrate carrier family protein | 4.024109 |
| 76 | Oeu033189.2 | squamosa promoter-binding-like protein | 4.024109 |
| 77 | Oeu034335.1 | inner membrane protein oxa1 | 4.024109 |
| 78 | Oeu038878.1 | utp-glucose glucosyltransferase family protein | 4.024109 |
| 79 | Oeu039074.1 |  | 4.024109 |
| 80 | Oeu039926.1 | os01g0613300 protein | 4.024109 |
| 81 | Oeu044523.1 | zinc finger family protein | 4.024109 |
| 82 | Oeu061877.2 | ras-gtpase-activating protein-binding protein | 4.024109 |
| 83 | Oeu029200.1 | non-specific lipid-transfer protein | 4.001602 |
| 84 | Oeu019687.1 | ef hand calcium-binding family protein | 3.99554 |
| 85 | Oeu015664.1 | armadillo beta-catenin repeat family protein | 3.981041 |
| 86 | Oeu036679.1 |  | 3.981041 |
| 87 | Oeu033581.1 |  | 3.976175 |
| 88 | Oeu029201.1 | non-specific lipid-transfer protein | 3.966792 |
| 89 | Oeu017346.1 |  | 3.936646 |
| 90 | Oeu035320.1 | at3g51010 | 3.936646 |
| 91 | Oeu039629.1 | kinase family protein | 3.936646 |
| 92 | Oeu041395.2 | rwp-rk domain-containing family protein | 3.936646 |
| 93 | Oeu042946.1 | dof affecting germination 2 family protein | 3.936646 |
| 94 | Oeu054354.2 | ketopantoate hydroxymethyltransferase family protein | 3.936646 |
| 95 | Oeu006724.1 |  | 3.936646 |
| 96 | Oeu017967.1 | structural constituent of cell wall | 3.936646 |
| 97 | Oeu021628.1 | monothiol glutaredoxin-s2 protein | 3.936646 |
| 98 | Oeu033468.1 | at1g80160 protein | 3.936646 |
| 99 | Oeu014177.1 |  | 3.871551 |
| 100 | Oeu032972.1 |  | 3.859479 |

**Supplementary Tables 5:** Top 100 up-regulated DEGs in olive cultivar ‘Nabali’ under salinity relative to control with at least 2 folds.

| # | **Feature ID** | **Annotations - Description** | **N_s/c** |
| --- | --- | --- | --- |
| 1 | Oeu012604.1 | xyloglucan endotransglucosylase hydrolase | 7.037577 |
| 2 | Oeu035401.1 | at1g36320 f7f23_4 | 6.775836 |
| 3 | Oeu024369.3 |  | 6.696109 |
| 4 | Oeu060006.1 | ndh-dependent cyclic electron flow | 6.585405 |
| 5 | Oeu017977.1 | gdsl-motif lipase hydrolase family protein | 6.470297 |
| 6 | Oeu007444.1 | 3-ketoacyl- synthase | 6.406602 |
| 7 | Oeu031063.1 | mitochondrial transcription termination factor family protein | 6.406602 |
| 8 | Oeu011199.1 | gland-specific fatty acyl- reductase | 6.291959 |
| 9 | Oeu044305.1 | u-box domain-containing family protein | 6.190873 |
| 10 | Oeu057164.1 | Uncharacterized protein | 6.167414 |
| 11 | Oeu040536.1 | phytosulfokine peptide | 6.082167 |
| 12 | Oeu009283.1 |  | 6.069567 |
| 13 | Oeu039204.1 | protein exordium-like 3 | 6.004861 |
| 14 | Oeu063550.2 | hva22-like protein | 5.950923 |
| 15 | Oeu016926.1 | eukaryotic aspartyl protease family protein | 5.950923 |
| 16 | Oeu016664.1 | ferric reductase-like transmembrane component family protein | 5.937117 |
| 17 | Oeu042318.2 | fatty acid amide hydrolase-like protein | 5.923178 |
| 18 | Oeu004303.1 | eukaryotic aspartyl protease family protein | 5.909102 |
| 19 | Oeu006724.1 |  | 5.894888 |
| 20 | Oeu053624.1 | lim transcription factor | 5.864211 |
| 21 | Oeu017776.1 | auxin canalization protein | 5.806533 |
| 22 | Oeu017967.1 | structural constituent of cell wall | 5.775836 |
| 23 | Oeu013592.1 | gdp-fucose protein o-fucosyltransferase | 5.72853 |
| 24 | Oeu002507.1 | beta-galactosidase | 5.72853 |
| 25 | Oeu016821.1 | rho gdp-dissociation inhibitor 1 family protein | 5.71241 |
| 26 | Oeu027587.1 | protein phosphatase 2c family protein | 5.702651 |
| 27 | Oeu001186.1 | abc transporter b family-like protein | 5.696109 |
| 28 | Oeu024076.1 | major intrinsic protein family transporter | 5.646068 |
| 29 | Oeu036500.2 |  | 5.646068 |
| 30 | Oeu049432.1 |  | 5.646068 |
| 31 | Oeu001142.1 | phototropic-responsive nph3 family protein | 5.611716 |
| 32 | Oeu034335.1 | inner membrane protein oxa1 | 5.611716 |
| 33 | Oeu004213.1 | calcium-binding ef hand family protein | 5.594229 |
| 34 | Oeu027672.1 | lrr receptor-like kinase | 5.576527 |
| 35 | Oeu027266.1 | zip zinc iron transport family protein | 5.564604 |
| 36 | Oeu015177.1 | alpha beta hydrolase family protein | 5.558605 |
| 37 | Oeu055877.2 | signal peptidase i | 5.552581 |
| 38 | Oeu034007.1 |  | 5.522079 |
| 39 | Oeu049015.1 |  | 5.503464 |
| 40 | Oeu060351.1 | ppr containing plant-like protein | 5.503464 |
| 41 | Oeu000251.1 | phosphate-responsive family protein | 5.484604 |
| 42 | Oeu012394.1 | homeobox associated leucine zipper protein | 5.483779 |
| 43 | Oeu048731.1 | at5g62720 | 5.436349 |
| 44 | Oeu034296.1 | basic helix-loop-helix family protein | 5.426502 |
| 45 | Oeu062996.1 | duf789 family protein | 5.426502 |
| 46 | Oeu022175.1 |  | 5.426502 |
| 47 | Oeu033762.1 | basic helix-loop-helix family protein | 5.416586 |
| 48 | Oeu032972.1 |  | 5.413266 |
| 49 | Oeu032948.1 | nadh-cytochrome b5 reductase | 5.406602 |
| 50 | Oeu042370.1 |  | 5.406602 |
| 51 | Oeu001139.1 | zinc finger family protein | 5.386424 |
| 52 | Oeu017675.1 | epoxide hydrolase | 5.386424 |
| 53 | Oeu024323.1 | myb transcription factor myb109 | 5.386424 |
| 54 | Oeu040652.1 | xyloglucan endotransglucosylase hydrolase | 5.386424 |
| 55 | Oeu060260.1 | cyp77a3p family protein | 5.36596 |
| 56 | Oeu063625.1 | 4-diphosphocytidyl-2-c-methyl-d-erythritol chloroplastic chromoplastic | 5.355618 |
| 57 | Oeu000296.1 | enox5 | 5.349377 |
| 58 | Oeu023038.1 | two-component response regulator arr3-like protein | 5.345201 |
| 59 | Oeu057084.1 |  | 5.345201 |
| 60 | Oeu013113.1 | hop-interacting protein thi016 | 5.32414 |
| 61 | Oeu055277.1 | calcineurin subunit b | 5.32414 |
| 62 | Oeu031821.1 | udp-glucosyltransferase family protein | 5.281071 |
| 63 | Oeu004998.1 | thylakoid lumenal kda protein | 5.273766 |
| 64 | Oeu044001.1 | rna-directed rna polymerase | 5.2701 |
| 65 | Oeu019122.1 | axi 1 protein | 5.259045 |
| 66 | Oeu023795.1 | myb transcription factor myb109 | 5.259045 |
| 67 | Oeu046681.1 | plant invertase pectin methylesterase inhibitor | 5.259045 |
| 68 | Oeu012144.1 | auxin repressed dormancy associated protein | 5.245106 |
| 69 | Oeu031371.1 | protein da1-related 2 | 5.236677 |
| 70 | Oeu044257.1 | upf0235 protein at5g63440 | 5.236677 |
| 71 | Oeu038384.1 | alpha-galactosidase | 5.213957 |
| 72 | Oeu055333.1 |  | 5.213957 |
| 73 | Oeu040289.1 | tonoplast intrinsic protein 12 | 5.207399 |
| 74 | Oeu020628.1 | os02g0557300 protein | 5.198609 |
| 75 | Oeu037677.1 | os05g0395300 protein | 5.190873 |
| 76 | Oeu040178.1 | tyrosine kinase family protein | 5.190873 |
| 77 | Oeu011933.4 | #NAME? | 5.181535 |
| 78 | Oeu058077.1 | abc transporter family protein | 5.179191 |
| 79 | Oeu015982.1 | os05g0218400 protein | 5.167414 |
| 80 | Oeu032393.2 | Uncharacterized protein | 5.167414 |
| 81 | Oeu034160.1 | Uncharacterized protein | 5.167414 |
| 82 | Oeu037357.1 | cytochrome p450 family protein | 5.167414 |
| 83 | Oeu049917.2 | peptidyl-prolyl cis-trans isomerase | 5.167414 |
| 84 | Oeu003596.1 | auxin-responsive protein | 5.161489 |
| 85 | Oeu043177.1 | mitochondrial adenine nucleotide transporter adnt1 | 5.159509 |
| 86 | Oeu012067.1 | gdsl-motif lipase hydrolase family protein | 5.143568 |
| 87 | Oeu014913.1 | at3g57730 f15b8_80 | 5.143568 |
| 88 | Oeu043744.1 |  | 5.143568 |
| 89 | Oeu045977.1 | staygreen protein | 5.143568 |
| 90 | Oeu032589.1 | n-carbamyl-l-amino acid hydrolase family protein | 5.13553 |
| 91 | Oeu035625.1 | o-acyltransferase wsd1-like protein | 5.11932 |
| 92 | Oeu046174.1 | 3-oxo-5-alpha-steroid 4-dehydrogenase | 5.111146 |
| 93 | Oeu003555.1 | leucine-rich repeat receptor-like protein kinase | 5.107042 |
| 94 | Oeu034307.1 |  | 5.097419 |
| 95 | Oeu008416.1 | glutamyl-trna reductase | 5.091545 |
| 96 | Oeu001983.1 |  | 5.069567 |
| 97 | Oeu012150.1 |  | 5.069567 |
| 98 | Oeu012921.2 | Putative uncharacterized protein | 5.069567 |
| 99 | Oeu037360.1 | mediator of rna polymerase ii transcription subunit 21 | 5.069567 |
| 100 | Oeu039629.1 | kinase family protein | 5.069567 |

**Supplementary Tables 6:** Top 100 up-regulated DEGs in olive cultivar ‘Manzanillo’ under drought relative to control with at least 2 folds.

| # | **Feature ID** | **Annotations - Description** | **Z_d/c** |
| --- | --- | --- | --- |
| 1 | Oeu029201.1 | non-specific lipid-transfer protein | 8.843611 |
| 2 | Oeu015612.1 | allene oxide synthase family protein | 6.518777 |
| 3 | Oeu017720.1 | u-box domain-containing family protein | 6.453324 |
| 4 | Oeu037159.1 | avr9 cf-9 rapidly elicited protein | 6.438025 |
| 5 | Oeu001701.1 | glycosyl transferase family 17 family protein | 6.397471 |
| 6 | Oeu030131.1 | c-repeat binding factor | 6.097567 |
| 7 | Oeu026920.1 | harpin-induced 1 | 5.999209 |
| 8 | Oeu036891.1 | heavy metal transport detoxification superfamily protein | 5.961186 |
| 9 | Oeu013901.1 | rap2-like protein | 5.943959 |
| 10 | Oeu036417.1 | mlo-like protein | 5.930903 |
| 11 | Oeu004155.1 | mitogen-activated protein kinase | 5.928715 |
| 12 | Oeu009320.2 | alcohol dehydroge family protein | 5.734506 |
| 13 | Oeu013711.1 | ap2 erf domain transcription factor | 5.659978 |
| 14 | Oeu036202.3 | plant inositol phosphorylceramide synthase | 5.593491 |
| 15 | Oeu016033.1 |  | 5.573009 |
| 16 | Oeu048286.1 | phosphatase 2c family protein | 5.350617 |
| 17 | Oeu009292.1 |  | 5.32347 |
| 18 | Oeu029200.1 | non-specific lipid-transfer protein | 5.319468 |
| 19 | Oeu043954.1 | endochitinase 2 family protein | 5.306048 |
| 20 | Oeu012851.3 | mitochondrial substrate carrier family protein | 5.251081 |
| 21 | Oeu035127.1 | lipid transfer protein | 5.251081 |
| 22 | Oeu032703.1 | at2g31130 | 5.237006 |
| 23 | Oeu029527.5 | 5-methylthioribose kinase family protein | 5.215632 |
| 24 | Oeu030171.1 | atpase 6 family protein | 5.208437 |
| 25 | Oeu057980.1 | vhs domain-containing family protein | 5.201205 |
| 26 | Oeu029830.1 | tubby-like f-box protein | 5.186633 |
| 27 | Oeu051201.1 | wrky transcription factor 6 family protein | 5.171911 |
| 28 | Oeu059662.3 |  | 5.164494 |
| 29 | Oeu050986.2 | #NAME? | 5.149543 |
| 30 | Oeu031282.1 |  | 5.124914 |
| 31 | Oeu036033.1 | heavy metal-associated domain protein | 5.121454 |
| 32 | Oeu033871.1 | carboxypeptidase | 5.111475 |
| 33 | Oeu055645.1 | calcium-binding ef hand family protein | 5.111475 |
| 34 | Oeu048965.1 |  | 5.088143 |
| 35 | Oeu037955.2 | ca2+ h+ exchanger family protein | 5.064427 |
| 36 | Oeu021710.2 | adenylate kinase family protein | 5.024012 |
| 37 | Oeu018813.2 | core-2 i-branching enzyme | 4.982433 |
| 38 | Oeu030173.1 | glycoside hydrolase family 18 protein | 4.961186 |
| 39 | Oeu006434.2 | two-component response regulator arr2-like protein | 4.948285 |
| 40 | Oeu017475.1 | c2 domain-containing family protein | 4.92433 |
| 41 | Oeu032620.1 | 3-ketoacyl- synthase | 4.913309 |
| 42 | Oeu001838.1 | copper ion transmembrane transporter | 4.904431 |
| 43 | Oeu026398.1 |  | 4.899971 |
| 44 | Oeu020732.1 | ubiquitin-protein ligase | 4.895498 |
| 45 | Oeu016606.1 | chitinase | 4.87292 |
| 46 | Oeu036587.1 | fatty acid desaturase cytochrome b5 fusion protein mrna | 4.871402 |
| 47 | Oeu016272.1 |  | 4.868362 |
| 48 | Oeu026991.1 | protein | 4.859202 |
| 49 | Oeu057044.1 | gras family transcription factor | 4.859202 |
| 50 | Oeu038934.1 | mitogen-activated protein kinase | 4.858052 |
| 51 | Oeu041125.1 | fad-binding berberine family protein | 4.846897 |
| 52 | Oeu039387.1 | duf3133 family protein | 4.840705 |
| 53 | Oeu044912.1 | plant cell wall protein 88 | 4.840705 |
| 54 | Oeu055845.1 | transferase family protein | 4.840705 |
| 55 | Oeu029571.1 | zinc finger family protein | 4.812508 |
| 56 | Oeu010504.1 | glutamyl-trna reductase | 4.810133 |
| 57 | Oeu005799.2 | transketolase family protein | 4.793399 |
| 58 | Oeu028648.1 | u-box domain-containing family protein | 4.783749 |
| 59 | Oeu030010.1 | protein-tyrosine-phosphatase | 4.74449 |
| 60 | Oeu038774.1 | at5g49480 | 4.729488 |
| 61 | Oeu023687.1 |  | 4.722696 |
| 62 | Oeu041817.1 |  | 4.714328 |
| 63 | Oeu006984.1 | ap47 50p mrna family protein | 4.693864 |
| 64 | Oeu016353.1 | nucleobase-ascorbate transporter-like protein | 4.693864 |
| 65 | Oeu029478.1 |  | 4.688702 |
| 66 | Oeu057643.2 | wrky family transcription factor family protein | 4.669616 |
| 67 | Oeu046773.1 | epsilon-adaptin family protein | 4.652044 |
| 68 | Oeu028662.1 | heat shock transcription factor family protein | 4.63067 |
| 69 | Oeu055337.3 |  | 4.619863 |
| 70 | Oeu003793.1 | pq-loop repeat family protein | 4.608975 |
| 71 | Oeu006348.1 | avr9 cf-9 rapidly elicited protein | 4.608975 |
| 72 | Oeu025973.1 |  | 4.608975 |
| 73 | Oeu033501.1 | pyridoxin biosynthesis pdx1-like protein 2 | 4.608975 |
| 74 | Oeu038985.1 | udp-glucoronosyl udp-glucosyl transferase family protein | 4.608975 |
| 75 | Oeu057169.1 | glutathione s-transferase 30 family protein | 4.598004 |
| 76 | Oeu055812.1 | os09g0281800 protein | 4.592487 |
| 77 | Oeu022909.3 | histidinol dehydrogenase family protein | 4.586949 |
| 78 | Oeu026339.1 |  | 4.575808 |
| 79 | Oeu003384.3 | atp-dependent dna helicase family protein | 4.564581 |
| 80 | Oeu011155.1 | auxin-responsive gh3 family protein | 4.541861 |
| 81 | Oeu013712.1 | cytochrome p450 family protein | 4.541861 |
| 82 | Oeu040105.1 | glycosyl hydrolase family 17 family protein | 4.541861 |
| 83 | Oeu054601.1 | violaxanthin de- chloroplastic | 4.530365 |
| 84 | Oeu030025.1 | f-box protein interaction domain protein | 4.518777 |
| 85 | Oeu059199.1 | mlh1 | 4.518777 |
| 86 | Oeu051277.1 | peroxidase | 4.512948 |
| 87 | Oeu061927.1 | rna polymerase ii subunit b1 ctd phosphatase | 4.512948 |
| 88 | Oeu042883.1 | gras2 | 4.511779 |
| 89 | Oeu063286.1 | nodulin family protein | 4.509439 |
| 90 | Oeu019926.1 | indole-3-acetate beta-d-glucosyltransferase family protein | 4.507095 |
| 91 | Oeu032888.1 | zinc finger family protein | 4.507095 |
| 92 | Oeu035434.1 | n-carbamoylputrescine amidase | 4.507095 |
| 93 | Oeu064510.1 |  | 4.507095 |
| 94 | Oeu032705.1 | lemir | 4.495318 |
| 95 | Oeu002227.1 |  | 4.49223 |
| 96 | Oeu045926.1 |  | 4.487413 |
| 97 | Oeu007998.4 | os06g0687800 protein | 4.483444 |
| 98 | Oeu029108.1 | adenylate kinase chloroplastic | 4.483444 |
| 99 | Oeu054834.1 | glycylpeptide n-tetradecanoyltransferase | 4.483444 |
| 100 | Oeu007288.1 | ap2 erf domain transcription factor | 4.480033 |

**Supplementary Tables 7:** Top 100 up-regulated DEGs in olive cultivar ‘Manzanillo’ under salinity relative to control with at least 2 folds.

| # | **Feature ID** | **Annotations - Description** | **Z_s/c** |
| --- | --- | --- | --- |
| 1 | Oeu012144.1 | auxin repressed dormancy associated protein | 6.848955 |
| 2 | Oeu038414.1 |  | 6.659268 |
| 3 | Oeu007780.1 | heat shock protein | 6.56634 |
| 4 | Oeu059302.1 |  | 5.862442 |
| 5 | Oeu029201.1 | non-specific lipid-transfer protein | 5.393504 |
| 6 | Oeu062259.1 | universal stress family protein | 5.256 |
| 7 | Oeu019681.1 | 60s ribosomal protein l4-1 | 5.187053 |
| 8 | Oeu021046.1 | glutaredoxin family protein | 5.146122 |
| 9 | Oeu058049.1 |  | 5.049549 |
| 10 | Oeu023193.1 |  | 5.015866 |
| 11 | Oeu051807.1 |  | 4.946045 |
| 12 | Oeu063286.1 | nodulin family protein | 4.912268 |
| 13 | Oeu050429.1 | lysine and histidine specific transporter family protein | 4.885162 |
| 14 | Oeu025980.1 | at4g05030 protein | 4.872671 |
| 15 | Oeu047138.1 | pathogenesis-related protein bet v i family protein | 4.85373 |
| 16 | Oeu047187.1 | glycosyl hydrolase family protein | 4.741427 |
| 17 | Oeu050155.1 | at5g13890 | 4.741427 |
| 18 | Oeu030408.1 | atp-dependent protease la domain-containing family protein | 4.713682 |
| 19 | Oeu052072.1 |  | 4.713682 |
| 20 | Oeu062474.1 | plant u-box protein | 4.699607 |
| 21 | Oeu000833.2 | os03g0123600 protein | 4.671038 |
| 22 | Oeu007848.1 | cmp-sialic acid | 4.612144 |
| 23 | Oeu034304.1 |  | 4.558563 |
| 24 | Oeu035434.1 | n-carbamoylputrescine amidase | 4.470125 |
| 25 | Oeu047572.1 | pra1 family protein | 4.470125 |
| 26 | Oeu055483.1 | at2g46890 | 4.453446 |
| 27 | Oeu003154.1 |  | 4.436572 |
| 28 | Oeu058837.1 | adp-ribosylation factor-like protein a1d | 4.384733 |
| 29 | Oeu036990.1 |  | 4.37591 |
| 30 | Oeu001838.1 | copper ion transmembrane transporter | 4.367031 |
| 31 | Oeu032272.1 | stem 28 kda glycoprotein | 4.312584 |
| 32 | Oeu037599.1 | duf3456 domain protein | 4.312584 |
| 33 | Oeu044304.1 | caffeoyl- o-methyltransferase family protein | 4.305166 |
| 34 | Oeu064799.1 | dna-binding family protein | 4.293968 |
| 35 | Oeu049322.1 |  | 4.275109 |
| 36 | Oeu010263.1 | transferring glycosyl group transferase | 4.24635 |
| 37 | Oeu007924.1 | cyclin d3-2 family protein | 4.236635 |
| 38 | Oeu012990.1 | Putative uncharacterized protein | 4.236635 |
| 39 | Oeu025658.1 | lob domain protein 39 | 4.217006 |
| 40 | Oeu036609.1 | cytochrome p450 family protein | 4.217006 |
| 41 | Oeu050146.1 | serine threonine-protein kinase | 4.217006 |
| 42 | Oeu058204.1 | 3-beta-hydroxysteroid-delta family protein | 4.217006 |
| 43 | Oeu056727.1 | #NAME? | 4.20377 |
| 44 | Oeu063417.1 | enox5 | 4.192088 |
| 45 | Oeu050099.1 | zinc finger family protein | 4.176929 |
| 46 | Oeu050390.1 | mate efflux family protein | 4.176929 |
| 47 | Oeu000296.1 | enox5 | 4.156464 |
| 48 | Oeu024074.1 | major intrinsic protein family transporter | 4.156464 |
| 49 | Oeu047963.2 | iron ion binding oxidoreductase | 4.156464 |
| 50 | Oeu054685.1 | probable lrr receptor-like serine threonine-protein kinase at4g08850 | 4.156464 |
| 51 | Oeu009949.1 | transmembrane | 4.135706 |
| 52 | Oeu020121.1 | plant f10m23-360 protein | 4.135706 |
| 53 | Oeu050593.1 | serine threonine-protein kinase | 4.135706 |
| 54 | Oeu011909.1 | vesicle-associated protein 43832 | 4.114644 |
| 55 | Oeu036514.1 | 60s ribosomal protein l27a-3 | 4.103997 |
| 56 | Oeu021710.2 | adenylate kinase family protein | 4.093271 |
| 57 | Oeu055967.1 | zinc finger bed domain-containing protein daysleeper | 4.093271 |
| 58 | Oeu033048.1 | phospho-2-dehydro-3-deoxyheptonate aldolase chloroplastic | 4.071576 |
| 59 | Oeu047573.1 | atp synthase delta chain family protein | 4.071576 |
| 60 | Oeu034303.1 |  | 4.068046 |
| 61 | Oeu020212.1 | 5-methyltetrahydropteroyltriglutamate--homocysteine methyltransferase family protein | 4.049549 |
| 62 | Oeu029922.1 | gcn5-related n-acetyltransferase family protein | 4.049549 |
| 63 | Oeu060435.2 | transmembrane | 4.049549 |
| 64 | Oeu061772.2 | udp-glucose:flavonoid 3-o-glucosyltransferase | 4.049549 |
| 65 | Oeu020232.1 | f-box family protein | 4.027181 |
| 66 | Oeu053624.1 | lim transcription factor | 4.027181 |
| 67 | Oeu019846.1 | plant f25p12-18 protein | 4.027181 |
| 68 | Oeu005137.1 | cold shock domain-containing protein 3 | 4.004461 |
| 69 | Oeu010566.1 |  | 4.004461 |
| 70 | Oeu036669.1 | transmembrane 9 superfamily member 1 | 4.004461 |
| 71 | Oeu039881.1 |  | 4.004461 |
| 72 | Oeu045926.1 |  | 3.996808 |
| 73 | Oeu016033.1 |  | 3.992966 |
| 74 | Oeu026920.1 | harpin-induced 1 | 3.992966 |
| 75 | Oeu061668.1 | nadh-ubiquinone oxidoreductase family protein | 3.992966 |
| 76 | Oeu021625.1 | casp-like protein | 3.981378 |
| 77 | Oeu058171.1 | at1g61730 protein | 3.981378 |
| 78 | Oeu029036.1 |  | 3.981378 |
| 79 | Oeu035917.1 | subtilase family protein | 3.981378 |
| 80 | Oeu053195.1 |  | 3.957919 |
| 81 | Oeu057201.1 | cytochrome p450 family protein | 3.957919 |
| 82 | Oeu009320.2 | alcohol dehydroge family protein | 3.934072 |
| 83 | Oeu018777.1 |  | 3.929255 |
| 84 | Oeu018774.1 |  | 3.909824 |
| 85 | Oeu030010.1 | protein-tyrosine-phosphatase | 3.909824 |
| 86 | Oeu053734.1 |  | 3.909824 |
| 87 | Oeu036356.1 | zinc finger family protein | 3.900411 |
| 88 | Oeu051185.1 | transferring glycosyl group transferase | 3.897546 |
| 89 | Oeu007836.2 |  | 3.890679 |
| 90 | Oeu047135.1 | pathogenesis-related protein bet v i family protein | 3.887924 |
| 91 | Oeu025624.1 | dna-binding family protein | 3.885162 |
| 92 | Oeu027202.1 |  | 3.885162 |
| 93 | Oeu035457.1 | histone-lysine n-methyltransferase | 3.872671 |
| 94 | Oeu038214.1 | telomerase cajal body-like protein | 3.860071 |
| 95 | Oeu043983.1 | heat shock factor protein 5 | 3.860071 |
| 96 | Oeu053212.1 | at4g08940 | 3.860071 |
| 97 | Oeu013066.1 |  | 3.834536 |
| 98 | Oeu027686.1 | nucleotidyltransferase | 3.834536 |
| 99 | Oeu034320.1 | udp-d-glucuronate 4-epimerase | 3.834536 |
| 100 | Oeu061779.1 | hippocampus abundant transcript-like protein | 3.834536 |

**Supplementary Tables 8:** Top 100 up-regulated DEGs in olive cultivar ‘Mehras’ under drought relative to control with at least 2 folds.

| # | **Feature ID** | **Annotations - Description** | **M_d/c** |
| --- | --- | --- | --- |
| 1 | Oeu043352.1 | atp-dependent clp protease proteolytic subunit | 5.229572 |
| 2 | Oeu021363.2 | zinc finger family protein | 5.094642 |
| 3 | Oeu032705.1 | lemir | 5.051574 |
| 4 | Oeu003335.2 | wd g-beta repeat protein | 5.02213 |
| 5 | Oeu035711.1 | basic helix-loop-helix family protein | 4.945779 |
| 6 | Oeu048804.2 |  | 4.670144 |
| 7 | Oeu041787.3 | maternal effect embryo arrest 18 protein | 4.651036 |
| 8 | Oeu039015.1 | poly rna polymerase gld2-like protein | 4.63167 |
| 9 | Oeu028109.1 | aspartate kinase family protein | 4.592142 |
| 10 | Oeu025739.1 |  | 4.571964 |
| 11 | Oeu026381.1 | like protein | 4.571964 |
| 12 | Oeu054785.1 | f-box family protein | 4.571964 |
| 13 | Oeu036090.1 | tcp family transcription factor | 4.561768 |
| 14 | Oeu013701.3 | b3 domain-containing protein os01g0234100 | 4.5515 |
| 15 | Oeu019370.1 | diacylglycerol kinase | 4.541158 |
| 16 | Oeu014567.1 | alpha-mannosidase | 4.50968 |
| 17 | Oeu016029.1 | hexosyltransferase | 4.50968 |
| 18 | Oeu005905.1 |  | 4.477499 |
| 19 | Oeu005601.1 | phd finger and bromo-adjacent homology domain-containing protein | 4.466611 |
| 20 | Oeu007289.1 |  | 4.466611 |
| 21 | Oeu018431.1 | pollen-specific sf21-like protein | 4.466611 |
| 22 | Oeu025037.1 | phospholipid glycerol acyltransferase family protein | 4.466611 |
| 23 | Oeu043314.1 | aaa-type atpase family protein | 4.466611 |
| 24 | Oeu047202.2 | os05g0443700 protein | 4.466611 |
| 25 | Oeu019706.1 | at1g55840 f14j16_2 | 4.444585 |
| 26 | Oeu033638.1 | map kinase | 4.410902 |
| 27 | Oeu048323.1 | sucrose synthase | 4.410902 |
| 28 | Oeu000748.2 |  | 4.399497 |
| 29 | Oeu007427.1 | auxin response factor | 4.399497 |
| 30 | Oeu043073.2 | dead-box atp-dependent rna helicase | 4.399497 |
| 31 | Oeu010556.1 | transducin family protein wd-40 repeat family protein | 4.399497 |
| 32 | Oeu013289.1 | casein kinase ii subunit chloroplastic | 4.399497 |
| 33 | Oeu039596.1 | serine threonine-protein kinase | 4.399497 |
| 34 | Oeu048370.1 | octicosapeptide phox bem1p domain-containing family protein | 4.352954 |
| 35 | Oeu014023.1 | rna pseudouridine | 4.329108 |
| 36 | Oeu021967.1 | mate efflux family protein | 4.329108 |
| 37 | Oeu060883.1 | duf506 family protein | 4.329108 |
| 38 | Oeu035436.1 | rac gtpase activating protein 1 | 4.30486 |
| 39 | Oeu037103.2 | spermine spermidine synthase | 4.30486 |
| 40 | Oeu040176.1 | ubiquitin-protein | 4.30486 |
| 41 | Oeu041039.1 | calcium-transporting atpase | 4.30486 |
| 42 | Oeu051802.2 | ap2 domain-containing transcription factor family protein | 4.30486 |
| 43 | Oeu001990.2 | vacuolar protein sorting-associated protein 9a | 4.292582 |
| 44 | Oeu049301.1 | myb-like dna-binding shaqkyf class protein | 4.280198 |
| 45 | Oeu018758.1 | kunitz-type protease inhibitor kpi- | 4.273165 |
| 46 | Oeu031181.1 | nucleic acid binding family protein | 4.255107 |
| 47 | Oeu057901.1 |  | 4.255107 |
| 48 | Oeu019002.1 | n-acetylglutamate kinase | 4.242396 |
| 49 | Oeu001264.1 | peptide upstream orf | 4.229572 |
| 50 | Oeu022034.1 | duf538 family protein | 4.229572 |
| 51 | Oeu024688.1 | ring-finger ubiquitin ligase | 4.203577 |
| 52 | Oeu046414.1 | monocopper oxidase family protein | 4.177104 |
| 53 | Oeu036504.1 | octicosapeptide phox bem1p domain-containing family protein | 4.150137 |
| 54 | Oeu038127.1 | wall-associated kinase family protein | 4.150137 |
| 55 | Oeu052794.1 | lutescent 2 | 4.150137 |
| 56 | Oeu062820.1 | inositol-tetrakisphosphate 1-kinase | 4.150137 |
| 57 | Oeu012190.1 | eukaryotic translation initiation factor 3 subunit l | 4.141035 |
| 58 | Oeu014699.1 | double-stranded rna-binding motif protein | 4.136462 |
| 59 | Oeu004544.1 | multiple chloroplast division site 1 | 4.122657 |
| 60 | Oeu013718.1 | endonuclease or glycosyl hydrolase | 4.122657 |
| 61 | Oeu015968.1 | mitochondrial rho gtpase | 4.122657 |
| 62 | Oeu017474.1 | 3 2 proteins | 4.122657 |
| 63 | Oeu025604.1 | at4g32605 protein | 4.122657 |
| 64 | Oeu044521.1 | ulp1 protease family protein | 4.094642 |
| 65 | Oeu064326.1 | glycoside hydrolase family 28 family protein | 4.094642 |
| 66 | Oeu035416.2 | abc transporter family protein | 4.080428 |
| 67 | Oeu012046.1 | cytochrome p450 family protein | 4.066073 |
| 68 | Oeu031295.1 | f-box family protein | 4.066073 |
| 69 | Oeu049419.1 | e3 ubiquitin protein ligase rie1-like | 4.066073 |
| 70 | Oeu054687.1 | phosphoglucan phosphatase chloroplastic | 4.066073 |
| 71 | Oeu056403.1 | zinc-binding dehydrogenase family oxidoreductase | 4.066073 |
| 72 | Oeu064524.1 | mads-box transcription factor family protein | 4.066073 |
| 73 | Oeu017646.1 | nudix family protein | 4.051574 |
| 74 | Oeu001823.1 | Uncharacterized protein | 4.036927 |
| 75 | Oeu019435.1 |  | 4.036927 |
| 76 | Oeu024061.1 | os08g0248900 protein | 4.036927 |
| 77 | Oeu049685.1 | protein yipf | 4.036927 |
| 78 | Oeu002119.1 | alanine:glyoxylate aminotransferase 2 family protein | 4.007179 |
| 79 | Oeu004654.1 | ice-like protease p20 domain protein | 4.007179 |
| 80 | Oeu021046.1 | glutaredoxin family protein | 4.007179 |
| 81 | Oeu051175.1 | inositol polyphosphate multikinase beta | 4.007179 |
| 82 | Oeu055744.1 | coatomer subunit alpha | 3.992073 |
| 83 | Oeu051333.1 | myb transcription factor family protein | 3.976806 |
| 84 | Oeu057518.1 | pentatricopeptide repeat-containing family protein | 3.976806 |
| 85 | Oeu007666.2 | c-4 methyl sterol oxidase | 3.976806 |
| 86 | Oeu026361.1 | serine threonine-protein phosphatase bsl2 | 3.976806 |
| 87 | Oeu035746.1 |  | 3.976806 |
| 88 | Oeu048384.2 | 2og-fe oxygenase family oxidoreductase | 3.976806 |
| 89 | Oeu057196.3 |  | 3.976806 |
| 90 | Oeu022016.1 | catalase | 3.967181 |
| 91 | Oeu006500.1 | zinc finger family protein | 3.945779 |
| 92 | Oeu011664.1 | gpi transamidase subunit pig-u | 3.945779 |
| 93 | Oeu015671.1 | chlorophyllase 2 | 3.945779 |
| 94 | Oeu023081.1 | ramosa 1 enhancer locus 2 | 3.945779 |
| 95 | Oeu032975.1 |  | 3.945779 |
| 96 | Oeu002489.1 |  | 3.91407 |
| 97 | Oeu003108.1 | dna repair protein rad23 | 3.91407 |
| 98 | Oeu010754.1 | rna recognition motif | 3.91407 |
| 99 | Oeu047310.1 | affected traffi cking protein | 3.91407 |
| 100 | Oeu050053.1 | trypsin-like serine and cysteine proteases | 3.91407 |

**Supplementary Tables 9:** Top 100 up-regulated DEGs in olive cultivar ‘Mehras’ under salinity relative to control with at least 2 folds.

| # | **Feature ID** | **Annotations - Description** | **M_s/c** |
| --- | --- | --- | --- |
| 1 | Oeu052645.1 | ankyrin repeat family protein | 7.040075 |
| 2 | Oeu040651.1 | xyloglucan endotransglucosylase hydrolase | 6.922606 |
| 3 | Oeu044952.2 | peptide nitrate transporter plant | 6.748355 |
| 4 | Oeu024323.1 | myb transcription factor myb109 | 6.668184 |
| 5 | Oeu057521.1 | iron transporter-related family protein | 6.625922 |
| 6 | Oeu000918.2 | receptor-like protein kinase feronia | 6.546396 |
| 7 | Oeu039015.1 | poly rna polymerase gld2-like protein | 6.51236 |
| 8 | Oeu007811.1 | duf1005 family protein | 6.429672 |
| 9 | Oeu005093.1 | probable methyltransferase pmt8 | 6.282614 |
| 10 | Oeu054785.1 | f-box family protein | 6.220725 |
| 11 | Oeu040652.1 | xyloglucan endotransglucosylase hydrolase | 6.182758 |
| 12 | Oeu014567.1 | alpha-mannosidase | 6.163392 |
| 13 | Oeu053681.1 | abc transporter b family protein | 6.163392 |
| 14 | Oeu012394.1 | homeobox associated leucine zipper protein | 6.155246 |
| 15 | Oeu050429.1 | lysine and histidine specific transporter family protein | 6.125533 |
| 16 | Oeu010839.1 | leucine-rich receptor-like kinase family protein | 6.088365 |
| 17 | Oeu043352.1 | atp-dependent clp protease proteolytic subunit | 6.020028 |
| 18 | Oeu001826.2 |  | 6.014635 |
| 19 | Oeu004641.1 | receptor-like kinase plant | 6.014635 |
| 20 | Oeu021287.1 |  | 5.992858 |
| 21 | Oeu033762.1 | basic helix-loop-helix family protein | 5.992858 |
| 22 | Oeu022991.1 | nitrite transporter family protein | 5.961166 |
| 23 | Oeu010517.3 | oligopeptide transporter 4 | 5.948292 |
| 24 | Oeu003154.1 |  | 5.942624 |
| 25 | Oeu019607.1 | rna recognition motif-containing family protein | 5.887629 |
| 26 | Oeu008863.2 | calmodulin-binding family protein | 5.878751 |
| 27 | Oeu005393.1 | lanceolate | 5.830456 |
| 28 | Oeu013701.3 | b3 domain-containing protein os01g0234100 | 5.818125 |
| 29 | Oeu016514.1 | amine oxidase | 5.818125 |
| 30 | Oeu023081.1 | ramosa 1 enhancer locus 2 | 5.818125 |
| 31 | Oeu028109.1 | aspartate kinase family protein | 5.81192 |
| 32 | Oeu058606.2 | armadillo | 5.713272 |
| 33 | Oeu000153.1 | chloride channel protein | 5.688648 |
| 34 | Oeu029937.1 | glycosyl transferase family 20 family protein | 5.681859 |
| 35 | Oeu046261.1 | endoglucanase | 5.681859 |
| 36 | Oeu028909.1 | at1g23890 t23e23_13 | 5.626364 |
| 37 | Oeu010177.1 | Uncharacterized protein | 5.597795 |
| 38 | Oeu016471.1 | myb-related protein 91 | 5.590563 |
| 39 | Oeu028690.1 |  | 5.590563 |
| 40 | Oeu046276.1 | beta-tubulin | 5.590563 |
| 41 | Oeu003335.2 | wd g-beta repeat protein | 5.583295 |
| 42 | Oeu017214.1 |  | 5.553852 |
| 43 | Oeu023080.1 | aaa-type atpase family protein | 5.553852 |
| 44 | Oeu003433.1 | phosphate-responsive 1 family protein | 5.546396 |
| 45 | Oeu022016.1 | catalase | 5.53561 |
| 46 | Oeu007229.1 | cationic amino acid transporter 5 family protein | 5.531368 |
| 47 | Oeu017986.1 | sucrase-related family protein | 5.531368 |
| 48 | Oeu012144.1 | auxin repressed dormancy associated protein | 5.524554 |
| 49 | Oeu019002.1 | n-acetylglutamate kinase | 5.516181 |
| 50 | Oeu029368.1 | receptor like protein 4 | 5.477501 |
| 51 | Oeu049462.3 | rna-binding domain ccch-type zinc finger protein | 5.469639 |
| 52 | Oeu063557.2 | chain h family protein | 5.461733 |
| 53 | Oeu053624.1 | lim transcription factor | 5.456439 |
| 54 | Oeu043073.2 | dead-box atp-dependent rna helicase | 5.437755 |
| 55 | Oeu038894.1 | lrr receptor-like kinase family protein | 5.421544 |
| 56 | Oeu029731.1 | rna recognition motif-containing family protein | 5.41337 |
| 57 | Oeu035711.1 | basic helix-loop-helix family protein | 5.41337 |
| 58 | Oeu063226.1 | dual specificity protein phosphatase | 5.41337 |
| 59 | Oeu008416.1 | glutamyl-trna reductase | 5.398264 |
| 60 | Oeu048370.1 | octicosapeptide phox bem1p domain-containing family protein | 5.396882 |
| 61 | Oeu009298.1 | lysosomal pro-x carboxypeptidase-like protein | 5.388567 |
| 62 | Oeu022027.2 | alcohol dehydrogenase expressed | 5.371791 |
| 63 | Oeu064519.1 | carboxypeptidase | 5.371791 |
| 64 | Oeu009771.1 | ribose-phosphate pyrophosphokinase | 5.36333 |
| 65 | Oeu035436.1 | rac gtpase activating protein 1 | 5.354818 |
| 66 | Oeu026361.1 | serine threonine-protein phosphatase bsl2 | 5.324626 |
| 67 | Oeu048323.1 | sucrose synthase | 5.324626 |
| 68 | Oeu007168.3 | swib mdm2 domain protein | 5.320261 |
| 69 | Oeu062391.1 | kinase family protein | 5.284856 |
| 70 | Oeu015751.1 | starch chloroplastic amyloplastic | 5.283206 |
| 71 | Oeu039596.1 | serine threonine-protein kinase | 5.275867 |
| 72 | Oeu050775.1 | transducin wd40 repeat protein | 5.275867 |
| 73 | Oeu012190.1 | eukaryotic translation initiation factor 3 subunit l | 5.26076 |
| 74 | Oeu028912.1 | mitochondrial aldehyde dehydrogenase family protein | 5.259545 |
| 75 | Oeu000070.1 | plant f25p12-18 protein | 5.239341 |
| 76 | Oeu005419.1 | choline transporter-related family protein | 5.239341 |
| 77 | Oeu015671.1 | chlorophyllase 2 | 5.239341 |
| 78 | Oeu056669.1 | calmodulin-binding family protein | 5.239341 |
| 79 | Oeu021012.1 | leucine-rich repeat family protein | 5.230063 |
| 80 | Oeu049431.1 |  | 5.230063 |
| 81 | Oeu063554.1 | chain h family protein | 5.230063 |
| 82 | Oeu018757.1 | ketol-acid reductoisomerase | 5.226957 |
| 83 | Oeu051802.2 | ap2 domain-containing transcription factor family protein | 5.211327 |
| 84 | Oeu018431.1 | pollen-specific sf21-like protein | 5.201866 |
| 85 | Oeu057778.2 | dehydration-responsive family protein | 5.201866 |
| 86 | Oeu032039.2 | la-related protein 2 la rna-binding domain protein | 5.192344 |
| 87 | Oeu062201.1 | nsp-interacting kinase-like protein | 5.182758 |
| 88 | Oeu026584.1 | mevalonate disphosphate decarboxylase | 5.173107 |
| 89 | Oeu055658.1 | subtilisin-like serine protease | 5.173107 |
| 90 | Oeu005601.1 | phd finger and bromo-adjacent homology domain-containing protein | 5.163392 |
| 91 | Oeu038306.1 | basic helix-loop-helix family protein | 5.163392 |
| 92 | Oeu055602.1 | aaa-type atpase family protein | 5.143763 |
| 93 | Oeu062369.1 | pmr5 cas1p gdsl sgnh-like acyl-esterase family protein | 5.143763 |
| 94 | Oeu027266.1 | zip zinc iron transport family protein | 5.138814 |
| 95 | Oeu016029.1 | hexosyltransferase | 5.133848 |
| 96 | Oeu052794.1 | lutescent 2 | 5.133848 |
| 97 | Oeu063392.1 | pyruvate decarboxylase 1 | 5.133848 |
| 98 | Oeu033245.1 | beta-hexosaminidase | 5.123864 |
| 99 | Oeu051950.1 | pyridine nucleotide-disulfide oxidoreductase domain protein | 5.123864 |
| 100 | Oeu000741.3 |  | 5.11381 |
